# Supplementary material for: COVID-19 disease—Temporal analyses of complete blood count parameters over course of illness, and relationship to patient demographics and management outcomes in survivors and non-survivors: A longitudinal descriptive cohort study
Source: PLoS One. 2020 Dec 28;15(12):e0244129. doi: 10.1371/journal.pone.0244129 (PMC7769441; doi:10.1371/journal.pone.0244129)
Supplement: S1 File — (PDF) [file pone.0244129.s001.pdf]

```
      name:  <unnamed>
       log:  /Users/Chiara/Documents/fileDO 15 Maggio/Kinetics Hematological .smcl
  log type:  smcl
 opened on:  19 May 2020, 16:47:16

  1 . do "/Users/Chiara/Documents/fileDO 15 Maggio/analisi_coorte_wide_multilevel_4.do"

  2 . use "/Users/Chiara/Documents/fileDO 15 Maggio/fileDo\coorte_long_emocromo_anemia0.dta", clear

  3 . *****  etichette
  4 .
  5 . replace death=0 if death==99 /* censura dei ricoveray */
    (506 real changes made)

  6 . gen death_30=death

  7 . gen death_time=a_data_esito-a_data_sintomi if death_30==1
    (9,438 missing values generated)

  8 .
  9 . replace death_30=0 if death_time>=30
    (286 real changes made)

 10 . replace a_data_ricovero=a_data_test if a_data_ricovero<a_data_sintomi
    (242 real changes made)

 11 . *gen inizio=mdy(02,29,2020)
 12 . *gen giorno_x=a_data_ricovero-inizio
 13 . *recode giorno_x -199/0=0 1/7=1 8/14=2 15/21=3 22/28=4 29/2000=99, into(settimana)
 14 . tab settimana, mi
```

RECODE of giorno_x	Freq.	Percent	Cum.
0	132	1.24	1.24
1	1,496	14.02	15.26
2	2,024	18.97	34.23
3	3,234	30.31	64.54
4	2,310	21.65	86.19
99	1,474	13.81	100.00
Total	10,670	100.00	

```
 15 . drop if a_data_ricovero>=mdy(03,29,2020)
    (1,474 observations deleted)

 16 . tab settimana, mi
```

RECODE of giorno_x	Freq.	Percent	Cum.
0	132	1.44	1.44
1	1,496	16.27	17.70
2	2,024	22.01	39.71
3	3,234	35.17	74.88
4	2,310	25.12	100.00
Total	9,196	100.00	

```
 17 . keep if dropping==0
    (660 observations deleted)

 18 . tab settimana
```

RECODE of giorno_x	Freq.	Percent	Cum.
0	132	1.55	1.55
1	1,364	15.98	17.53
2	1,980	23.20	40.72
3	2,992	35.05	75.77
4	2,068	24.23	100.00
Total	8,536	100.00	

```
 19 . recode p_age 0/59=0 60/700=1, into(p_age2)
    (8536 differences between p_age and p_age2)

 20 . tab death_30
```

death_30	Freq.	Percent	Cum.
0	7,612	89.18	89.18
1	924	10.82	100.00
Total	8,536	100.00	

```
 21 .
 22 . label define death_30 1 "nonsurvivor" 0 "survivor"

 23 . label values death_30 death_30

 24 . label variable b_day_malattia "Days since onset"

 25 .
 26 . save "/Users/Chiara/Documents/fileDO 15 Maggio/fileDo\coorte_long_emocromo_anemia_1.dta", replace
    file /Users/Chiara/Documents/fileDO 15 Maggio/fileDo\coorte_long_emocromo_anemia_1.dta saved

 27 .
 28 .
 29 . ****************************************************************************
 30 . *** analisi leucociti  ****************************************************
 31 . ****************************************************************************
 32 . **** scatter no model
 33 . *use "/Users/Chiara/Documents/fileDO 15 Maggio/fileDO\coorte_long_emocromo_anemia_1.dta", clear
 34 . *scatter wbc b_day_malattia  if death_30==1, mcolor(red) legend(off) title("WBC variation over time") xtitle("day
    > since onset") ytitle("WBC per mmc X 1000") || scatter wbc b_day_malattia  if death_30==0, mcolor(green) legend(off
```

```
   > ) ms(oh)
35 . *keep if wbc!=.
36 . *sort a_data_test
37 . *collapse (first) wbc (first) death (first) b_day_malattia, by(progr)
38 .
39 .
40 .
41 . **** modelling
42 . *use "/Users/Chiara/Documents/fileDO 15 Maggio/fileDO\coorte_long_emocromo_anemia_1.dta", clear
43 . *mixed wbc_ln c.b_day_malattia##i.death_30 i.p_age2 i.p_obeso i.p_rene i.p_cardio || progr:b_day_malattia, cov(uns
   > tr)
44 . *est store linear
45 . *mixed wbc_ln  c.b_day_malattia##c.b_day_malattia##i.death_30 i.p_age2 i.p_obeso i.p_rene i.p_cardio || progr:b_da
   > y_malattia, cov(unstr)
46 . *est store quadratic
47 . *lrtest linear quadratic
48 . ****** plotting and estimates
49 . *quiet: mixed wbc_ln  c.b_day_malattia##c.b_day_malattia##i.death_30 i.p_age2 i.p_obeso i.p_rene i.p_cardio || pro
   > gr:b_day_malattia, cov(unstr)
50 .
51 . * death_30, at(b_day_malattia=(0(1)30))  expression(exp(predict(xb))) asbal
52 . *marginsplot, title("WBC kinetic day 0 to day 30 after onset") ytitle("Cells per mmc (log-scale)")
53 . *margins ar.death_30, at(b_day_malattia=(0(1)30))  expression(exp(predict(xb))) asbal
54 . *margins, at(b_day_malattia=(0(1)30))  expression(exp(predict(xb))) contrast
55 . *margins, at(b_day_malattia=(0(1)30) death==0)  expression(exp(predict(xb)))  contrast
56 .
57 .
58 . ***************************************************************************
59 . *** analisi neutrofili  **************************************************
60 . ***************************************************************************
61 . **** scatter no model
62 . use "/Users/Chiara/Documents/fileDO 15 Maggio/fileDO\coorte_long_emocromo_anemia_1.dta", clear

63 . *scatter neu b_day_malattia  if death==1, mcolor(red) legend(off) title("Neutrophils variation over time") xtitle(
   > "day since onset") ytitle("Neutrophils per mmc X 1000")xlab(0(5)30) || scatter neu b_day_malattia  if death==0, mc
   > olor(green) legend(off) ms(oh)
64 . *********Graph box
65 . replace neu=neu*1000
   (1,805 real changes made)

66 . graph box neu, over(death_30, label(nolabel)) over(b_day_malattia) asyvars box(1, fcolor(navy))  nooutsides ytitle
   > (Cells per mmc) yline(500, lcolor(red)) legend(off)

67 . graph save "Graph" "/Users/Chiara/Documents/fileDO 15 Maggio/BOX neu .gph", replace
   (file /Users/Chiara/Documents/fileDO 15 Maggio/BOX neu .gph saved)

68 . **** modelling
69 . use "/Users/Chiara/Documents/fileDO 15 Maggio/fileDO\coorte_long_emocromo_anemia_1.dta", clear

70 . mixed neu_ln c.b_day_malattia##i.death_30 i.p_age2 i.p_obeso i.p_rene|| progr:b_day_malattia, cov(unstr)

   Performing EM optimization:

   Performing gradient-based optimization:

   Iteration 0:   log likelihood = -1197.7645
   Iteration 1:   log likelihood = -1197.7498
   Iteration 2:   log likelihood = -1197.7498

   Computing standard errors:

   Mixed-effects ML regression                     Number of obs     =      1,805
   Group variable: progr                           Number of groups  =        379

                                                   Obs per group:
                                                                 min =          1
                                                                 avg =        4.8
                                                                 max =         22

                                                   Wald chi2(6)      =     120.65
   Log likelihood = -1197.7498                     Prob > chi2       =     0.0000
```

neu_ln	Coef.	Std. Err.	z	P>|z|	[95% Conf.	Interval]
b_day_malattia	.026355	.0037435	7.04	0.000	.0190179	.0336921
death_30						
nonsurvivor	-.0692439	.1417194	-0.49	0.625	-.3470088	.208521
death_30#c.b_day_malattia						
nonsurvivor	.0442918	.0110632	4.00	0.000	.0226084	.0659752
1.p_age2	.1332044	.0559794	2.38	0.017	.0234869	.2429219
1.p_obeso	.1115497	.1115481	1.00	0.317	-.1070805	.33018
1.p_rene	.0614802	.127466	0.48	0.630	-.1883485	.3113089
_cons	8.035981	.0590407	136.11	0.000	7.920263	8.151698

Random-effects Parameters	Estimate	Std. Err.	[95% Conf.	Interval]
progr: Unstructured				
var(b_day_malattia)	.0020646	.000305	.0015456	.0027579
var(_cons)	.4650455	.0565429	.3664388	.5901866
cov(b_day_malattia,_cons)	-.0225641	.0038667	-.0301428	-.0149854
var(Residual)	.1239949	.0050842	.1144201	.1343711

```
   LR test vs. linear model: chi2(3) = 1078.06               Prob > chi2 = 0.0000

   Note: LR test is conservative and provided only for reference.

71 . est store linear

72 . mixed neu_ln  c.b_day_malattia##c.b_day_malattia##i.death_30 i.p_age2 i.p_cardio i.p_obeso i.p_rene || progr:b_day
   > _malattia, cov(unstr)
```

```
Performing EM optimization:

Performing gradient-based optimization:

Iteration 0:   log likelihood = -1188.5729
Iteration 1:   log likelihood = -1188.5576
Iteration 2:   log likelihood = -1188.5576

Computing standard errors:

Mixed-effects ML regression                     Number of obs     =      1,805
Group variable: progr                           Number of groups  =        379

                                                Obs per group:
                                                              min =          1
                                                              avg =        4.8
                                                              max =         22

                                                Wald chi2(9)      =     137.27
Log likelihood = -1188.5576                     Prob > chi2       =     0.0000

------------------------------------------------------------------------------------------------------------
                                     neu_ln |      Coef.   Std. Err.      z    P>|z|     [95% Conf. Interval]
--------------------------------------------+---------------------------------------------------------------
                              b_day_malattia |   .0674394   .0106074     6.36   0.000     .0466493    .0882295
                                             |
        c.b_day_malattia#c.b_day_malattia    |   -.001686   .0004058    -4.16   0.000    -.0024813   -.0008907
                                             |
                                    death_30 |
                                nonsurvivor  |   .2550166   .1800781     1.42   0.157    -.0979301    .6079633
                                             |
                     death_30#c.b_day_malattia |
                                nonsurvivor  |  -.0224537   .0269067    -0.83   0.404    -.0751899    .0302825
                                             |
death_30#c.b_day_malattia#c.b_day_malattia   |
                                nonsurvivor  |   .0028729   .0011111     2.59   0.010     .0006951    .0050507
                                             |
                                    1.p_age2 |   .1306073   .0591056     2.21   0.027     .0147625    .2464522
                                  1.p_cardio |   .0168001   .0632414     0.27   0.791    -.1071507    .1407509
                                   1.p_obeso |    .107481     .11163     0.96   0.336    -.1113099    .3262718
                                    1.p_rene |   .0578037   .1291291     0.45   0.654    -.1952846     .310892
                                       _cons |   7.814722   .0796682    98.09   0.000     7.658575    7.970868
------------------------------------------------------------------------------------------------------------

------------------------------------------------------------------------------
  Random-effects Parameters  |   Estimate   Std. Err.     [95% Conf. Interval]
-----------------------------+------------------------------------------------
progr: Unstructured          |
         var(b_day_malattia) |   .0021292   .0003116      .0015983    .0028365
                  var(_cons) |   .4650909   .0567559      .3661542    .5907607
   cov(b_day_malattia,_cons) |  -.0229141   .0039075     -.0305726   -.0152555
-----------------------------+------------------------------------------------
               var(Residual) |   .1218045   .0050145      .1123623    .1320402
------------------------------------------------------------------------------
LR test vs. linear model: chi2(3) = 1089.99               Prob > chi2 = 0.0000

Note: LR test is conservative and provided only for reference.

 73 . est store quadratic

 74 . lrtest linear quadratic

Likelihood-ratio test                                 LR chi2(3)  =     18.38
(Assumption: linear nested in quadratic)              Prob > chi2 =    0.0004

 75 . **** margins
 76 . quiet: mixed neu_ln  c.b_day_malattia##c.b_day_malattia##i.death_30 i.p_age2 i.p_cardio i.p_obeso i.p_rene || prog
> r:b_day_malattia, cov(unstr)

 77 . margins death_30, at(b_day_malattia=(0(1)21))  expression(exp(predict(xb))) asbal

Adjusted predictions                            Number of obs     =      1,805

Expression   : exp(predict(xb))

1._at        : b_day_mala~a    =           0
               death_30                      (asbalanced)
               p_age2                        (asbalanced)
               p_cardio                      (asbalanced)
               p_obeso                       (asbalanced)
               p_rene                        (asbalanced)

2._at        : b_day_mala~a    =           1
               death_30                      (asbalanced)
               p_age2                        (asbalanced)
               p_cardio                      (asbalanced)
               p_obeso                       (asbalanced)
               p_rene                        (asbalanced)

3._at        : b_day_mala~a    =           2
               death_30                      (asbalanced)
               p_age2                        (asbalanced)
               p_cardio                      (asbalanced)
               p_obeso                       (asbalanced)
               p_rene                        (asbalanced)

4._at        : b_day_mala~a    =           3
               death_30                      (asbalanced)
               p_age2                        (asbalanced)
               p_cardio                      (asbalanced)
               p_obeso                       (asbalanced)
               p_rene                        (asbalanced)

5._at        : b_day_mala~a    =           4
               death_30                      (asbalanced)
               p_age2                        (asbalanced)
               p_cardio                      (asbalanced)
               p_obeso                       (asbalanced)
```

```
              p_rene                          (asbalanced)

      6._at  : b_day_mala~a    =          5
              death_30                        (asbalanced)
              p_age2                          (asbalanced)
              p_cardio                        (asbalanced)
              p_obeso                         (asbalanced)
              p_rene                          (asbalanced)

      7._at  : b_day_mala~a    =          6
              death_30                        (asbalanced)
              p_age2                          (asbalanced)
              p_cardio                        (asbalanced)
              p_obeso                         (asbalanced)
              p_rene                          (asbalanced)

      8._at  : b_day_mala~a    =          7
              death_30                        (asbalanced)
              p_age2                          (asbalanced)
              p_cardio                        (asbalanced)
              p_obeso                         (asbalanced)
              p_rene                          (asbalanced)

      9._at  : b_day_mala~a    =          8
              death_30                        (asbalanced)
              p_age2                          (asbalanced)
              p_cardio                        (asbalanced)
              p_obeso                         (asbalanced)
              p_rene                          (asbalanced)

     10._at  : b_day_mala~a    =          9
              death_30                        (asbalanced)
              p_age2                          (asbalanced)
              p_cardio                        (asbalanced)
              p_obeso                         (asbalanced)
              p_rene                          (asbalanced)

     11._at  : b_day_mala~a    =         10
              death_30                        (asbalanced)
              p_age2                          (asbalanced)
              p_cardio                        (asbalanced)
              p_obeso                         (asbalanced)
              p_rene                          (asbalanced)

     12._at  : b_day_mala~a    =         11
              death_30                        (asbalanced)
              p_age2                          (asbalanced)
              p_cardio                        (asbalanced)
              p_obeso                         (asbalanced)
              p_rene                          (asbalanced)

     13._at  : b_day_mala~a    =         12
              death_30                        (asbalanced)
              p_age2                          (asbalanced)
              p_cardio                        (asbalanced)
              p_obeso                         (asbalanced)
              p_rene                          (asbalanced)

     14._at  : b_day_mala~a    =         13
              death_30                        (asbalanced)
              p_age2                          (asbalanced)
              p_cardio                        (asbalanced)
              p_obeso                         (asbalanced)
              p_rene                          (asbalanced)

     15._at  : b_day_mala~a    =         14
              death_30                        (asbalanced)
              p_age2                          (asbalanced)
              p_cardio                        (asbalanced)
              p_obeso                         (asbalanced)
              p_rene                          (asbalanced)

     16._at  : b_day_mala~a    =         15
              death_30                        (asbalanced)
              p_age2                          (asbalanced)
              p_cardio                        (asbalanced)
              p_obeso                         (asbalanced)
              p_rene                          (asbalanced)

     17._at  : b_day_mala~a    =         16
              death_30                        (asbalanced)
              p_age2                          (asbalanced)
              p_cardio                        (asbalanced)
              p_obeso                         (asbalanced)
              p_rene                          (asbalanced)

     18._at  : b_day_mala~a    =         17
              death_30                        (asbalanced)
              p_age2                          (asbalanced)
              p_cardio                        (asbalanced)
              p_obeso                         (asbalanced)
              p_rene                          (asbalanced)

     19._at  : b_day_mala~a    =         18
              death_30                        (asbalanced)
              p_age2                          (asbalanced)
              p_cardio                        (asbalanced)
              p_obeso                         (asbalanced)
              p_rene                          (asbalanced)

     20._at  : b_day_mala~a    =         19
              death_30                        (asbalanced)
              p_age2                          (asbalanced)
              p_cardio                        (asbalanced)
              p_obeso                         (asbalanced)
              p_rene                          (asbalanced)

     21._at  : b_day_mala~a    =         20
```

```
             death_30                      (asbalanced)
             p_age2                        (asbalanced)
             p_cardio                      (asbalanced)
             p_obeso                       (asbalanced)
             p_rene                        (asbalanced)

22._at     : b_day_mala~a    =          21
             death_30                      (asbalanced)
             p_age2                        (asbalanced)
             p_cardio                      (asbalanced)
             p_obeso                       (asbalanced)
             p_rene                        (asbalanced)
```

	Margin	Delta-method Std. Err.	z	P>|z|	[95% Conf.	Interval]
_at#death_30						
1#survivor	2895.947	315.9751	9.17	0.000	2276.648	3515.247
1#nonsurvivor	3737.171	637.8243	5.86	0.000	2487.058	4987.284
2#survivor	3092.766	319.527	9.68	0.000	2466.504	3719.027
2#nonsurvivor	3913.772	599.8806	6.52	0.000	2738.027	5089.516
3#survivor	3291.842	324.3425	10.15	0.000	2656.142	3927.541
3#nonsurvivor	4108.458	569.5686	7.21	0.000	2992.124	5224.792
4#survivor	3491.937	330.6288	10.56	0.000	2843.917	4139.958
4#nonsurvivor	4323.079	547.9801	7.89	0.000	3249.058	5397.101
5#survivor	3691.726	338.4798	10.91	0.000	3028.318	4355.135
5#nonsurvivor	4559.723	536.1051	8.51	0.000	3508.976	5610.469
6#survivor	3889.808	347.8639	11.18	0.000	3208.007	4571.609
6#nonsurvivor	4820.75	534.6489	9.02	0.000	3772.857	5868.642
7#survivor	4084.721	358.6288	11.39	0.000	3381.821	4787.62
7#nonsurvivor	5108.832	543.8963	9.39	0.000	4042.815	6174.849
8#survivor	4274.961	370.5211	11.54	0.000	3548.753	5001.169
8#nonsurvivor	5426.997	563.7055	9.63	0.000	4322.155	6531.84
9#survivor	4459	383.217	11.64	0.000	3707.909	5210.092
9#nonsurvivor	5778.678	593.6558	9.73	0.000	4615.134	6942.222
10#survivor	4635.306	396.359	11.69	0.000	3858.457	5412.156
10#nonsurvivor	6167.771	633.305	9.74	0.000	4926.516	7409.026
11#survivor	4802.363	409.5925	11.72	0.000	3999.576	5605.149
11#nonsurvivor	6598.709	682.4841	9.67	0.000	5261.064	7936.353
12#survivor	4958.691	422.601	11.73	0.000	4130.408	5786.973
12#nonsurvivor	7076.533	741.5821	9.54	0.000	5623.059	8530.007
13#survivor	5102.872	435.1362	11.73	0.000	4250.021	5955.723
13#nonsurvivor	7606.994	811.8171	9.37	0.000	6015.862	9198.126
14#survivor	5233.568	447.0432	11.71	0.000	4357.38	6109.757
14#nonsurvivor	8196.652	895.5072	9.15	0.000	6441.49	9951.814
15#survivor	5349.543	458.2804	11.67	0.000	4451.33	6247.756
15#nonsurvivor	8853.008	996.3517	8.89	0.000	6900.194	10805.82
16#survivor	5449.681	468.9322	11.62	0.000	4530.591	6368.771
16#nonsurvivor	9584.646	1119.711	8.56	0.000	7390.053	11779.24
17#survivor	5533.005	479.2139	11.55	0.000	4593.763	6472.247
17#nonsurvivor	10401.41	1272.861	8.17	0.000	7906.648	12896.17
18#survivor	5598.692	489.4664	11.44	0.000	4639.355	6558.028
18#nonsurvivor	11314.6	1465.197	7.72	0.000	8442.869	14186.34
19#survivor	5646.088	500.1386	11.29	0.000	4665.834	6626.342
19#nonsurvivor	12337.22	1708.388	7.22	0.000	8988.841	15685.6
20#survivor	5674.718	511.7552	11.09	0.000	4671.697	6677.74
20#nonsurvivor	13484.23	2016.536	6.69	0.000	9531.893	17436.57
21#survivor	5684.294	524.8707	10.83	0.000	4655.567	6713.022
21#nonsurvivor	14772.91	2406.426	6.14	0.000	10056.4	19489.42
22#survivor	5674.719	540.0129	10.51	0.000	4616.313	6733.125
22#nonsurvivor	16223.21	2897.934	5.60	0.000	10543.36	21903.05

```
78 . marginsplot, ytitle("Cells per mmc") xlab(0(1)21) yline(500, lcolor(red)) title ("") legend(off)

     Variables that uniquely identify margins: b_day_malattia death_30

79 . margins ar.death_30, at(b_day_malattia=(0(1)21))  expression(exp(predict(xb))) asbal

   Contrasts of adjusted predictions               Number of obs     =      1,805

   Expression    : exp(predict(xb))

   1._at         : b_day_mala~a    =           0
                   death_30                      (asbalanced)
                   p_age2                        (asbalanced)
                   p_cardio                      (asbalanced)
                   p_obeso                       (asbalanced)
                   p_rene                        (asbalanced)

   2._at         : b_day_mala~a    =           1
                   death_30                      (asbalanced)
                   p_age2                        (asbalanced)
                   p_cardio                      (asbalanced)
                   p_obeso                       (asbalanced)
                   p_rene                        (asbalanced)

   3._at         : b_day_mala~a    =           2
                   death_30                      (asbalanced)
                   p_age2                        (asbalanced)
                   p_cardio                      (asbalanced)
                   p_obeso                       (asbalanced)
                   p_rene                        (asbalanced)

   4._at         : b_day_mala~a    =           3
                   death_30                      (asbalanced)
                   p_age2                        (asbalanced)
                   p_cardio                      (asbalanced)
                   p_obeso                       (asbalanced)
                   p_rene                        (asbalanced)

   5._at         : b_day_mala~a    =           4
                   death_30                      (asbalanced)
                   p_age2                        (asbalanced)
                   p_cardio                      (asbalanced)
                   p_obeso                       (asbalanced)
                   p_rene                        (asbalanced)
```

```
6._at        : b_day_mala~a    =          5
               death_30                     (asbalanced)
               p_age2                       (asbalanced)
               p_cardio                     (asbalanced)
               p_obeso                      (asbalanced)
               p_rene                       (asbalanced)

7._at        : b_day_mala~a    =          6
               death_30                     (asbalanced)
               p_age2                       (asbalanced)
               p_cardio                     (asbalanced)
               p_obeso                      (asbalanced)
               p_rene                       (asbalanced)

8._at        : b_day_mala~a    =          7
               death_30                     (asbalanced)
               p_age2                       (asbalanced)
               p_cardio                     (asbalanced)
               p_obeso                      (asbalanced)
               p_rene                       (asbalanced)

9._at        : b_day_mala~a    =          8
               death_30                     (asbalanced)
               p_age2                       (asbalanced)
               p_cardio                     (asbalanced)
               p_obeso                      (asbalanced)
               p_rene                       (asbalanced)

10._at       : b_day_mala~a    =          9
               death_30                     (asbalanced)
               p_age2                       (asbalanced)
               p_cardio                     (asbalanced)
               p_obeso                      (asbalanced)
               p_rene                       (asbalanced)

11._at       : b_day_mala~a    =         10
               death_30                     (asbalanced)
               p_age2                       (asbalanced)
               p_cardio                     (asbalanced)
               p_obeso                      (asbalanced)
               p_rene                       (asbalanced)

12._at       : b_day_mala~a    =         11
               death_30                     (asbalanced)
               p_age2                       (asbalanced)
               p_cardio                     (asbalanced)
               p_obeso                      (asbalanced)
               p_rene                       (asbalanced)

13._at       : b_day_mala~a    =         12
               death_30                     (asbalanced)
               p_age2                       (asbalanced)
               p_cardio                     (asbalanced)
               p_obeso                      (asbalanced)
               p_rene                       (asbalanced)

14._at       : b_day_mala~a    =         13
               death_30                     (asbalanced)
               p_age2                       (asbalanced)
               p_cardio                     (asbalanced)
               p_obeso                      (asbalanced)
               p_rene                       (asbalanced)

15._at       : b_day_mala~a    =         14
               death_30                     (asbalanced)
               p_age2                       (asbalanced)
               p_cardio                     (asbalanced)
               p_obeso                      (asbalanced)
               p_rene                       (asbalanced)

16._at       : b_day_mala~a    =         15
               death_30                     (asbalanced)
               p_age2                       (asbalanced)
               p_cardio                     (asbalanced)
               p_obeso                      (asbalanced)
               p_rene                       (asbalanced)

17._at       : b_day_mala~a    =         16
               death_30                     (asbalanced)
               p_age2                       (asbalanced)
               p_cardio                     (asbalanced)
               p_obeso                      (asbalanced)
               p_rene                       (asbalanced)

18._at       : b_day_mala~a    =         17
               death_30                     (asbalanced)
               p_age2                       (asbalanced)
               p_cardio                     (asbalanced)
               p_obeso                      (asbalanced)
               p_rene                       (asbalanced)

19._at       : b_day_mala~a    =         18
               death_30                     (asbalanced)
               p_age2                       (asbalanced)
               p_cardio                     (asbalanced)
               p_obeso                      (asbalanced)
               p_rene                       (asbalanced)

20._at       : b_day_mala~a    =         19
               death_30                     (asbalanced)
               p_age2                       (asbalanced)
               p_cardio                     (asbalanced)
               p_obeso                      (asbalanced)
               p_rene                       (asbalanced)

21._at       : b_day_mala~a    =         20
               death_30                     (asbalanced)
```

```
              p_age2                       (asbalanced)
              p_cardio                     (asbalanced)
              p_obeso                      (asbalanced)
              p_rene                       (asbalanced)

22._at       : b_day_mala~a    =          21
              death_30                     (asbalanced)
              p_age2                       (asbalanced)
              p_cardio                     (asbalanced)
              p_obeso                      (asbalanced)
              p_rene                       (asbalanced)
```

	df	chi2	P>chi2
death_30@_at			
(nonsurvivor vs survivor) 1	1	1.71	0.1908
(nonsurvivor vs survivor) 2	1	1.88	0.1701
(nonsurvivor vs survivor) 3	1	2.13	0.1449
(nonsurvivor vs survivor) 4	1	2.46	0.1167
(nonsurvivor vs survivor) 5	1	2.91	0.0878
(nonsurvivor vs survivor) 6	1	3.50	0.0612
(nonsurvivor vs survivor) 7	1	4.24	0.0394
(nonsurvivor vs survivor) 8	1	5.14	0.0234
(nonsurvivor vs survivor) 9	1	6.19	0.0128
(nonsurvivor vs survivor) 10	1	7.42	0.0065
(nonsurvivor vs survivor) 11	1	8.81	0.0030
(nonsurvivor vs survivor) 12	1	10.36	0.0013
(nonsurvivor vs survivor) 13	1	12.02	0.0005
(nonsurvivor vs survivor) 14	1	13.70	0.0002
(nonsurvivor vs survivor) 15	1	15.25	0.0001
(nonsurvivor vs survivor) 16	1	16.52	0.0000
(nonsurvivor vs survivor) 17	1	17.34	0.0000
(nonsurvivor vs survivor) 18	1	17.61	0.0000
(nonsurvivor vs survivor) 19	1	17.32	0.0000
(nonsurvivor vs survivor) 20	1	16.54	0.0000
(nonsurvivor vs survivor) 21	1	15.40	0.0001
(nonsurvivor vs survivor) 22	1	14.05	0.0002
Joint	6	161.15	0.0000

	Contrast	Delta-method Std. Err.	[95% Conf.	Interval]
death_30@_at				
(nonsurvivor vs survivor) 1	841.2236	643.0631	-419.157	2101.604
(nonsurvivor vs survivor) 2	821.0059	598.3877	-351.8125	1993.824
(nonsurvivor vs survivor) 3	816.6166	560.1934	-281.3422	1914.575
(nonsurvivor vs survivor) 4	831.1421	529.7621	-207.1726	1869.457
(nonsurvivor vs survivor) 5	867.9964	508.4221	-128.4925	1864.485
(nonsurvivor vs survivor) 6	930.9419	497.3304	-43.80777	1905.692
(nonsurvivor vs survivor) 7	1024.112	497.2321	49.55461	1998.668
(nonsurvivor vs survivor) 8	1152.036	508.3174	155.7526	2148.32
(nonsurvivor vs survivor) 9	1319.677	530.289	280.33	2359.025
(nonsurvivor vs survivor) 10	1532.465	562.6437	429.7035	2635.226
(nonsurvivor vs survivor) 11	1796.346	605.0691	610.4325	2982.26
(nonsurvivor vs survivor) 12	2117.843	657.8449	828.4903	3407.195
(nonsurvivor vs survivor) 13	2504.122	722.1985	1088.639	3919.605
(nonsurvivor vs survivor) 14	2963.084	800.6131	1393.911	4532.257
(nonsurvivor vs survivor) 15	3503.464	897.0964	1745.188	5261.741
(nonsurvivor vs survivor) 16	4134.965	1017.401	2140.896	6129.035
(nonsurvivor vs survivor) 17	4868.406	1169.179	2576.857	7159.955
(nonsurvivor vs survivor) 18	5715.911	1362.073	3046.298	8385.524
(nonsurvivor vs survivor) 19	6691.131	1607.788	3539.924	9842.338
(nonsurvivor vs survivor) 20	7809.513	1920.24	4045.912	11573.11
(nonsurvivor vs survivor) 21	9088.613	2315.847	4549.637	13627.59
(nonsurvivor vs survivor) 22	10548.49	2814.037	5033.076	16063.9

```
 80 . graph save "Graph" "/Users/Chiara/Documents/fileDO 15 Maggio/Marg_NEU.gph", replace
    (file /Users/Chiara/Documents/fileDO 15 Maggio/Marg_NEU.gph saved)

 81 . ***combine*****
 82 . gr combine "/Users/Chiara/Documents/fileDO 15 Maggio/Marg_NEU.gph" "/Users/Chiara/Documents/fileDO 15 Maggio/BOX n
    > eu .gph", ycomm   xsize(8) ysize(4)

 83 . graph save "Graph" "/Users/Chiara/Documents/fileDO 15 Maggio/Graph_NEU.gph", replace
    (file /Users/Chiara/Documents/fileDO 15 Maggio/Graph_NEU.gph saved)

 84 .
 85 .
 86 . ***************************************************************************
 87 . *** analisi Lymphocytes  **************************************************
 88 . ***************************************************************************
 89 . **** scatter no model
 90 . use "/Users/Chiara/Documents/fileDO 15 Maggio/fileDO\coorte_long_emocromo_anemia_1.dta", clear

 91 . *scatter lym b_day_malattia  if death_30==1, mcolor(red) legend(off) title("Lymphocites variation over time") xtit
    > le("day since onset") ytitle("Cells per mmc X 1000") xlab(0(5)30)|| scatter lym b_day_malattia  if death_30==0, mc
    > olor(green) legend(off) ms(oh)
 92 . *keep if lym!=.
 93 . *********Graph box**************************************
 94 . replace lym=lym*1000
    (1,805 real changes made)

 95 . graph box lym, over(death_30, label(nolabel)) over(b_day_malattia) asyvars box(1, fcolor(navy))  nooutsides ytitle
    > (Cells per mmc) yline(200, lcolor(red)) legend(off)

 96 . graph save "Graph" "/Users/Chiara/Documents/fileDO 15 Maggio/BOX_LYM.gph", replace
    (file /Users/Chiara/Documents/fileDO 15 Maggio/BOX_LYM.gph saved)

 97 .
 98 . **** modelling
 99 . use "/Users/Chiara/Documents/fileDO 15 Maggio/fileDO\coorte_long_emocromo_anemia_1.dta", clear

100 . mixed lym_ln c.b_day_malattia##i.death_30 i.p_age2 i.p_cardio i.p_obeso i.p_rene || progr:b_day_malattia, cov(unst
    > r)
```

```
    Performing EM optimization:

    Performing gradient-based optimization:

    Iteration 0:   log likelihood = -1068.4684
    Iteration 1:   log likelihood =  -1068.451
    Iteration 2:   log likelihood =  -1068.451

    Computing standard errors:

    Mixed-effects ML regression                     Number of obs     =      1,805
    Group variable: progr                           Number of groups  =        379

                                                    Obs per group:
                                                                  min =          1
                                                                  avg =        4.8
                                                                  max =         22

                                                    Wald chi2(7)      =     103.79
    Log likelihood =  -1068.451                     Prob > chi2       =     0.0000

    ------------------------------------------------------------------------------------------
                              lym_ln |      Coef.   Std. Err.      z    P>|z|     [95% Conf. Interval]
    ---------------------------------+--------------------------------------------------------
                       b_day_malattia |   .0156949   .0030565     5.13   0.000     .0097043    .0216854
                                      |
                             death_30 |
                          nonsurvivor |  -.1994586   .1093057    -1.82   0.068    -.4136939    .0147766
                                      |
        death_30#c.b_day_malattia     |
                          nonsurvivor |  -.0293182   .0090334    -3.25   0.001    -.0470234    -.011613
                                      |
                             1.p_age2 |  -.2179975   .0536462    -4.06   0.000    -.3231422   -.1128528
                           1.p_cardio |   .0118107    .057423     0.21   0.837    -.1007363    .1243577
                            1.p_obeso |  -.0057988   .1010762    -0.06   0.954    -.2039045    .1923069
                             1.p_rene |  -.2148718   .1154012    -1.86   0.063     -.441054    .0113104
                                _cons |   6.908192   .0489655   141.08   0.000     6.812222    7.004163
    ------------------------------------------------------------------------------------------

    ------------------------------------------------------------------------------
      Random-effects Parameters  |   Estimate   Std. Err.     [95% Conf. Interval]
    -----------------------------+------------------------------------------------
    progr: Unstructured          |
             var(b_day_malattia) |   .0010352   .0001846      .0007298    .0014684
                       var(_cons) |   .2167147   .0315218      .1629591    .2882027
       cov(b_day_malattia,_cons) |  -.0071685   .0020917     -.0112683   -.0030688
    -----------------------------+------------------------------------------------
                    var(Residual) |    .115312   .0046531      .1065433    .1248023
    ------------------------------------------------------------------------------
    LR test vs. linear model: chi2(3) = 1070.45               Prob > chi2 = 0.0000

    Note: LR test is conservative and provided only for reference.

101 . est store linear

102 . mixed lym_ln  c.b_day_malattia##c.b_day_malattia##i.death_30 i.p_age2 i.p_cardio i.p_obeso i.p_rene || progr:b_day
    > _malattia, cov(unstr)

    Performing EM optimization:

    Performing gradient-based optimization:

    Iteration 0:   log likelihood =  -1002.984
    Iteration 1:   log likelihood =  -1002.959
    Iteration 2:   log likelihood =  -1002.959

    Computing standard errors:

    Mixed-effects ML regression                     Number of obs     =      1,805
    Group variable: progr                           Number of groups  =        379

                                                    Obs per group:
                                                                  min =          1
                                                                  avg =        4.8
                                                                  max =         22

                                                    Wald chi2(9)      =     246.54
    Log likelihood =  -1002.959                     Prob > chi2       =     0.0000

    -------------------------------------------------------------------------------------------------------
                                              lym_ln |      Coef.   Std. Err.      z    P>|z|     [95% Conf. Interval]
    -------------------------------------------------+-----------------------------------------------------
                                       b_day_malattia |  -.0774067   .0091223    -8.49   0.000     -.095286   -.0595274
                                                      |
                      c.b_day_malattia#c.b_day_malattia |   .0038736   .0003553    10.90   0.000     .0031772      .00457
                                                      |
                                             death_30 |
                                          nonsurvivor |  -.2860727   .1407578    -2.03   0.042    -.5619529   -.0101925
                                                      |
                            death_30#c.b_day_malattia |
                                          nonsurvivor |  -.0281842   .0233644    -1.21   0.228    -.0739777    .0176093
                                                      |
        death_30#c.b_day_malattia#c.b_day_malattia    |
                                          nonsurvivor |   .0002729   .0009716     0.28   0.779    -.0016315    .0021773
                                                      |
                                             1.p_age2 |  -.2218182   .0523814    -4.23   0.000    -.3244839   -.1191526
                                           1.p_cardio |   .0097151   .0560636     0.17   0.862    -.1001675    .1195977
                                            1.p_obeso |   .0367953   .0987614     0.37   0.709    -.1567735    .2303642
                                             1.p_rene |  -.2177771   .1119601    -1.95   0.052    -.4372149    .0016607
                                                _cons |   7.387266   .0647979   114.00   0.000     7.260265    7.514268
    -------------------------------------------------------------------------------------------------------

    ------------------------------------------------------------------------------
      Random-effects Parameters  |   Estimate   Std. Err.     [95% Conf. Interval]
    -----------------------------+------------------------------------------------
    progr: Unstructured          |
             var(b_day_malattia) |   .0011768   .0001991      .0008446    .0016397
```

```
             var(_cons) |   .1916417   .0284008      .1433323    .2562335
   cov(b_day_malattia,_cons) |   -.006868   .0020182     -.0108237   -.0029123
-----------------------------+------------------------------------------------
               var(Residual) |    .104534   .0042586      .0965119    .1132229
------------------------------------------------------------------------------
LR test vs. linear model: chi2(3) = 1160.20              Prob > chi2 = 0.0000

Note: LR test is conservative and provided only for reference.

103 . est store quadratic

104 . lrtest linear quadratic

Likelihood-ratio test                                 LR chi2(2)  =    130.98
(Assumption: linear nested in quadratic)              Prob > chi2 =    0.0000

105 . ****margins****
106 . quiet: mixed lym_ln  c.b_day_malattia##c.b_day_malattia##i.death_30 i.p_age2 i.p_cardio i.p_obeso i.p_rene || prog
    > r:b_day_malattia, cov(unstr)

107 . margins death_30, at(b_day_malattia=(0(1)21))  expression(exp(predict(xb))) asbal

Adjusted predictions                            Number of obs     =      1,805

Expression   : exp(predict(xb))

1._at        : b_day_mala~a    =           0
               death_30                      (asbalanced)
               p_age2                        (asbalanced)
               p_cardio                      (asbalanced)
               p_obeso                       (asbalanced)
               p_rene                        (asbalanced)

2._at        : b_day_mala~a    =           1
               death_30                      (asbalanced)
               p_age2                        (asbalanced)
               p_cardio                      (asbalanced)
               p_obeso                       (asbalanced)
               p_rene                        (asbalanced)

3._at        : b_day_mala~a    =           2
               death_30                      (asbalanced)
               p_age2                        (asbalanced)
               p_cardio                      (asbalanced)
               p_obeso                       (asbalanced)
               p_rene                        (asbalanced)

4._at        : b_day_mala~a    =           3
               death_30                      (asbalanced)
               p_age2                        (asbalanced)
               p_cardio                      (asbalanced)
               p_obeso                       (asbalanced)
               p_rene                        (asbalanced)

5._at        : b_day_mala~a    =           4
               death_30                      (asbalanced)
               p_age2                        (asbalanced)
               p_cardio                      (asbalanced)
               p_obeso                       (asbalanced)
               p_rene                        (asbalanced)

6._at        : b_day_mala~a    =           5
               death_30                      (asbalanced)
               p_age2                        (asbalanced)
               p_cardio                      (asbalanced)
               p_obeso                       (asbalanced)
               p_rene                        (asbalanced)

7._at        : b_day_mala~a    =           6
               death_30                      (asbalanced)
               p_age2                        (asbalanced)
               p_cardio                      (asbalanced)
               p_obeso                       (asbalanced)
               p_rene                        (asbalanced)

8._at        : b_day_mala~a    =           7
               death_30                      (asbalanced)
               p_age2                        (asbalanced)
               p_cardio                      (asbalanced)
               p_obeso                       (asbalanced)
               p_rene                        (asbalanced)

9._at        : b_day_mala~a    =           8
               death_30                      (asbalanced)
               p_age2                        (asbalanced)
               p_cardio                      (asbalanced)
               p_obeso                       (asbalanced)
               p_rene                        (asbalanced)

10._at       : b_day_mala~a    =           9
               death_30                      (asbalanced)
               p_age2                        (asbalanced)
               p_cardio                      (asbalanced)
               p_obeso                       (asbalanced)
               p_rene                        (asbalanced)

11._at       : b_day_mala~a    =          10
               death_30                      (asbalanced)
               p_age2                        (asbalanced)
               p_cardio                      (asbalanced)
               p_obeso                       (asbalanced)
               p_rene                        (asbalanced)

12._at       : b_day_mala~a    =          11
               death_30                      (asbalanced)
               p_age2                        (asbalanced)
               p_cardio                      (asbalanced)
               p_obeso                       (asbalanced)
```

```
              p_rene                        (asbalanced)

13._at        : b_day_mala~a    =          12
              death_30                      (asbalanced)
              p_age2                        (asbalanced)
              p_cardio                      (asbalanced)
              p_obeso                       (asbalanced)
              p_rene                        (asbalanced)

14._at        : b_day_mala~a    =          13
              death_30                      (asbalanced)
              p_age2                        (asbalanced)
              p_cardio                      (asbalanced)
              p_obeso                       (asbalanced)
              p_rene                        (asbalanced)

15._at        : b_day_mala~a    =          14
              death_30                      (asbalanced)
              p_age2                        (asbalanced)
              p_cardio                      (asbalanced)
              p_obeso                       (asbalanced)
              p_rene                        (asbalanced)

16._at        : b_day_mala~a    =          15
              death_30                      (asbalanced)
              p_age2                        (asbalanced)
              p_cardio                      (asbalanced)
              p_obeso                       (asbalanced)
              p_rene                        (asbalanced)

17._at        : b_day_mala~a    =          16
              death_30                      (asbalanced)
              p_age2                        (asbalanced)
              p_cardio                      (asbalanced)
              p_obeso                       (asbalanced)
              p_rene                        (asbalanced)

18._at        : b_day_mala~a    =          17
              death_30                      (asbalanced)
              p_age2                        (asbalanced)
              p_cardio                      (asbalanced)
              p_obeso                       (asbalanced)
              p_rene                        (asbalanced)

19._at        : b_day_mala~a    =          18
              death_30                      (asbalanced)
              p_age2                        (asbalanced)
              p_cardio                      (asbalanced)
              p_obeso                       (asbalanced)
              p_rene                        (asbalanced)

20._at        : b_day_mala~a    =          19
              death_30                      (asbalanced)
              p_age2                        (asbalanced)
              p_cardio                      (asbalanced)
              p_obeso                       (asbalanced)
              p_rene                        (asbalanced)

21._at        : b_day_mala~a    =          20
              death_30                      (asbalanced)
              p_age2                        (asbalanced)
              p_cardio                      (asbalanced)
              p_obeso                       (asbalanced)
              p_rene                        (asbalanced)

22._at        : b_day_mala~a    =          21
              death_30                      (asbalanced)
              p_age2                        (asbalanced)
              p_cardio                      (asbalanced)
              p_obeso                       (asbalanced)
              p_rene                        (asbalanced)
```

	Margin	Delta-method Std. Err.	z	P>|z|	[95% Conf.	Interval]
_at#death_30						
1#survivor	1327.064	121.8415	10.89	0.000	1088.259	1565.869
1#nonsurvivor	996.9008	134.1	7.43	0.000	734.0696	1259.732
2#survivor	1232.982	107.4015	11.48	0.000	1022.479	1443.485
2#nonsurvivor	900.7312	107.7332	8.36	0.000	689.578	1111.884
3#survivor	1154.48	96.18505	12.00	0.000	965.9605	1342.999
3#nonsurvivor	820.6163	88.17468	9.31	0.000	647.7971	993.4355
4#survivor	1089.383	87.54858	12.44	0.000	917.7906	1260.975
4#nonsurvivor	753.853	73.97559	10.19	0.000	608.8635	898.8425
5#survivor	1035.951	80.97907	12.79	0.000	877.2349	1194.667
5#nonsurvivor	698.2884	63.97407	10.92	0.000	572.9015	823.6753
6#survivor	992.8017	76.0687	13.05	0.000	843.7098	1141.894
6#nonsurvivor	652.2057	57.2013	11.40	0.000	540.0932	764.3182
7#survivor	958.8494	72.49614	13.23	0.000	816.7595	1100.939
7#nonsurvivor	614.2371	52.83886	11.62	0.000	510.6748	717.7993
8#survivor	933.2604	70.01245	13.33	0.000	796.0385	1070.482
8#nonsurvivor	583.2961	50.21647	11.62	0.000	484.8736	681.7186
9#survivor	915.4189	68.4298	13.38	0.000	781.299	1049.539
9#nonsurvivor	558.5264	48.82146	11.44	0.000	462.8381	654.2147
10#survivor	904.9019	67.61215	13.38	0.000	772.3845	1037.419
10#nonsurvivor	539.2623	48.29581	11.17	0.000	444.6042	633.9203
11#survivor	901.4625	67.46781	13.36	0.000	769.228	1033.697
11#nonsurvivor	524.9984	48.41708	10.84	0.000	430.1027	619.8941
12#survivor	905.0206	67.94383	13.32	0.000	771.8531	1038.188
12#nonsurvivor	515.3681	49.07239	10.50	0.000	419.188	611.5482
13#survivor	915.659	69.0224	13.27	0.000	780.3776	1050.94
13#nonsurvivor	510.1275	50.2349	10.15	0.000	411.6689	608.5861
14#survivor	933.6277	70.71949	13.20	0.000	795.02	1072.235
14#nonsurvivor	509.1451	51.94742	9.80	0.000	407.33	610.9602
15#survivor	959.3525	73.08598	13.13	0.000	816.1066	1102.598
15#nonsurvivor	512.3963	54.31339	9.43	0.000	405.944	618.8486
16#survivor	993.4529	76.21138	13.04	0.000	844.0813	1142.824

```
     16#nonsurvivor |   519.9626   57.49391     9.04   0.000     407.2766    632.6486
        17#survivor |   1036.766   80.23053    12.92   0.000     879.5175    1194.015
     17#nonsurvivor |   532.0345   61.70902     8.62   0.000     411.0871     652.982
        18#survivor |   1090.383   85.33365    12.78   0.000     923.1324    1257.634
     18#nonsurvivor |   548.9201   67.24221     8.16   0.000     417.1278    680.7124
        19#survivor |   1155.692    91.7802    12.59   0.000     975.8058    1335.578
     19#nonsurvivor |   571.0579   74.44802     7.67   0.000     425.1424    716.9733
        20#survivor |   1234.438    99.9172    12.35   0.000     1038.604    1430.272
     20#nonsurvivor |   599.0357   83.76392     7.15   0.000     434.8615      763.21
        21#survivor |   1328.805    110.203    12.06   0.000     1112.811    1544.799
     21#nonsurvivor |   633.6172   95.72881     6.62   0.000     445.9922    821.2422
        22#survivor |   1441.511   123.2375    11.70   0.000      1199.97    1683.052
     22#nonsurvivor |   675.7761   111.0108     6.09   0.000     458.1989    893.3532
--------------------------------------------------------------------------------

108 . marginsplot, ytitle("Cells per mmc") xlab(0(1)21) ylab(0(1000)3000) yline(200, lcolor(red)) title ("") legend(off)

      Variables that uniquely identify margins: b_day_malattia death_30

109 . margins ar.death_30, at(b_day_malattia=(0(1)21))  expression(exp(predict(xb))) asbal

    Contrasts of adjusted predictions               Number of obs     =      1,805

    Expression   : exp(predict(xb))

    1._at        : b_day_mala~a    =           0
                   death_30                      (asbalanced)
                   p_age2                        (asbalanced)
                   p_cardio                      (asbalanced)
                   p_obeso                       (asbalanced)
                   p_rene                        (asbalanced)

    2._at        : b_day_mala~a    =           1
                   death_30                      (asbalanced)
                   p_age2                        (asbalanced)
                   p_cardio                      (asbalanced)
                   p_obeso                       (asbalanced)
                   p_rene                        (asbalanced)

    3._at        : b_day_mala~a    =           2
                   death_30                      (asbalanced)
                   p_age2                        (asbalanced)
                   p_cardio                      (asbalanced)
                   p_obeso                       (asbalanced)
                   p_rene                        (asbalanced)

    4._at        : b_day_mala~a    =           3
                   death_30                      (asbalanced)
                   p_age2                        (asbalanced)
                   p_cardio                      (asbalanced)
                   p_obeso                       (asbalanced)
                   p_rene                        (asbalanced)

    5._at        : b_day_mala~a    =           4
                   death_30                      (asbalanced)
                   p_age2                        (asbalanced)
                   p_cardio                      (asbalanced)
                   p_obeso                       (asbalanced)
                   p_rene                        (asbalanced)

    6._at        : b_day_mala~a    =           5
                   death_30                      (asbalanced)
                   p_age2                        (asbalanced)
                   p_cardio                      (asbalanced)
                   p_obeso                       (asbalanced)
                   p_rene                        (asbalanced)

    7._at        : b_day_mala~a    =           6
                   death_30                      (asbalanced)
                   p_age2                        (asbalanced)
                   p_cardio                      (asbalanced)
                   p_obeso                       (asbalanced)
                   p_rene                        (asbalanced)

    8._at        : b_day_mala~a    =           7
                   death_30                      (asbalanced)
                   p_age2                        (asbalanced)
                   p_cardio                      (asbalanced)
                   p_obeso                       (asbalanced)
                   p_rene                        (asbalanced)

    9._at        : b_day_mala~a    =           8
                   death_30                      (asbalanced)
                   p_age2                        (asbalanced)
                   p_cardio                      (asbalanced)
                   p_obeso                       (asbalanced)
                   p_rene                        (asbalanced)

    10._at       : b_day_mala~a    =           9
                   death_30                      (asbalanced)
                   p_age2                        (asbalanced)
                   p_cardio                      (asbalanced)
                   p_obeso                       (asbalanced)
                   p_rene                        (asbalanced)

    11._at       : b_day_mala~a    =          10
                   death_30                      (asbalanced)
                   p_age2                        (asbalanced)
                   p_cardio                      (asbalanced)
                   p_obeso                       (asbalanced)
                   p_rene                        (asbalanced)

    12._at       : b_day_mala~a    =          11
                   death_30                      (asbalanced)
                   p_age2                        (asbalanced)
                   p_cardio                      (asbalanced)
                   p_obeso                       (asbalanced)
                   p_rene                        (asbalanced)
```

```
13._at       : b_day_mala~a    =          12
               death_30                      (asbalanced)
               p_age2                        (asbalanced)
               p_cardio                      (asbalanced)
               p_obeso                       (asbalanced)
               p_rene                        (asbalanced)

14._at       : b_day_mala~a    =          13
               death_30                      (asbalanced)
               p_age2                        (asbalanced)
               p_cardio                      (asbalanced)
               p_obeso                       (asbalanced)
               p_rene                        (asbalanced)

15._at       : b_day_mala~a    =          14
               death_30                      (asbalanced)
               p_age2                        (asbalanced)
               p_cardio                      (asbalanced)
               p_obeso                       (asbalanced)
               p_rene                        (asbalanced)

16._at       : b_day_mala~a    =          15
               death_30                      (asbalanced)
               p_age2                        (asbalanced)
               p_cardio                      (asbalanced)
               p_obeso                       (asbalanced)
               p_rene                        (asbalanced)

17._at       : b_day_mala~a    =          16
               death_30                      (asbalanced)
               p_age2                        (asbalanced)
               p_cardio                      (asbalanced)
               p_obeso                       (asbalanced)
               p_rene                        (asbalanced)

18._at       : b_day_mala~a    =          17
               death_30                      (asbalanced)
               p_age2                        (asbalanced)
               p_cardio                      (asbalanced)
               p_obeso                       (asbalanced)
               p_rene                        (asbalanced)

19._at       : b_day_mala~a    =          18
               death_30                      (asbalanced)
               p_age2                        (asbalanced)
               p_cardio                      (asbalanced)
               p_obeso                       (asbalanced)
               p_rene                        (asbalanced)

20._at       : b_day_mala~a    =          19
               death_30                      (asbalanced)
               p_age2                        (asbalanced)
               p_cardio                      (asbalanced)
               p_obeso                       (asbalanced)
               p_rene                        (asbalanced)

21._at       : b_day_mala~a    =          20
               death_30                      (asbalanced)
               p_age2                        (asbalanced)
               p_cardio                      (asbalanced)
               p_obeso                       (asbalanced)
               p_rene                        (asbalanced)

22._at       : b_day_mala~a    =          21
               death_30                      (asbalanced)
               p_age2                        (asbalanced)
               p_cardio                      (asbalanced)
               p_obeso                       (asbalanced)
               p_rene                        (asbalanced)
```

	df	chi2	P>chi2
death_30@_at			
(nonsurvivor vs survivor) 1	1	4.55	0.0329
(nonsurvivor vs survivor) 2	1	7.02	0.0081
(nonsurvivor vs survivor) 3	1	10.38	0.0013
(nonsurvivor vs survivor) 4	1	14.61	0.0001
(nonsurvivor vs survivor) 5	1	19.37	0.0000
(nonsurvivor vs survivor) 6	1	24.13	0.0000
(nonsurvivor vs survivor) 7	1	28.33	0.0000
(nonsurvivor vs survivor) 8	1	31.67	0.0000
(nonsurvivor vs survivor) 9	1	34.12	0.0000
(nonsurvivor vs survivor) 10	1	35.88	0.0000
(nonsurvivor vs survivor) 11	1	37.13	0.0000
(nonsurvivor vs survivor) 12	1	38.04	0.0000
(nonsurvivor vs survivor) 13	1	38.70	0.0000
(nonsurvivor vs survivor) 14	1	39.11	0.0000
(nonsurvivor vs survivor) 15	1	39.25	0.0000
(nonsurvivor vs survivor) 16	1	39.03	0.0000
(nonsurvivor vs survivor) 17	1	38.36	0.0000
(nonsurvivor vs survivor) 18	1	37.19	0.0000
(nonsurvivor vs survivor) 19	1	35.49	0.0000
(nonsurvivor vs survivor) 20	1	33.29	0.0000
(nonsurvivor vs survivor) 21	1	30.70	0.0000
(nonsurvivor vs survivor) 22	1	27.87	0.0000
Joint	5	40.30	0.0000

	Contrast	Delta-method Std. Err.	[95% Conf.	Interval]
death_30@_at				
(nonsurvivor vs survivor) 1	-330.1628	154.7546	-633.4764	-26.84931
(nonsurvivor vs survivor) 2	-332.2508	125.4147	-578.0591	-86.4425
(nonsurvivor vs survivor) 3	-333.8635	103.6061	-536.9277	-130.7993

```
    (nonsurvivor vs survivor)  4  |  -335.5297   87.78817     -507.5914   -163.4681
    (nonsurvivor vs survivor)  5  |  -337.6626   76.71533     -488.0219   -187.3033
    (nonsurvivor vs survivor)  6  |   -340.596   69.33495       -476.49   -204.7019
    (nonsurvivor vs survivor)  7  |  -344.6123   64.74366     -471.5075    -217.717
    (nonsurvivor vs survivor)  8  |  -349.9643   62.19066     -471.8558   -228.0729
    (nonsurvivor vs survivor)  9  |  -356.8925   61.09448     -476.6354   -237.1495
    (nonsurvivor vs survivor) 10  |  -365.6396    61.0446     -485.2848   -245.9944
    (nonsurvivor vs survivor) 11  |  -376.4641   61.78297     -497.5565   -255.3717
    (nonsurvivor vs survivor) 12  |  -389.6524   63.17631     -513.4757   -265.8292
    (nonsurvivor vs survivor) 13  |  -405.5315   65.19049     -533.3026   -277.7605
    (nonsurvivor vs survivor) 14  |  -424.4826   67.87286     -557.5109   -291.4542
    (nonsurvivor vs survivor) 15  |  -446.9562   71.34326     -586.7864   -307.1259
    (nonsurvivor vs survivor) 16  |  -473.4903   75.79296     -622.0418   -324.9388
    (nonsurvivor vs survivor) 17  |  -504.7319   81.48962     -664.4487   -345.0152
    (nonsurvivor vs survivor) 18  |  -541.4631   88.78721     -715.4829   -367.4434
    (nonsurvivor vs survivor) 19  |  -584.6338   98.14034     -776.9853   -392.2822
    (nonsurvivor vs survivor) 20  |  -635.4025   110.1238     -851.2412   -419.5638
    (nonsurvivor vs survivor) 21  |   -695.188   125.4596     -941.0843   -449.2917
    (nonsurvivor vs survivor) 22  |  -765.7346   145.0545     -1050.036    -481.433
    ------------------------------------------------------------------------------

110 . graph save "Graph" "/Users/Chiara/Documents/fileDO 15 Maggio/Marg_LYM.gph", replace
    (file /Users/Chiara/Documents/fileDO 15 Maggio/Marg_LYM.gph saved)

111 .
112 . *margins death_30, at(b_day_malattia=(0(1)30))  expression(exp(predict(xb)))
113 . *marginsplot, title("Lymphocites kinetic day 0 to day 30 after onset") ytitle("Cells per mmc (log-scale)") xlab(0(
    > 5)30) ylab(0(250)2500, angle(horizontal)) yline(800, lcolor(green)) yline(500, lcolor(orange)) yline(200, lcolor(r
    > ed)) legend(off)
114 . *margins ar.death_30, at(b_day_malattia=(0(1)30))  expression(exp(predict(xb)))
115 .
116 . ***combine*****
117 . gr combine "/Users/Chiara/Documents/fileDO 15 Maggio/Marg_LYM.gph" "/Users/Chiara/Documents/fileDO 15 Maggio/BOX_L
    > YM.gph", ycomm   xsize(8) ysize(4)

118 . graph save "Graph" "/Users/Chiara/Documents/fileDO 15 Maggio/Graph_LYM.gph", replace
    (file /Users/Chiara/Documents/fileDO 15 Maggio/Graph_LYM.gph saved)

119 .
120 .
121 . ****************************************************************************
122 . *** analisi Monocytes  ***************************************************
123 . ****************************************************************************
124 . **** scatter no model
125 . use "/Users/Chiara/Documents/fileDO 15 Maggio/fileDO\coorte_long_emocromo_anemia_1.dta", clear

126 . *scatter mono b_day_malattia  if death_30==1, mcolor(red) legend(off) title("Monocites variation over time") xtitl
    > e("day since onset") ytitle("Cells per mmc X 1000") xlab(0(5)30)|| scatter mono b_day_malattia  if death_30==0, mc
    > olor(green) legend(off) ms(oh)
127 .
128 . *********Graph box**************************************
129 . replace mono=mono*1000
    (1,805 real changes made)

130 . graph box mono, over(death_30, label(nolabel)) over(b_day_malattia) asyvars box(1, fcolor(navy))  nooutsides ytitl
    > e(Cells per mmc) yline(100, lcolor(red)) legend(off)

131 . graph save "Graph" "/Users/Chiara/Documents/fileDO 15 Maggio/BOX_MONO.gph", replace
    (file /Users/Chiara/Documents/fileDO 15 Maggio/BOX_MONO.gph saved)

132 .
133 . **** modelling
134 . use "/Users/Chiara/Documents/fileDO 15 Maggio/fileDO\coorte_long_emocromo_anemia_1.dta", clear

135 . mixed mono_ln c.b_day_malattia##i.death_30 || progr:b_day_malattia, cov(unstr)

    Performing EM optimization:

    Performing gradient-based optimization:

    Iteration 0:   log likelihood = -1234.8149
    Iteration 1:   log likelihood =  -1234.808
    Iteration 2:   log likelihood =  -1234.808

    Computing standard errors:

    Mixed-effects ML regression                     Number of obs     =      1,805
    Group variable: progr                           Number of groups  =        379

                                                    Obs per group:
                                                                  min =          1
                                                                  avg =        4.8
                                                                  max =         22

                                                    Wald chi2(3)      =     131.44
    Log likelihood =  -1234.808                     Prob > chi2       =     0.0000

    -------------------------------------------------------------------------------------------
                   mono_ln |      Coef.   Std. Err.      z    P>|z|     [95% Conf. Interval]
    -----------------------+-------------------------------------------------------------------
            b_day_malattia |   .0338711    .003426     9.89   0.000     .0271563     .040586
                           |
                  death_30 |
               nonsurvivor |  -.2782923   .1340952    -2.08   0.038    -.5411141   -.0154705
                           |
    death_30#c.b_day_malattia |
               nonsurvivor |  -.0015475   .0097598    -0.16   0.874    -.0206764    .0175815
                           |
                     _cons |   5.782126   .0502506   115.07   0.000     5.683637    5.880616
    -------------------------------------------------------------------------------------------

    ------------------------------------------------------------------------------
      Random-effects Parameters  |   Estimate   Std. Err.     [95% Conf. Interval]
    -----------------------------+------------------------------------------------
    progr: Unstructured          |
           var(b_day_malattia)   |   .0014032   .0002267      .0010224    .0019259
                     var(_cons)  |   .4201148   .0537424      .3269487    .5398291
      cov(b_day_malattia,_cons)  |  -.0196816   .0032793     -.0261089   -.0132542
```

```
                 var(Residual)    .1478748    .005912      .1367298    .1599283

LR test vs. linear model: chi2(3) = 695.19               Prob > chi2 = 0.0000

Note: LR test is conservative and provided only for reference.

136 . est store linear

137 . mixed mono_ln  c.b_day_malattia##c.b_day_malattia##i.death_30 i.p_age2 i.p_cardio i.p_obeso i.p_rene|| progr:b_day
    > _malattia, cov(unstr)

Performing EM optimization:

Performing gradient-based optimization:

Iteration 0:   log likelihood = -1214.9499
Iteration 1:   log likelihood = -1214.9415
Iteration 2:   log likelihood = -1214.9415

Computing standard errors:

Mixed-effects ML regression                     Number of obs     =      1,805
Group variable: progr                           Number of groups  =        379

                                                Obs per group:
                                                              min =          1
                                                              avg =        4.8
                                                              max =         22

                                                Wald chi2(9)      =     181.99
Log likelihood = -1214.9415                     Prob > chi2       =     0.0000

--------------------------------------------------------------------------------------------------------------
                                 mono_ln |      Coef.   Std. Err.      z    P>|z|     [95% Conf. Interval]
-----------------------------------------+--------------------------------------------------------------------
                           b_day_malattia |  -.0175094   .0108672    -1.61   0.107    -.0388086    .0037899
                                          |
          c.b_day_malattia#c.b_day_malattia |   .0021106   .0004208     5.02   0.000     .0012857    .0029354
                                          |
                                 death_30 |
                              nonsurvivor  |  -.1891192   .1752773    -1.08   0.281    -.5326564    .1544181
                                          |
                 death_30#c.b_day_malattia |
                              nonsurvivor  |   -.016233   .0275329    -0.59   0.555    -.0701965    .0377305
                                          |
death_30#c.b_day_malattia#c.b_day_malattia |
                              nonsurvivor  |   .0008249   .0011461     0.72   0.472    -.0014214    .0030712
                                          |
                                 1.p_age2 |  -.1042496   .0501716    -2.08   0.038    -.2025841    -.005915
                               1.p_cardio |  -.0082546   .0533906    -0.15   0.877    -.1128982     .096389
                                1.p_obeso |  -.0116325   .0938326    -0.12   0.901     -.195541    .1722761
                                 1.p_rene |  -.1855588   .1085617    -1.71   0.087    -.3983357    .0272182
                                    _cons |    6.11702     .07747    78.96   0.000     5.965181    6.268858
--------------------------------------------------------------------------------------------------------------

------------------------------------------------------------------------------
  Random-effects Parameters  |   Estimate   Std. Err.     [95% Conf. Interval]
-----------------------------+------------------------------------------------
progr: Unstructured          |
         var(b_day_malattia) |   .0012124   .0002085      .0008654    .0016984
                   var(_cons) |   .3662504   .0493641      .2812233    .4769853
    cov(b_day_malattia,_cons) |  -.0165163   .0030029     -.0224018   -.0106308
-----------------------------+------------------------------------------------
                var(Residual) |   .1466274   .0058517      .1355953     .158557
------------------------------------------------------------------------------
LR test vs. linear model: chi2(3) = 691.78               Prob > chi2 = 0.0000

Note: LR test is conservative and provided only for reference.

138 . est store quadratic

139 . lrtest linear quadratic

Likelihood-ratio test                                 LR chi2(6)  =     39.73
(Assumption: linear nested in quadratic)              Prob > chi2 =    0.0000

140 . ****margins*****
141 . quiet: mixed mono_ln  c.b_day_malattia##c.b_day_malattia##i.death_30 i.p_age2 i.p_cardio i.p_obeso i.p_rene|| prog
    > r:b_day_malattia, cov(unstr)

142 . margins death_30, at(b_day_malattia=(0(1)21))  expression(exp(predict(xb))) asbal

Adjusted predictions                            Number of obs     =      1,805

Expression   : exp(predict(xb))

1._at        : b_day_mala~a    =           0
               death_30                      (asbalanced)
               p_age2                        (asbalanced)
               p_cardio                      (asbalanced)
               p_obeso                       (asbalanced)
               p_rene                        (asbalanced)

2._at        : b_day_mala~a    =           1
               death_30                      (asbalanced)
               p_age2                        (asbalanced)
               p_cardio                      (asbalanced)
               p_obeso                       (asbalanced)
               p_rene                        (asbalanced)

3._at        : b_day_mala~a    =           2
               death_30                      (asbalanced)
               p_age2                        (asbalanced)
               p_cardio                      (asbalanced)
               p_obeso                       (asbalanced)
               p_rene                        (asbalanced)
```

```
4._at        : b_day_mala~a    =           3
               death_30                     (asbalanced)
               p_age2                       (asbalanced)
               p_cardio                     (asbalanced)
               p_obeso                      (asbalanced)
               p_rene                       (asbalanced)

5._at        : b_day_mala~a    =           4
               death_30                     (asbalanced)
               p_age2                       (asbalanced)
               p_cardio                     (asbalanced)
               p_obeso                      (asbalanced)
               p_rene                       (asbalanced)

6._at        : b_day_mala~a    =           5
               death_30                     (asbalanced)
               p_age2                       (asbalanced)
               p_cardio                     (asbalanced)
               p_obeso                      (asbalanced)
               p_rene                       (asbalanced)

7._at        : b_day_mala~a    =           6
               death_30                     (asbalanced)
               p_age2                       (asbalanced)
               p_cardio                     (asbalanced)
               p_obeso                      (asbalanced)
               p_rene                       (asbalanced)

8._at        : b_day_mala~a    =           7
               death_30                     (asbalanced)
               p_age2                       (asbalanced)
               p_cardio                     (asbalanced)
               p_obeso                      (asbalanced)
               p_rene                       (asbalanced)

9._at        : b_day_mala~a    =           8
               death_30                     (asbalanced)
               p_age2                       (asbalanced)
               p_cardio                     (asbalanced)
               p_obeso                      (asbalanced)
               p_rene                       (asbalanced)

10._at       : b_day_mala~a    =           9
               death_30                     (asbalanced)
               p_age2                       (asbalanced)
               p_cardio                     (asbalanced)
               p_obeso                      (asbalanced)
               p_rene                       (asbalanced)

11._at       : b_day_mala~a    =          10
               death_30                     (asbalanced)
               p_age2                       (asbalanced)
               p_cardio                     (asbalanced)
               p_obeso                      (asbalanced)
               p_rene                       (asbalanced)

12._at       : b_day_mala~a    =          11
               death_30                     (asbalanced)
               p_age2                       (asbalanced)
               p_cardio                     (asbalanced)
               p_obeso                      (asbalanced)
               p_rene                       (asbalanced)

13._at       : b_day_mala~a    =          12
               death_30                     (asbalanced)
               p_age2                       (asbalanced)
               p_cardio                     (asbalanced)
               p_obeso                      (asbalanced)
               p_rene                       (asbalanced)

14._at       : b_day_mala~a    =          13
               death_30                     (asbalanced)
               p_age2                       (asbalanced)
               p_cardio                     (asbalanced)
               p_obeso                      (asbalanced)
               p_rene                       (asbalanced)

15._at       : b_day_mala~a    =          14
               death_30                     (asbalanced)
               p_age2                       (asbalanced)
               p_cardio                     (asbalanced)
               p_obeso                      (asbalanced)
               p_rene                       (asbalanced)

16._at       : b_day_mala~a    =          15
               death_30                     (asbalanced)
               p_age2                       (asbalanced)
               p_cardio                     (asbalanced)
               p_obeso                      (asbalanced)
               p_rene                       (asbalanced)

17._at       : b_day_mala~a    =          16
               death_30                     (asbalanced)
               p_age2                       (asbalanced)
               p_cardio                     (asbalanced)
               p_obeso                      (asbalanced)
               p_rene                       (asbalanced)

18._at       : b_day_mala~a    =          17
               death_30                     (asbalanced)
               p_age2                       (asbalanced)
               p_cardio                     (asbalanced)
               p_obeso                      (asbalanced)
               p_rene                       (asbalanced)

19._at       : b_day_mala~a    =          18
               death_30                     (asbalanced)
               p_age2                       (asbalanced)
```

```
              p_cardio                      (asbalanced)
              p_obeso                       (asbalanced)
              p_rene                        (asbalanced)

20._at      : b_day_mala~a    =          19
              death_30                      (asbalanced)
              p_age2                        (asbalanced)
              p_cardio                      (asbalanced)
              p_obeso                       (asbalanced)
              p_rene                        (asbalanced)

21._at      : b_day_mala~a    =          20
              death_30                      (asbalanced)
              p_age2                        (asbalanced)
              p_cardio                      (asbalanced)
              p_obeso                       (asbalanced)
              p_rene                        (asbalanced)

22._at      : b_day_mala~a    =          21
              death_30                      (asbalanced)
              p_age2                        (asbalanced)
              p_cardio                      (asbalanced)
              p_obeso                       (asbalanced)
              p_rene                        (asbalanced)
```

	Margin	Delta-method Std. Err.	z	P>|z|	[95% Conf.	Interval]
_at#death_30						
1#survivor	388.4529	38.65304	10.05	0.000	312.6944	464.2115
1#nonsurvivor	321.5178	52.81145	6.09	0.000	218.0092	425.0263
2#survivor	382.517	35.56431	10.76	0.000	312.8123	452.2218
2#nonsurvivor	311.7638	45.42954	6.86	0.000	222.7236	400.8041
3#survivor	378.2652	33.1177	11.42	0.000	313.3557	443.1747
3#nonsurvivor	304.0858	39.55271	7.69	0.000	226.5639	381.6077
4#survivor	375.6429	31.24492	12.02	0.000	314.404	436.8818
4#nonsurvivor	298.3433	35.01192	8.52	0.000	229.7212	366.9655
5#survivor	374.6168	29.88053	12.54	0.000	316.052	433.1815
5#nonsurvivor	294.4328	31.65583	9.30	0.000	232.3886	356.4771
6#survivor	375.1737	28.96117	12.95	0.000	318.4109	431.9366
6#nonsurvivor	292.2845	29.33417	9.96	0.000	234.7906	349.7785
7#survivor	377.3209	28.42626	13.27	0.000	321.6064	433.0353
7#nonsurvivor	291.8604	27.88858	10.47	0.000	237.1998	346.521
8#survivor	381.0855	28.21969	13.50	0.000	325.776	436.3951
8#nonsurvivor	293.1529	27.15522	10.80	0.000	239.9297	346.3762
9#survivor	386.5159	28.29181	13.66	0.000	331.0649	441.9668
9#nonsurvivor	296.1849	26.97756	10.98	0.000	243.3099	349.06
10#survivor	393.6818	28.60128	13.76	0.000	337.6243	449.7393
10#nonsurvivor	301.0104	27.22211	11.06	0.000	247.656	354.3647
11#survivor	402.6768	29.11654	13.83	0.000	345.6095	459.7442
11#nonsurvivor	307.7157	27.79056	11.07	0.000	253.2472	362.1842
12#survivor	413.6196	29.81685	13.87	0.000	355.1796	472.0595
12#nonsurvivor	316.4226	28.62718	11.05	0.000	260.3144	372.5309
13#survivor	426.6569	30.69338	13.90	0.000	366.499	486.8148
13#nonsurvivor	327.2918	29.72381	11.01	0.000	269.0342	385.5494
14#survivor	441.9668	31.75056	13.92	0.000	379.7369	504.1968
14#nonsurvivor	340.5277	31.1259	10.94	0.000	279.5221	401.5334
15#survivor	459.7627	33.00811	13.93	0.000	395.068	524.4574
15#nonsurvivor	356.3851	32.94153	10.82	0.000	291.8209	420.9493
16#survivor	480.2983	34.50394	13.92	0.000	412.6718	547.9248
16#nonsurvivor	375.1771	35.35299	10.61	0.000	305.8865	444.4677
17#survivor	503.8736	36.29824	13.88	0.000	432.7303	575.0168
17#nonsurvivor	397.2855	38.6281	10.28	0.000	321.5759	472.9952
18#survivor	530.842	38.4786	13.80	0.000	455.4253	606.2587
18#nonsurvivor	423.174	43.12683	9.81	0.000	338.647	507.701
19#survivor	561.6196	41.16598	13.64	0.000	480.9357	642.3034
19#nonsurvivor	453.4035	49.30136	9.20	0.000	356.7746	550.0324
20#survivor	596.6949	44.52116	13.40	0.000	509.4351	683.9548
20#nonsurvivor	488.6529	57.69343	8.47	0.000	375.5759	601.73
21#survivor	636.6426	48.75099	13.06	0.000	541.0924	732.1928
21#nonsurvivor	529.7438	68.93937	7.68	0.000	394.6251	664.8624
22#survivor	682.138	54.11422	12.61	0.000	576.0761	788.1999
22#nonsurvivor	577.6715	83.79256	6.89	0.000	413.4411	741.9019

```
143 . marginsplot, ytitle("Cells per mmc") xlab(0(1)21) ylab(0(500)2000) yline(100,lcolor(red)) legend(off)

      Variables that uniquely identify margins: b_day_malattia death_30

144 . margins ar.death_30, at(b_day_malattia=(0(1)21))  expression(exp(predict(xb))) asbal

    Contrasts of adjusted predictions               Number of obs     =      1,805

    Expression   : exp(predict(xb))

    1._at        : b_day_mala~a    =          0
                   death_30                      (asbalanced)
                   p_age2                        (asbalanced)
                   p_cardio                      (asbalanced)
                   p_obeso                       (asbalanced)
                   p_rene                        (asbalanced)

    2._at        : b_day_mala~a    =          1
                   death_30                      (asbalanced)
                   p_age2                        (asbalanced)
                   p_cardio                      (asbalanced)
                   p_obeso                       (asbalanced)
                   p_rene                        (asbalanced)

    3._at        : b_day_mala~a    =          2
                   death_30                      (asbalanced)
                   p_age2                        (asbalanced)
                   p_cardio                      (asbalanced)
                   p_obeso                       (asbalanced)
                   p_rene                        (asbalanced)

    4._at        : b_day_mala~a    =          3
```

```
                death_30                      (asbalanced)
                p_age2                        (asbalanced)
                p_cardio                      (asbalanced)
                p_obeso                       (asbalanced)
                p_rene                        (asbalanced)

5._at         : b_day_mala~a    =          4
                death_30                      (asbalanced)
                p_age2                        (asbalanced)
                p_cardio                      (asbalanced)
                p_obeso                       (asbalanced)
                p_rene                        (asbalanced)

6._at         : b_day_mala~a    =          5
                death_30                      (asbalanced)
                p_age2                        (asbalanced)
                p_cardio                      (asbalanced)
                p_obeso                       (asbalanced)
                p_rene                        (asbalanced)

7._at         : b_day_mala~a    =          6
                death_30                      (asbalanced)
                p_age2                        (asbalanced)
                p_cardio                      (asbalanced)
                p_obeso                       (asbalanced)
                p_rene                        (asbalanced)

8._at         : b_day_mala~a    =          7
                death_30                      (asbalanced)
                p_age2                        (asbalanced)
                p_cardio                      (asbalanced)
                p_obeso                       (asbalanced)
                p_rene                        (asbalanced)

9._at         : b_day_mala~a    =          8
                death_30                      (asbalanced)
                p_age2                        (asbalanced)
                p_cardio                      (asbalanced)
                p_obeso                       (asbalanced)
                p_rene                        (asbalanced)

10._at        : b_day_mala~a    =          9
                death_30                      (asbalanced)
                p_age2                        (asbalanced)
                p_cardio                      (asbalanced)
                p_obeso                       (asbalanced)
                p_rene                        (asbalanced)

11._at        : b_day_mala~a    =         10
                death_30                      (asbalanced)
                p_age2                        (asbalanced)
                p_cardio                      (asbalanced)
                p_obeso                       (asbalanced)
                p_rene                        (asbalanced)

12._at        : b_day_mala~a    =         11
                death_30                      (asbalanced)
                p_age2                        (asbalanced)
                p_cardio                      (asbalanced)
                p_obeso                       (asbalanced)
                p_rene                        (asbalanced)

13._at        : b_day_mala~a    =         12
                death_30                      (asbalanced)
                p_age2                        (asbalanced)
                p_cardio                      (asbalanced)
                p_obeso                       (asbalanced)
                p_rene                        (asbalanced)

14._at        : b_day_mala~a    =         13
                death_30                      (asbalanced)
                p_age2                        (asbalanced)
                p_cardio                      (asbalanced)
                p_obeso                       (asbalanced)
                p_rene                        (asbalanced)

15._at        : b_day_mala~a    =         14
                death_30                      (asbalanced)
                p_age2                        (asbalanced)
                p_cardio                      (asbalanced)
                p_obeso                       (asbalanced)
                p_rene                        (asbalanced)

16._at        : b_day_mala~a    =         15
                death_30                      (asbalanced)
                p_age2                        (asbalanced)
                p_cardio                      (asbalanced)
                p_obeso                       (asbalanced)
                p_rene                        (asbalanced)

17._at        : b_day_mala~a    =         16
                death_30                      (asbalanced)
                p_age2                        (asbalanced)
                p_cardio                      (asbalanced)
                p_obeso                       (asbalanced)
                p_rene                        (asbalanced)

18._at        : b_day_mala~a    =         17
                death_30                      (asbalanced)
                p_age2                        (asbalanced)
                p_cardio                      (asbalanced)
                p_obeso                       (asbalanced)
                p_rene                        (asbalanced)

19._at        : b_day_mala~a    =         18
                death_30                      (asbalanced)
                p_age2                        (asbalanced)
                p_cardio                      (asbalanced)
```

```
               p_obeso                        (asbalanced)
               p_rene                         (asbalanced)

20._at       : b_day_mala~a    =          19
               death_30                       (asbalanced)
               p_age2                         (asbalanced)
               p_cardio                       (asbalanced)
               p_obeso                        (asbalanced)
               p_rene                         (asbalanced)

21._at       : b_day_mala~a    =          20
               death_30                       (asbalanced)
               p_age2                         (asbalanced)
               p_cardio                       (asbalanced)
               p_obeso                        (asbalanced)
               p_rene                         (asbalanced)

22._at       : b_day_mala~a    =          21
               death_30                       (asbalanced)
               p_age2                         (asbalanced)
               p_cardio                       (asbalanced)
               p_obeso                        (asbalanced)
               p_rene                         (asbalanced)
```

	df	chi2	P>chi2
death_30@_at			
(nonsurvivor vs survivor) 1	1	1.28	0.2588
(nonsurvivor vs survivor) 2	1	1.93	0.1651
(nonsurvivor vs survivor) 3	1	2.81	0.0938
(nonsurvivor vs survivor) 4	1	3.92	0.0476
(nonsurvivor vs survivor) 5	1	5.23	0.0222
(nonsurvivor vs survivor) 6	1	6.60	0.0102
(nonsurvivor vs survivor) 7	1	7.88	0.0050
(nonsurvivor vs survivor) 8	1	8.91	0.0028
(nonsurvivor vs survivor) 9	1	9.61	0.0019
(nonsurvivor vs survivor) 10	1	10.00	0.0016
(nonsurvivor vs survivor) 11	1	10.13	0.0015
(nonsurvivor vs survivor) 12	1	10.05	0.0015
(nonsurvivor vs survivor) 13	1	9.79	0.0018
(nonsurvivor vs survivor) 14	1	9.36	0.0022
(nonsurvivor vs survivor) 15	1	8.75	0.0031
(nonsurvivor vs survivor) 16	1	7.92	0.0049
(nonsurvivor vs survivor) 17	1	6.88	0.0087
(nonsurvivor vs survivor) 18	1	5.67	0.0173
(nonsurvivor vs survivor) 19	1	4.40	0.0359
(nonsurvivor vs survivor) 20	1	3.21	0.0732
(nonsurvivor vs survivor) 21	1	2.20	0.1381
(nonsurvivor vs survivor) 22	1	1.42	0.2338
Joint	5	10.70	0.0577

	Contrast	Delta-method Std. Err.	[95% Conf.	Interval]
death_30@_at				
(nonsurvivor vs survivor) 1	-66.93515	59.27759	-183.1171	49.2468
(nonsurvivor vs survivor) 2	-70.7532	50.97133	-170.6552	29.14877
(nonsurvivor vs survivor) 3	-74.17936	44.27377	-160.9543	12.59563
(nonsurvivor vs survivor) 4	-77.29955	39.01844	-153.7743	-.8248194
(nonsurvivor vs survivor) 5	-80.18391	35.06014	-148.9005	-11.4673
(nonsurvivor vs survivor) 6	-82.88918	32.25529	-146.1084	-19.66996
(nonsurvivor vs survivor) 7	-85.46048	30.44761	-145.1367	-25.78426
(nonsurvivor vs survivor) 8	-87.93264	29.46608	-145.6851	-30.18017
(nonsurvivor vs survivor) 9	-90.33093	29.13683	-147.4381	-33.2238
(nonsurvivor vs survivor) 10	-92.67148	29.30219	-150.1027	-35.24024
(nonsurvivor vs survivor) 11	-94.96115	29.83817	-153.4429	-36.47941
(nonsurvivor vs survivor) 12	-97.19698	30.66603	-157.3013	-37.09267
(nonsurvivor vs survivor) 13	-99.3651	31.75998	-161.6135	-37.11669
(nonsurvivor vs survivor) 14	-101.4391	33.1549	-166.4215	-36.4567
(nonsurvivor vs survivor) 15	-103.3776	34.95704	-171.8922	-34.8631
(nonsurvivor vs survivor) 16	-105.1212	37.35783	-178.3412	-31.90126
(nonsurvivor vs survivor) 17	-106.588	40.64727	-186.2552	-26.92084
(nonsurvivor vs survivor) 18	-107.668	45.22107	-196.2997	-19.03637
(nonsurvivor vs survivor) 19	-108.216	51.57736	-209.3058	-7.126278
(nonsurvivor vs survivor) 20	-108.042	60.30757	-226.2427	10.15865
(nonsurvivor vs survivor) 21	-106.8988	72.095	-248.2024	34.40476
(nonsurvivor vs survivor) 22	-104.4665	87.73475	-276.4235	67.49041

```
145 . graph save "Graph" "/Users/Chiara/Documents/fileDO 15 Maggio/Marg_MONO.gph", replace
    (file /Users/Chiara/Documents/fileDO 15 Maggio/Marg_MONO.gph saved)

146 .
147 . *margins death_30, at(b_day_malattia=(0(1)30))  expression(exp(predict(xb)))
148 . *marginsplot, title("Monocites kinetic day 0 to day 30 after onset") ytitle("Cells per mmc (log-scale)") xlab(0(5)
    > 30) ylab(0(100)800) yline(100,lcolor(red)) legend(off)
149 . *margins ar.death_30, at(b_day_malattia=(0(1)30))  expression(exp(predict(xb)))
150 .
151 . ***combine*****
152 . gr combine "/Users/Chiara/Documents/fileDO 15 Maggio/Marg_MONO.gph" "/Users/Chiara/Documents/fileDO 15 Maggio/BOX_
    > MONO.gph", ycomm   xsize(8) ysize(4)

153 . graph save "Graph" "/Users/Chiara/Documents/fileDO 15 Maggio/Graph_MONO.gph", replace
    (file /Users/Chiara/Documents/fileDO 15 Maggio/Graph_MONO.gph saved)

154 .
155 .
156 . ***************************************************************************
157 . *** analisi Eosinophils (senza trasfgomrazione ci sono tanti ZERI ********
158 . ***************************************************************************
159 . **** scatter no model
160 . use "/Users/Chiara/Documents/fileDO 15 Maggio/fileDO\coorte_long_emocromo_anemia_1.dta", clear

161 . scatter eos b_day_malattia  if death_30==1, mcolor(red) legend(off) title("Eosinophils variation over time") xtitl
    > e("day since onset") ytitle("Cells per mmc X 1000") || scatter eos b_day_malattia  if death_30==0, mcolor(green) l
```

```
    > egend(off) ms(oh)

162 .
163 . **** modelling
164 . use "/Users/Chiara/Documents/fileDO 15 Maggio/fileDO\coorte_long_emocromo_anemia_1.dta", clear

165 . replace eos=eos*1000
    (961 real changes made)

166 . mixed eos c.b_day_malattia##i.death_30 || progr:b_day_malattia, cov(unstr)

    Performing EM optimization:

    Performing gradient-based optimization:

    Iteration 0:   log likelihood = -10062.085
    Iteration 1:   log likelihood = -10062.036
    Iteration 2:   log likelihood = -10062.036

    Computing standard errors:

    Mixed-effects ML regression                     Number of obs     =      1,805
    Group variable: progr                           Number of groups  =        379

                                                    Obs per group:
                                                                  min =          1
                                                                  avg =        4.8
                                                                  max =         22

                                                    Wald chi2(3)      =     121.64
    Log likelihood = -10062.036                     Prob > chi2       =     0.0000

    --------------------------------------------------------------------------------------------
                             eos |      Coef.   Std. Err.      z    P>|z|     [95% Conf. Interval]
    -----------------------------+--------------------------------------------------------------
                    b_day_malattia |   5.120101   .4880917    10.49   0.000     4.163459    6.076743
                                 |
                        death_30 |
                     nonsurvivor |   6.918299   12.25559     0.56   0.572    -17.10221    30.93881
                                 |
    death_30#c.b_day_malattia |
                     nonsurvivor |   -3.48303   1.480325    -2.35   0.019    -6.384414   -.5816457
                                 |
                           _cons |  -5.791112   4.845078    -1.20   0.232    -15.28729    3.705066
    --------------------------------------------------------------------------------------------

    ------------------------------------------------------------------------------
      Random-effects Parameters  |   Estimate   Std. Err.     [95% Conf. Interval]
    -----------------------------+------------------------------------------------
    progr: Unstructured          |
             var(b_day_malattia) |    35.1686   5.270418      26.21759     47.1756
                       var(_cons) |   1709.433   495.5068        968.54    3017.078
       cov(b_day_malattia,_cons) |  -169.9506   46.90627     -261.8852   -78.01596
    -----------------------------+------------------------------------------------
                    var(Residual) |   2751.337   112.6649      2539.146    2981.259
    ------------------------------------------------------------------------------
    LR test vs. linear model: chi2(3) = 783.15                Prob > chi2 = 0.0000

    Note: LR test is conservative and provided only for reference.

167 . est store linear

168 . mixed eos  c.b_day_malattia##c.b_day_malattia##i.death_30 || progr:b_day_malattia, cov(unstr)

    Performing EM optimization:

    Performing gradient-based optimization:

    Iteration 0:   log likelihood = -10057.824
    Iteration 1:   log likelihood = -10057.642
    Iteration 2:   log likelihood = -10057.642

    Computing standard errors:

    Mixed-effects ML regression                     Number of obs     =      1,805
    Group variable: progr                           Number of groups  =        379

                                                    Obs per group:
                                                                  min =          1
                                                                  avg =        4.8
                                                                  max =         22

                                                    Wald chi2(5)      =     133.34
    Log likelihood = -10057.642                     Prob > chi2       =     0.0000

    -------------------------------------------------------------------------------------------------------------
                                          eos |      Coef.   Std. Err.      z    P>|z|     [95% Conf. Interval]
    -------------------------------------------+-------------------------------------------------------------
                               b_day_malattia |   1.786876   1.338072     1.34   0.182    -.8356974    4.409449
                                              |
              c.b_day_malattia#c.b_day_malattia |   .1433959   .0536253     2.67   0.007     .0382922    .2484996
                                              |
                                     death_30 |
                                  nonsurvivor |   8.559288   17.49322     0.49   0.625     -25.7268    42.84537
                                              |
                      death_30#c.b_day_malattia |
                                  nonsurvivor |   -4.44837   3.378566    -1.32   0.188    -11.07024    2.173497
                                              |
    death_30#c.b_day_malattia#c.b_day_malattia |
                                  nonsurvivor |   .0621388   .1446084     0.43   0.667    -.2212885    .3455661
                                              |
                                        _cons |   10.58238    7.69387     1.38   0.169    -4.497329    25.66209
    -------------------------------------------------------------------------------------------------------------

    ------------------------------------------------------------------------------
      Random-effects Parameters  |   Estimate   Std. Err.     [95% Conf. Interval]
    -----------------------------+------------------------------------------------
    progr: Unstructured          |
```

```
        var(b_day_malattia) |   34.48418   5.193817      25.66946    46.32583
                  var(_cons) |   1377.686    461.892      714.1199    2657.845
   cov(b_day_malattia,_cons) |  -150.8955   44.79085     -238.6839   -63.10701
-----------------------------+------------------------------------------------
               var(Residual) |   2763.249   112.9161      2550.569    2993.665
------------------------------------------------------------------------------
LR test vs. linear model: chi2(3) = 786.76                Prob > chi2 = 0.0000

Note: LR test is conservative and provided only for reference.
```

```
169 . est store quadratic

170 . lrtest linear quadratic

    Likelihood-ratio test                                 LR chi2(2)  =      8.79
    (Assumption: linear nested in quadratic)              Prob > chi2 =    0.0124

171 .
172 . margins death_30, at(b_day_malattia=(0(1)30))

    Adjusted predictions                            Number of obs     =      1,805

    Expression   : Linear prediction, fixed portion, predict()

    1._at        : b_day_mala~a    =           0

    2._at        : b_day_mala~a    =           1

    3._at        : b_day_mala~a    =           2

    4._at        : b_day_mala~a    =           3

    5._at        : b_day_mala~a    =           4

    6._at        : b_day_mala~a    =           5

    7._at        : b_day_mala~a    =           6

    8._at        : b_day_mala~a    =           7

    9._at        : b_day_mala~a    =           8

    10._at       : b_day_mala~a    =           9

    11._at       : b_day_mala~a    =          10

    12._at       : b_day_mala~a    =          11

    13._at       : b_day_mala~a    =          12

    14._at       : b_day_mala~a    =          13

    15._at       : b_day_mala~a    =          14

    16._at       : b_day_mala~a    =          15

    17._at       : b_day_mala~a    =          16

    18._at       : b_day_mala~a    =          17

    19._at       : b_day_mala~a    =          18

    20._at       : b_day_mala~a    =          19

    21._at       : b_day_mala~a    =          20

    22._at       : b_day_mala~a    =          21

    23._at       : b_day_mala~a    =          22

    24._at       : b_day_mala~a    =          23

    25._at       : b_day_mala~a    =          24

    26._at       : b_day_mala~a    =          25

    27._at       : b_day_mala~a    =          26

    28._at       : b_day_mala~a    =          27

    29._at       : b_day_mala~a    =          28

    30._at       : b_day_mala~a    =          29

    31._at       : b_day_mala~a    =          30
```

	Margin	Delta-method Std. Err.	z	P>|z|	[95% Conf.	Interval]
_at#death_30						
1#survivor	10.58238	7.69387	1.38	0.169	-4.497329	25.66209
1#nonsurvivor	19.14167	15.71042	1.22	0.223	-11.65018	49.93352
2#survivor	12.51265	6.527092	1.92	0.055	-.2802154	25.30552
2#nonsurvivor	16.68571	13.12093	1.27	0.203	-9.030837	42.40225
3#survivor	14.72971	5.48991	2.68	0.007	3.969687	25.48974
3#nonsurvivor	14.64082	10.89322	1.34	0.179	-6.709501	35.99113
4#survivor	17.23357	4.593984	3.75	0.000	8.229525	26.23761
4#nonsurvivor	13.007	9.083217	1.43	0.152	-4.795783	30.80977
5#survivor	20.02422	3.856058	5.19	0.000	12.46648	27.58195
5#nonsurvivor	11.78424	7.765083	1.52	0.129	-3.435039	27.00353
6#survivor	23.10165	3.29714	7.01	0.000	16.63938	29.56393
6#nonsurvivor	10.97256	7.005717	1.57	0.117	-2.758394	24.70351
7#survivor	26.46588	2.93566	9.02	0.000	20.7121	32.21967
7#nonsurvivor	10.57195	6.806705	1.55	0.120	-2.76895	23.91284
8#survivor	30.11691	2.772483	10.86	0.000	24.68294	35.55087
8#nonsurvivor	10.5824	7.068136	1.50	0.134	-3.270889	24.43569
9#survivor	34.05472	2.778922	12.25	0.000	28.60813	39.50131

9#nonsurvivor	11.00393	7.635667	1.44	0.150	-3.961704	25.96956
10#survivor	38.27933	2.905158	13.18	0.000	32.58532	43.97333
10#nonsurvivor	11.83652	8.37497	1.41	0.158	-4.578117	28.25116
11#survivor	42.79072	3.102079	13.79	0.000	36.71076	48.87069
11#nonsurvivor	13.08019	9.201946	1.42	0.155	-4.955297	31.11567
12#survivor	47.58891	3.335251	14.27	0.000	41.05194	54.12589
12#nonsurvivor	14.73492	10.07723	1.46	0.144	-5.016082	34.48592
13#survivor	52.67389	3.586728	14.69	0.000	45.64404	59.70375
13#nonsurvivor	16.80072	10.99259	1.53	0.126	-4.744359	38.3458
14#survivor	58.04567	3.851715	15.07	0.000	50.49644	65.59489
14#nonsurvivor	19.27759	11.95998	1.61	0.107	-4.16354	42.71873
15#survivor	63.70423	4.134837	15.41	0.000	55.6001	71.80836
15#nonsurvivor	22.16553	13.0039	1.70	0.088	-3.321644	47.65271
16#survivor	69.64959	4.447113	15.66	0.000	60.93341	78.36577
16#nonsurvivor	25.46455	14.1559	1.80	0.072	-2.2805	53.20959
17#survivor	75.88174	4.803461	15.80	0.000	66.46712	85.29635
17#nonsurvivor	29.17462	15.45022	1.89	0.059	-1.107245	59.45649
18#survivor	82.40068	5.220431	15.78	0.000	72.16882	92.63253
18#nonsurvivor	33.29577	16.92035	1.97	0.049	.1325	66.45905
19#survivor	89.20641	5.714153	15.61	0.000	78.00687	100.4059
19#nonsurvivor	37.82799	18.59641	2.03	0.042	1.379706	74.27628
20#survivor	96.29893	6.298725	15.29	0.000	83.95366	108.6442
20#nonsurvivor	42.77128	20.50357	2.09	0.037	2.585015	82.95754
21#survivor	103.6782	6.985261	14.84	0.000	89.98739	117.3691
21#nonsurvivor	48.12564	22.66152	2.12	0.034	3.709873	92.5414
22#survivor	111.3444	7.781654	14.31	0.000	96.09259	126.5961
22#nonsurvivor	53.89106	25.08464	2.15	0.032	4.726081	103.056
23#survivor	119.2973	8.692903	13.72	0.000	102.2595	136.335
23#nonsurvivor	60.06756	27.78275	2.16	0.031	5.614365	114.5208
24#survivor	127.5369	9.721727	13.12	0.000	108.4827	146.5912
24#nonsurvivor	66.65512	30.76209	2.17	0.030	6.362542	126.9477
25#survivor	136.0634	10.86923	12.52	0.000	114.7601	157.3667
25#nonsurvivor	73.65376	34.02617	2.16	0.030	6.963694	140.3438
26#survivor	144.8767	12.13549	11.94	0.000	121.0916	168.6618
26#nonsurvivor	81.06346	37.57664	2.16	0.031	7.414602	154.7123
27#survivor	153.9768	13.51997	11.39	0.000	127.4781	180.4754
27#nonsurvivor	88.88424	41.41389	2.15	0.032	7.714508	170.054
28#survivor	163.3636	15.02184	10.88	0.000	133.9214	192.8059
28#nonsurvivor	97.11608	45.53751	2.13	0.033	7.864204	186.368
29#survivor	173.0373	16.64014	10.40	0.000	140.4232	205.6513
29#nonsurvivor	105.759	49.94663	2.12	0.034	7.86539	203.6526
30#survivor	182.9977	18.37389	9.96	0.000	146.9855	219.0099
30#nonsurvivor	114.813	54.64015	2.10	0.036	7.720245	221.9057
31#survivor	193.2449	20.22219	9.56	0.000	153.6102	232.8797
31#nonsurvivor	124.278	59.61685	2.08	0.037	7.431142	241.1249

173 . marginsplot, title("Eosinophils kinetic day 0 to day 28 after onset") ytitle("Cells per mmc (log-scale)")

Variables that uniquely identify margins: b_day_malattia death_30

174 . margins ar.death_30, at(b_day_malattia=(0(1)30))

Contrasts of adjusted predictions Number of obs = **1,805**

Expression : **Linear prediction, fixed portion, predict()**

1._at : b_day_mala~a = **0**

2._at : b_day_mala~a = **1**

3._at : b_day_mala~a = **2**

4._at : b_day_mala~a = **3**

5._at : b_day_mala~a = **4**

6._at : b_day_mala~a = **5**

7._at : b_day_mala~a = **6**

8._at : b_day_mala~a = **7**

9._at : b_day_mala~a = **8**

10._at : b_day_mala~a = **9**

11._at : b_day_mala~a = **10**

12._at : b_day_mala~a = **11**

13._at : b_day_mala~a = **12**

14._at : b_day_mala~a = **13**

15._at : b_day_mala~a = **14**

16._at : b_day_mala~a = **15**

17._at : b_day_mala~a = **16**

18._at : b_day_mala~a = **17**

19._at : b_day_mala~a = **18**

20._at : b_day_mala~a = **19**

21._at : b_day_mala~a = **20**

22._at : b_day_mala~a = **21**

23._at : b_day_mala~a = **22**

24._at : b_day_mala~a = **23**

25._at : b_day_mala~a = **24**

26._at : b_day_mala~a = **25**

```
27._at       : b_day_mala~a    =          26

28._at       : b_day_mala~a    =          27

29._at       : b_day_mala~a    =          28

30._at       : b_day_mala~a    =          29

31._at       : b_day_mala~a    =          30
```

	df	chi2	P>chi2
death_30@_at			
(nonsurvivor vs survivor) 1	1	0.24	0.6246
(nonsurvivor vs survivor) 2	1	0.08	0.7758
(nonsurvivor vs survivor) 3	1	0.00	0.9942
(nonsurvivor vs survivor) 4	1	0.17	0.6780
(nonsurvivor vs survivor) 5	1	0.90	0.3419
(nonsurvivor vs survivor) 6	1	2.45	0.1172
(nonsurvivor vs survivor) 7	1	4.60	0.0320
(nonsurvivor vs survivor) 8	1	6.62	0.0101
(nonsurvivor vs survivor) 9	1	8.05	0.0046
(nonsurvivor vs survivor) 10	1	8.90	0.0029
(nonsurvivor vs survivor) 11	1	9.36	0.0022
(nonsurvivor vs survivor) 12	1	9.58	0.0020
(nonsurvivor vs survivor) 13	1	9.63	0.0019
(nonsurvivor vs survivor) 14	1	9.52	0.0020
(nonsurvivor vs survivor) 15	1	9.27	0.0023
(nonsurvivor vs survivor) 16	1	8.87	0.0029
(nonsurvivor vs survivor) 17	1	8.33	0.0039
(nonsurvivor vs survivor) 18	1	7.69	0.0056
(nonsurvivor vs survivor) 19	1	6.97	0.0083
(nonsurvivor vs survivor) 20	1	6.23	0.0126
(nonsurvivor vs survivor) 21	1	5.49	0.0191
(nonsurvivor vs survivor) 22	1	4.79	0.0287
(nonsurvivor vs survivor) 23	1	4.14	0.0419
(nonsurvivor vs survivor) 24	1	3.56	0.0591
(nonsurvivor vs survivor) 25	1	3.05	0.0806
(nonsurvivor vs survivor) 26	1	2.61	0.1061
(nonsurvivor vs survivor) 27	1	2.23	0.1351
(nonsurvivor vs survivor) 28	1	1.91	0.1671
(nonsurvivor vs survivor) 29	1	1.63	0.2013
(nonsurvivor vs survivor) 30	1	1.40	0.2369
(nonsurvivor vs survivor) 31	1	1.20	0.2733
Joint	3	9.82	0.0202

	Contrast	Delta-method Std. Err.	[95% Conf.	Interval]
death_30@_at				
(nonsurvivor vs survivor) 1	8.559288	17.49322	-25.7268	42.84537
(nonsurvivor vs survivor) 2	4.173057	14.65475	-24.54972	32.89584
(nonsurvivor vs survivor) 3	-.0888971	12.19842	-23.99735	23.81956
(nonsurvivor vs survivor) 4	-4.226574	10.17888	-24.1768	15.72366
(nonsurvivor vs survivor) 5	-8.239972	8.669815	-25.2325	8.752552
(nonsurvivor vs survivor) 6	-12.12909	7.742817	-27.30474	3.046548
(nonsurvivor vs survivor) 7	-15.89394	7.412782	-30.42272	-1.365152
(nonsurvivor vs survivor) 8	-19.5345	7.592444	-34.41542	-4.653587
(nonsurvivor vs survivor) 9	-23.05079	8.125627	-38.97673	-7.124856
(nonsurvivor vs survivor) 10	-26.4428	8.86454	-43.81698	-9.068625
(nonsurvivor vs survivor) 11	-29.71054	9.710752	-48.74326	-10.67781
(nonsurvivor vs survivor) 12	-32.85399	10.61482	-53.65866	-12.04933
(nonsurvivor vs survivor) 13	-35.87317	11.56294	-58.53612	-13.21022
(nonsurvivor vs survivor) 14	-38.76807	12.56491	-63.39484	-14.14131
(nonsurvivor vs survivor) 15	-41.5387	13.64545	-68.28329	-14.7941
(nonsurvivor vs survivor) 16	-44.18504	14.838	-73.26698	-15.1031
(nonsurvivor vs survivor) 17	-46.70711	16.17969	-78.41873	-14.9955
(nonsurvivor vs survivor) 18	-49.1049	17.70737	-83.81072	-14.39909
(nonsurvivor vs survivor) 19	-51.37842	19.45451	-89.50855	-13.24828
(nonsurvivor vs survivor) 20	-53.52765	21.44925	-95.56741	-11.48789
(nonsurvivor vs survivor) 21	-55.55261	23.71367	-102.0306	-9.074661
(nonsurvivor vs survivor) 22	-57.45329	26.26391	-108.9296	-5.976968
(nonsurvivor vs survivor) 23	-59.22969	29.11096	-116.2861	-2.173264
(nonsurvivor vs survivor) 24	-60.88182	32.26171	-124.1136	2.349971
(nonsurvivor vs survivor) 25	-62.40967	35.72003	-132.4196	7.600299
(nonsurvivor vs survivor) 26	-63.81324	39.48764	-141.2076	13.58112
(nonsurvivor vs survivor) 27	-65.09253	43.56489	-150.4781	20.29309
(nonsurvivor vs survivor) 28	-66.24755	47.95123	-160.2302	27.73513
(nonsurvivor vs survivor) 29	-67.27828	52.64561	-170.4618	35.90521
(nonsurvivor vs survivor) 30	-68.18475	57.64673	-181.1703	44.80078
(nonsurvivor vs survivor) 31	-68.96693	62.9532	-192.3529	54.41908

```
175 .
176 .
177 . ***************************************************************************
178 . *** analisi PLTS  **************************************************
179 . ***************************************************************************
180 . **** scatter no model
181 . use "/Users/Chiara/Documents/fileDO 15 Maggio/fileDO\coorte_long_emocromo_anemia_1.dta", clear

182 . *scatter plt b_day_malattia  if death_30==1, mcolor(red) legend(off) title("Platelets variation over time") xtitle
    > ("day since onset") ytitle("Cells per mmc X 1000") xlab(0(5)30)|| scatter plt b_day_malattia  if death_30==0, mcol
    > or(green) legend(off) ms(oh)
183 . *********Graph box**************************************
184 . replace plt=plt*1000
    (1,805 real changes made)

185 . graph box plt, over(death_30, label(nolabel)) over(b_day_malattia) asyvars box(1, fcolor(navy))  nooutsides ytitle
    > (Cells per mmc) yline(150000, lcolor(red)) legend(off)

186 . graph save "Graph" "/Users/Chiara/Documents/fileDO 15 Maggio/BOX_PLT.gph", replace
    (file /Users/Chiara/Documents/fileDO 15 Maggio/BOX_PLT.gph saved)
```

```
187 . **** modelling
188 . use "/Users/Chiara/Documents/fileDO 15 Maggio/fileDO\coorte_long_emocromo_anemia_1.dta", clear

189 . mixed plt_ln c.b_day_malattia##i.death_30 || progr:b_day_malattia, cov(unstr)

    Performing EM optimization:

    Performing gradient-based optimization:

    Iteration 0:   log likelihood = -488.15169
    Iteration 1:   log likelihood = -488.15166

    Computing standard errors:

    Mixed-effects ML regression                     Number of obs     =      1,805
    Group variable: progr                           Number of groups  =        379

                                                    Obs per group:
                                                                  min =          1
                                                                  avg =        4.8
                                                                  max =         22

                                                    Wald chi2(3)      =     170.93
    Log likelihood = -488.15166                     Prob > chi2       =     0.0000

    ------------------------------------------------------------------------------------------
                       plt_ln |      Coef.   Std. Err.      z    P>|z|     [95% Conf. Interval]
    --------------------------+---------------------------------------------------------------
               b_day_malattia |    .040182   .0032144    12.50   0.000      .033882     .046482
                              |
                     death_30 |
                  nonsurvivor |   .3307512    .117088     2.82   0.005     .1012631    .5602394
                              |
    death_30#c.b_day_malattia |
                  nonsurvivor |  -.0509723   .0096373    -5.29   0.000     -.069861   -.0320836
                              |
                        _cons |   11.96795   .0419482   285.30   0.000     11.88573    12.05017
    ------------------------------------------------------------------------------------------

    ------------------------------------------------------------------------------
      Random-effects Parameters  |   Estimate   Std. Err.     [95% Conf. Interval]
    -----------------------------+------------------------------------------------
    progr: Unstructured          |
             var(b_day_malattia) |   .0021197   .0002226      .0017254    .0026041
                       var(_cons) |   .3864737   .0394638      .3163746    .4721047
       cov(b_day_malattia,_cons) |  -.0240338   .0027549     -.0294332   -.0186343
    -----------------------------+------------------------------------------------
                   var(Residual) |   .0483518    .002008      .0445721     .052452
    ------------------------------------------------------------------------------
    LR test vs. linear model: chi2(3) = 1364.85               Prob > chi2 = 0.0000

    Note: LR test is conservative and provided only for reference.

190 . est store linear

191 . mixed plt_ln  c.b_day_malattia##c.b_day_malattia##i.death_30 i.p_age2 i.p_cardio i.p_obeso i.p_rene|| progr:b_day_
    > malattia, cov(unstr)

    Performing EM optimization:

    Performing gradient-based optimization:

    Iteration 0:   log likelihood = -382.02189
    Iteration 1:   log likelihood = -382.02185

    Computing standard errors:

    Mixed-effects ML regression                     Number of obs     =      1,805
    Group variable: progr                           Number of groups  =        379

                                                    Obs per group:
                                                                  min =          1
                                                                  avg =        4.8
                                                                  max =         22

                                                    Wald chi2(9)      =     413.99
    Log likelihood = -382.02185                     Prob > chi2       =     0.0000

    -------------------------------------------------------------------------------------------------------------
                                          plt_ln |      Coef.   Std. Err.      z    P>|z|     [95% Conf. Interval]
    ---------------------------------------------+---------------------------------------------------------------
                                  b_day_malattia |   .1209497   .0070774    17.09   0.000     .1070783    .1348211
                                                 |
                c.b_day_malattia#c.b_day_malattia |  -.0032354   .0002557   -12.65   0.000    -.0037367   -.0027342
                                                 |
                                        death_30 |
                                     nonsurvivor |   .3947662   .1463285     2.70   0.007     .1079676    .6815648
                                                 |
                        death_30#c.b_day_malattia |
                                     nonsurvivor |  -.0276821   .0179166    -1.55   0.122     -.062798    .0074338
                                                 |
    death_30#c.b_day_malattia#c.b_day_malattia |
                                     nonsurvivor |   -.001778    .000696    -2.55   0.011    -.0031422   -.0004138
                                                 |
                                        1.p_age2 |  -.1391985   .0420066    -3.31   0.001      -.22153   -.0568671
                                      1.p_cardio |  -.0182412   .0448995    -0.41   0.685    -.1062426    .0697603
                                       1.p_obeso |   .0614358   .0794493     0.77   0.439     -.094282    .2171537
                                        1.p_rene |  -.1097104   .0927053    -1.18   0.237    -.2914094    .0719886
                                           _cons |    11.5935   .0604623   191.75   0.000     11.47499      11.712
    -------------------------------------------------------------------------------------------------------------

    ------------------------------------------------------------------------------
      Random-effects Parameters  |   Estimate   Std. Err.     [95% Conf. Interval]
    -----------------------------+------------------------------------------------
    progr: Unstructured          |
             var(b_day_malattia) |   .0024137   .0002437      .0019804    .0029418
                       var(_cons) |   .4782215   .0476246      .3934237    .5812963
       cov(b_day_malattia,_cons) |  -.0297081   .0032096     -.0359988   -.0234174
```

```
               var(Residual)  |   .0398844   .0016814      .0367214    .0433198

LR test vs. linear model: chi2(3) = 1477.93              Prob > chi2 = 0.0000

Note: LR test is conservative and provided only for reference.
```

```
192 . est store quadratic

193 . lrtest linear quadratic

    Likelihood-ratio test                                 LR chi2(6)  =    212.26
    (Assumption: linear nested in quadratic)              Prob > chi2 =    0.0000

194 . *margins***********
195 . quiet: mixed plt_ln  c.b_day_malattia##c.b_day_malattia##i.death_30 i.p_age2 i.p_cardio i.p_obeso i.p_rene|| progr
    > :b_day_malattia, cov(unstr)

196 . margins death_30, at(b_day_malattia=(0(1)21))  expression(exp(predict(xb))) asbal

    Adjusted predictions                            Number of obs     =      1,805

    Expression   : exp(predict(xb))

    1._at        : b_day_mala~a    =           0
                   death_30                      (asbalanced)
                   p_age2                        (asbalanced)
                   p_cardio                      (asbalanced)
                   p_obeso                       (asbalanced)
                   p_rene                        (asbalanced)

    2._at        : b_day_mala~a    =           1
                   death_30                      (asbalanced)
                   p_age2                        (asbalanced)
                   p_cardio                      (asbalanced)
                   p_obeso                       (asbalanced)
                   p_rene                        (asbalanced)

    3._at        : b_day_mala~a    =           2
                   death_30                      (asbalanced)
                   p_age2                        (asbalanced)
                   p_cardio                      (asbalanced)
                   p_obeso                       (asbalanced)
                   p_rene                        (asbalanced)

    4._at        : b_day_mala~a    =           3
                   death_30                      (asbalanced)
                   p_age2                        (asbalanced)
                   p_cardio                      (asbalanced)
                   p_obeso                       (asbalanced)
                   p_rene                        (asbalanced)

    5._at        : b_day_mala~a    =           4
                   death_30                      (asbalanced)
                   p_age2                        (asbalanced)
                   p_cardio                      (asbalanced)
                   p_obeso                       (asbalanced)
                   p_rene                        (asbalanced)

    6._at        : b_day_mala~a    =           5
                   death_30                      (asbalanced)
                   p_age2                        (asbalanced)
                   p_cardio                      (asbalanced)
                   p_obeso                       (asbalanced)
                   p_rene                        (asbalanced)

    7._at        : b_day_mala~a    =           6
                   death_30                      (asbalanced)
                   p_age2                        (asbalanced)
                   p_cardio                      (asbalanced)
                   p_obeso                       (asbalanced)
                   p_rene                        (asbalanced)

    8._at        : b_day_mala~a    =           7
                   death_30                      (asbalanced)
                   p_age2                        (asbalanced)
                   p_cardio                      (asbalanced)
                   p_obeso                       (asbalanced)
                   p_rene                        (asbalanced)

    9._at        : b_day_mala~a    =           8
                   death_30                      (asbalanced)
                   p_age2                        (asbalanced)
                   p_cardio                      (asbalanced)
                   p_obeso                       (asbalanced)
                   p_rene                        (asbalanced)

    10._at       : b_day_mala~a    =           9
                   death_30                      (asbalanced)
                   p_age2                        (asbalanced)
                   p_cardio                      (asbalanced)
                   p_obeso                       (asbalanced)
                   p_rene                        (asbalanced)

    11._at       : b_day_mala~a    =          10
                   death_30                      (asbalanced)
                   p_age2                        (asbalanced)
                   p_cardio                      (asbalanced)
                   p_obeso                       (asbalanced)
                   p_rene                        (asbalanced)

    12._at       : b_day_mala~a    =          11
                   death_30                      (asbalanced)
                   p_age2                        (asbalanced)
                   p_cardio                      (asbalanced)
                   p_obeso                       (asbalanced)
                   p_rene                        (asbalanced)
```

```
13._at       : b_day_mala~a    =          12
               death_30                      (asbalanced)
               p_age2                        (asbalanced)
               p_cardio                      (asbalanced)
               p_obeso                       (asbalanced)
               p_rene                        (asbalanced)

14._at       : b_day_mala~a    =          13
               death_30                      (asbalanced)
               p_age2                        (asbalanced)
               p_cardio                      (asbalanced)
               p_obeso                       (asbalanced)
               p_rene                        (asbalanced)

15._at       : b_day_mala~a    =          14
               death_30                      (asbalanced)
               p_age2                        (asbalanced)
               p_cardio                      (asbalanced)
               p_obeso                       (asbalanced)
               p_rene                        (asbalanced)

16._at       : b_day_mala~a    =          15
               death_30                      (asbalanced)
               p_age2                        (asbalanced)
               p_cardio                      (asbalanced)
               p_obeso                       (asbalanced)
               p_rene                        (asbalanced)

17._at       : b_day_mala~a    =          16
               death_30                      (asbalanced)
               p_age2                        (asbalanced)
               p_cardio                      (asbalanced)
               p_obeso                       (asbalanced)
               p_rene                        (asbalanced)

18._at       : b_day_mala~a    =          17
               death_30                      (asbalanced)
               p_age2                        (asbalanced)
               p_cardio                      (asbalanced)
               p_obeso                       (asbalanced)
               p_rene                        (asbalanced)

19._at       : b_day_mala~a    =          18
               death_30                      (asbalanced)
               p_age2                        (asbalanced)
               p_cardio                      (asbalanced)
               p_obeso                       (asbalanced)
               p_rene                        (asbalanced)

20._at       : b_day_mala~a    =          19
               death_30                      (asbalanced)
               p_age2                        (asbalanced)
               p_cardio                      (asbalanced)
               p_obeso                       (asbalanced)
               p_rene                        (asbalanced)

21._at       : b_day_mala~a    =          20
               death_30                      (asbalanced)
               p_age2                        (asbalanced)
               p_cardio                      (asbalanced)
               p_obeso                       (asbalanced)
               p_rene                        (asbalanced)

22._at       : b_day_mala~a    =          21
               death_30                      (asbalanced)
               p_age2                        (asbalanced)
               p_cardio                      (asbalanced)
               p_obeso                       (asbalanced)
               p_rene                        (asbalanced)
```

	Margin	Delta-method Std. Err.	z	P>|z|	[95% Conf.	Interval]
_at#death_30						
1#survivor	97796.27	7888.64	12.40	0.000	82334.82	113257.7
1#nonsurvivor	145133.3	20108.02	7.22	0.000	105722.3	184544.3
2#survivor	110013.2	8428.929	13.05	0.000	93492.84	126533.6
2#nonsurvivor	158524.1	20066.9	7.90	0.000	119193.7	197854.5
3#survivor	122958.1	8994.522	13.67	0.000	105329.2	140587.1
3#nonsurvivor	171423	19879.56	8.62	0.000	132459.8	210386.2
4#survivor	136539.8	9589.321	14.24	0.000	117745.1	155334.6
4#nonsurvivor	183522	19576.81	9.37	0.000	145152.2	221891.9
5#survivor	150643.8	10215.87	14.75	0.000	130621	170666.5
5#nonsurvivor	194514.8	19192.27	10.14	0.000	156898.6	232131
6#survivor	165132.6	10874.62	15.19	0.000	143818.7	186446.5
6#nonsurvivor	204109.2	18758.93	10.88	0.000	167342.4	240876
7#survivor	179847.4	11563.37	15.55	0.000	157183.6	202511.2
7#nonsurvivor	212040	18306.1	11.58	0.000	176160.7	247919.3
8#survivor	194610	12277.05	15.85	0.000	170547.4	218672.6
8#nonsurvivor	218081.4	17857.47	12.21	0.000	183081.4	253081.4
9#survivor	209226.1	13007.93	16.08	0.000	183731	234721.2
9#nonsurvivor	222057.1	17430.51	12.74	0.000	187893.9	256220.3
10#survivor	223489.1	13746.11	16.26	0.000	196547.2	250431
10#nonsurvivor	223849.6	17037.45	13.14	0.000	190456.8	257242.3
11#survivor	237184.6	14480.38	16.38	0.000	208803.6	265565.6
11#nonsurvivor	223405.1	16686.9	13.39	0.000	190699.4	256110.9
12#survivor	250095.8	15199.22	16.45	0.000	220305.9	279885.8
12#nonsurvivor	220737.2	16385.54	13.47	0.000	188622.1	252852.3
13#survivor	262009	15891.95	16.49	0.000	230861.3	293156.6
13#nonsurvivor	215925.1	16138.79	13.38	0.000	184293.7	247556.6
14#survivor	272719.1	16549.79	16.48	0.000	240282.1	305156.1
14#nonsurvivor	209110.7	15949.96	13.11	0.000	177849.4	240372.1
15#survivor	282036.1	17166.72	16.43	0.000	248389.9	315682.2
15#nonsurvivor	200491	15818.33	12.67	0.000	169487.7	231494.4
16#survivor	289790.1	17740.12	16.34	0.000	255020.1	324560.1
16#nonsurvivor	190308.8	15736.7	12.09	0.000	159465.4	221152.1
17#survivor	295836.7	18270.98	16.19	0.000	260026.3	331647.2

```
 17#nonsurvivor |   178841.5    15689.9    11.40   0.000     148089.8    209593.1
    18#survivor |   300061.6   18763.61    15.99   0.000     263285.6    336837.6
 18#nonsurvivor |   166388.4   15654.94    10.63   0.000     135705.2    197071.5
    19#survivor |   302383.8   19224.92    15.73   0.000     264703.6    340063.9
 19#nonsurvivor |     153258   15603.02     9.82   0.000     122676.6    183839.3
    20#survivor |   302758.4   19663.19    15.40   0.000     264219.3    341297.6
 20#nonsurvivor |   139755.4   15503.05     9.01   0.000       109370    170140.8
    21#survivor |   301178.4   20086.47    14.99   0.000     261809.6    340547.1
 21#nonsurvivor |     126171   15325.54     8.23   0.000     96133.52    156208.5
    22#survivor |   297674.1   20500.92    14.52   0.000       257493    337855.1
 22#nonsurvivor |   112770.6   15046.18     7.49   0.000     83280.66    142260.6
--------------------------------------------------------------------------------

197 . marginsplot, ytitle("Cells per mmc") xlab(0(1)21) ylab(0(200000)800000, angle(horizontal)) yline(150000,lcolor(red
> )) legend(off)

  Variables that uniquely identify margins: b_day_malattia death_30

198 . margins ar.death_30, at(b_day_malattia=(0(1)21))  expression(exp(predict(xb))) asbal

Contrasts of adjusted predictions               Number of obs     =      1,805

Expression   : exp(predict(xb))

1._at        : b_day_mala~a    =           0
               death_30                     (asbalanced)
               p_age2                       (asbalanced)
               p_cardio                     (asbalanced)
               p_obeso                      (asbalanced)
               p_rene                       (asbalanced)

2._at        : b_day_mala~a    =           1
               death_30                     (asbalanced)
               p_age2                       (asbalanced)
               p_cardio                     (asbalanced)
               p_obeso                      (asbalanced)
               p_rene                       (asbalanced)

3._at        : b_day_mala~a    =           2
               death_30                     (asbalanced)
               p_age2                       (asbalanced)
               p_cardio                     (asbalanced)
               p_obeso                      (asbalanced)
               p_rene                       (asbalanced)

4._at        : b_day_mala~a    =           3
               death_30                     (asbalanced)
               p_age2                       (asbalanced)
               p_cardio                     (asbalanced)
               p_obeso                      (asbalanced)
               p_rene                       (asbalanced)

5._at        : b_day_mala~a    =           4
               death_30                     (asbalanced)
               p_age2                       (asbalanced)
               p_cardio                     (asbalanced)
               p_obeso                      (asbalanced)
               p_rene                       (asbalanced)

6._at        : b_day_mala~a    =           5
               death_30                     (asbalanced)
               p_age2                       (asbalanced)
               p_cardio                     (asbalanced)
               p_obeso                      (asbalanced)
               p_rene                       (asbalanced)

7._at        : b_day_mala~a    =           6
               death_30                     (asbalanced)
               p_age2                       (asbalanced)
               p_cardio                     (asbalanced)
               p_obeso                      (asbalanced)
               p_rene                       (asbalanced)

8._at        : b_day_mala~a    =           7
               death_30                     (asbalanced)
               p_age2                       (asbalanced)
               p_cardio                     (asbalanced)
               p_obeso                      (asbalanced)
               p_rene                       (asbalanced)

9._at        : b_day_mala~a    =           8
               death_30                     (asbalanced)
               p_age2                       (asbalanced)
               p_cardio                     (asbalanced)
               p_obeso                      (asbalanced)
               p_rene                       (asbalanced)

10._at       : b_day_mala~a    =           9
               death_30                     (asbalanced)
               p_age2                       (asbalanced)
               p_cardio                     (asbalanced)
               p_obeso                      (asbalanced)
               p_rene                       (asbalanced)

11._at       : b_day_mala~a    =          10
               death_30                     (asbalanced)
               p_age2                       (asbalanced)
               p_cardio                     (asbalanced)
               p_obeso                      (asbalanced)
               p_rene                       (asbalanced)

12._at       : b_day_mala~a    =          11
               death_30                     (asbalanced)
               p_age2                       (asbalanced)
               p_cardio                     (asbalanced)
               p_obeso                      (asbalanced)
               p_rene                       (asbalanced)
```

```
13._at       : b_day_mala~a    =          12
               death_30                      (asbalanced)
               p_age2                        (asbalanced)
               p_cardio                      (asbalanced)
               p_obeso                       (asbalanced)
               p_rene                        (asbalanced)

14._at       : b_day_mala~a    =          13
               death_30                      (asbalanced)
               p_age2                        (asbalanced)
               p_cardio                      (asbalanced)
               p_obeso                       (asbalanced)
               p_rene                        (asbalanced)

15._at       : b_day_mala~a    =          14
               death_30                      (asbalanced)
               p_age2                        (asbalanced)
               p_cardio                      (asbalanced)
               p_obeso                       (asbalanced)
               p_rene                        (asbalanced)

16._at       : b_day_mala~a    =          15
               death_30                      (asbalanced)
               p_age2                        (asbalanced)
               p_cardio                      (asbalanced)
               p_obeso                       (asbalanced)
               p_rene                        (asbalanced)

17._at       : b_day_mala~a    =          16
               death_30                      (asbalanced)
               p_age2                        (asbalanced)
               p_cardio                      (asbalanced)
               p_obeso                       (asbalanced)
               p_rene                        (asbalanced)

18._at       : b_day_mala~a    =          17
               death_30                      (asbalanced)
               p_age2                        (asbalanced)
               p_cardio                      (asbalanced)
               p_obeso                       (asbalanced)
               p_rene                        (asbalanced)

19._at       : b_day_mala~a    =          18
               death_30                      (asbalanced)
               p_age2                        (asbalanced)
               p_cardio                      (asbalanced)
               p_obeso                       (asbalanced)
               p_rene                        (asbalanced)

20._at       : b_day_mala~a    =          19
               death_30                      (asbalanced)
               p_age2                        (asbalanced)
               p_cardio                      (asbalanced)
               p_obeso                       (asbalanced)
               p_rene                        (asbalanced)

21._at       : b_day_mala~a    =          20
               death_30                      (asbalanced)
               p_age2                        (asbalanced)
               p_cardio                      (asbalanced)
               p_obeso                       (asbalanced)
               p_rene                        (asbalanced)

22._at       : b_day_mala~a    =          21
               death_30                      (asbalanced)
               p_age2                        (asbalanced)
               p_cardio                      (asbalanced)
               p_obeso                       (asbalanced)
               p_rene                        (asbalanced)
```

	df	chi2	P>chi2
death_30@_at			
(nonsurvivor vs survivor) 1	1	5.52	0.0188
(nonsurvivor vs survivor) 2	1	5.90	0.0151
(nonsurvivor vs survivor) 3	1	6.09	0.0136
(nonsurvivor vs survivor) 4	1	6.01	0.0142
(nonsurvivor vs survivor) 5	1	5.55	0.0184
(nonsurvivor vs survivor) 6	1	4.67	0.0306
(nonsurvivor vs survivor) 7	1	3.40	0.0650
(nonsurvivor vs survivor) 8	1	1.92	0.1657
(nonsurvivor vs survivor) 9	1	0.60	0.4372
(nonsurvivor vs survivor) 10	1	0.00	0.9823
(nonsurvivor vs survivor) 11	1	0.73	0.3925
(nonsurvivor vs survivor) 12	1	3.29	0.0695
(nonsurvivor vs survivor) 13	1	7.88	0.0050
(nonsurvivor vs survivor) 14	1	14.31	0.0002
(nonsurvivor vs survivor) 15	1	22.04	0.0000
(nonsurvivor vs survivor) 16	1	30.41	0.0000
(nonsurvivor vs survivor) 17	1	38.78	0.0000
(nonsurvivor vs survivor) 18	1	46.63	0.0000
(nonsurvivor vs survivor) 19	1	53.62	0.0000
(nonsurvivor vs survivor) 20	1	59.54	0.0000
(nonsurvivor vs survivor) 21	1	64.30	0.0000
(nonsurvivor vs survivor) 22	1	67.86	0.0000
Joint	6	314.32	0.0000

	Contrast	Delta-method Std. Err.	[95% Conf.	Interval]
death_30@_at				
(nonsurvivor vs survivor) 1	47337.03	20141.19	7861.019	86813.05
(nonsurvivor vs survivor) 2	48510.89	19970.61	9369.225	87652.56
(nonsurvivor vs survivor) 3	48464.84	19633.79	9983.324	86946.37
(nonsurvivor vs survivor) 4	46982.17	19167.56	9414.436	84549.9

```
(nonsurvivor vs survivor)  5 |   43871.02   18615.81      7384.695    80357.34
(nonsurvivor vs survivor)  6 |    38976.6   18026.66          3645    74308.21
(nonsurvivor vs survivor)  7 |   32192.67   17449.34     -2007.41    66392.76
(nonsurvivor vs survivor)  8 |   23471.41   16931.11     -9712.962    56655.77
(nonsurvivor vs survivor)  9 |   12831.03   16514.36    -19536.53    45198.58
(nonsurvivor vs survivor) 10 |   360.4604   16234.15    -31457.88     32178.8
(nonsurvivor vs survivor) 11 |  -13779.49   16116.06    -45366.39    17807.41
(nonsurvivor vs survivor) 12 |  -29358.67   16174.56    -61060.23    2342.896
(nonsurvivor vs survivor) 13 |  -46083.82   16411.85    -78250.46   -13917.18
(nonsurvivor vs survivor) 14 |  -63608.35   16817.68     -96570.4   -30646.29
(nonsurvivor vs survivor) 15 |  -81545.08   17370.36    -115590.4    -47499.8
(nonsurvivor vs survivor) 16 |  -99481.31   18039.02    -134837.1   -64125.49
(nonsurvivor vs survivor) 17 |  -116995.3   18786.84    -153816.8   -80173.76
(nonsurvivor vs survivor) 18 |  -133673.3   19574.76    -172039.1   -95307.43
(nonsurvivor vs survivor) 19 |  -149125.8   20364.96    -189040.4   -109211.2
(nonsurvivor vs survivor) 20 |    -163003   21123.87      -204405     -121601
(nonsurvivor vs survivor) 21 |  -175007.3   21824.18    -217781.9   -132232.7
(nonsurvivor vs survivor) 22 |  -184903.4   22445.91    -228896.6   -140910.3
-----------------------------------------------------------------------------

199 . graph save "Graph" "/Users/Chiara/Documents/fileDO 15 Maggio/Marg_PLT.gph", replace
    (file /Users/Chiara/Documents/fileDO 15 Maggio/Marg_PLT.gph saved)

200 .
201 . *margins death_30, at(b_day_malattia=(0(1)30))  expression(exp(predict(xb)))
202 . *marginsplot, title("Platelets kinetic day 0 to day 30 after onset") ytitle("Cells per mmc x 1000") xlab(0(5)30) y
    > lab(0(50000)400000, angle(horizontal)) yline(150000,lcolor(red)) legend(off)
203 . *margins ar.death_30, at(b_day_malattia=(0(1)30))  expression(exp(predict(xb)))
204 .
205 . ***combine*****
206 . gr combine "/Users/Chiara/Documents/fileDO 15 Maggio/Marg_PLT.gph" "/Users/Chiara/Documents/fileDO 15 Maggio/BOX_P
    > LT.gph", ycomm   xsize(8) ysize(4)

207 . graph save "Graph" "/Users/Chiara/Documents/fileDO 15 Maggio/Graph_PLT.gph", replace
    (file /Users/Chiara/Documents/fileDO 15 Maggio/Graph_PLT.gph saved)

208 .
209 . ***************************************************************************
210 . *** analisi MPV  ***************************************************
211 . ***************************************************************************
212 . **** scatter no model
213 . use "/Users/Chiara/Documents/fileDO 15 Maggio/fileDO\coorte_long_emocromo_anemia_1.dta", clear

214 . *scatter mpv b_day_malattia  if death_30==1, mcolor(red) legend(off) title("MPV variation over time") xtitle("day
    > since onset") ytitle("Cell volume (fL)") xlab(0(5)30)|| scatter mpv b_day_malattia  if death_30==0, mcolor(green)
    > legend(off) ms(oh)
215 .
216 . *********Graph box**************************************
217 . graph box mpv, over(death_30, label(nolabel)) over(b_day_malattia, gap(5)) asyvars box(1, fcolor(navy)) nooutsides
    >  ytitle(Cells volume (fl)) legend(off)

218 . graph save "Graph" "/Users/Chiara/Documents/fileDO 15 Maggio/BOX_MPV.gph", replace
    (file /Users/Chiara/Documents/fileDO 15 Maggio/BOX_MPV.gph saved)

219 .
220 . *****modelling******
221 . use "/Users/Chiara/Documents/fileDO 15 Maggio/fileDO\coorte_long_emocromo_anemia_1.dta", clear

222 . mixed mpv_ln c.b_day_malattia##i.death_30 || progr:b_day_malattia, cov(unstr)

    Performing EM optimization:

    Performing gradient-based optimization:

    Iteration 0:   log likelihood =  2703.4127
    Iteration 1:   log likelihood =  2703.4129

    Computing standard errors:

    Mixed-effects ML regression                     Number of obs     =      1,773
    Group variable: progr                           Number of groups  =        376

                                                    Obs per group:
                                                                  min =          1
                                                                  avg =        4.7
                                                                  max =         22

                                                    Wald chi2(3)      =      98.30
    Log likelihood =  2703.4129                     Prob > chi2       =     0.0000

    ------------------------------------------------------------------------------------------
                      mpv_ln |      Coef.   Std. Err.      z    P>|z|     [95% Conf. Interval]
    -------------------------+----------------------------------------------------------------
              b_day_malattia |  -.0029786   .0004825    -6.17   0.000    -.0039243   -.0020328
                             |
                    death_30 |
                 nonsurvivor |   -.066807   .0203881    -3.28   0.001    -.1067669   -.0268471
                             |
    death_30#c.b_day_malattia |
                 nonsurvivor |   .0122826   .0014827     8.28   0.000     .0093766    .0151886
                             |
                       _cons |   2.402822   .0072018   333.64   0.000     2.388707    2.416938
    ------------------------------------------------------------------------------------------

    ------------------------------------------------------------------------------
      Random-effects Parameters  |   Estimate   Std. Err.     [95% Conf. Interval]
    -----------------------------+------------------------------------------------
    progr: Unstructured          |
             var(b_day_malattia) |   .0000422   4.78e-06      .0000338    .0000527
                       var(_cons) |   .0120543   .0011994      .0099185    .0146499
       cov(b_day_malattia,_cons) |  -.0004925   .0000673     -.0006244   -.0003606
    -----------------------------+------------------------------------------------
                   var(Residual) |   .0011794   .0000494      .0010863    .0012804
    ------------------------------------------------------------------------------
    LR test vs. linear model: chi2(3) = 1847.24               Prob > chi2 = 0.0000

    Note: LR test is conservative and provided only for reference.
```

```
223 . est store linear

224 . mixed mpv_ln  c.b_day_malattia##c.b_day_malattia##i.death_30 i.p_age2 i.p_cardio i.p_obeso i.p_rene|| progr:b_day_
    > malattia, cov(unstr)

    Performing EM optimization:

    Performing gradient-based optimization:

    Iteration 0:   log likelihood =  2721.9498
    Iteration 1:   log likelihood =  2721.9499

    Computing standard errors:

    Mixed-effects ML regression                     Number of obs     =      1,773
    Group variable: progr                           Number of groups  =        376

                                                    Obs per group:
                                                                  min =          1
                                                                  avg =        4.7
                                                                  max =         22

                                                    Wald chi2(9)      =     137.32
    Log likelihood =  2721.9499                     Prob > chi2       =     0.0000
```

mpv_ln	Coef.	Std. Err.	z	P>|z|	[95% Conf.	Interval]
b_day_malattia	-.0077241	.0011545	-6.69	0.000	-.0099869	-.0054612
c.b_day_malattia#c.b_day_malattia	.0001913	.0000427	4.48	0.000	.0001075	.0002751
death_30						
nonsurvivor	-.0830919	.0241874	-3.44	0.001	-.1304983	-.0356854
death_30#c.b_day_malattia						
nonsurvivor	.0115817	.002954	3.92	0.000	.0057919	.0173714
death_30#c.b_day_malattia#c.b_day_malattia						
nonsurvivor	.0000729	.0001181	0.62	0.537	-.0001586	.0003044
1.p_age2	.0148626	.009325	1.59	0.111	-.003414	.0331392
1.p_cardio	.0047159	.0099999	0.47	0.637	-.0148836	.0243154
1.p_obeso	-.014846	.017699	-0.84	0.402	-.0495354	.0198434
1.p_rene	.0475706	.0207101	2.30	0.022	.0069795	.0881618
_cons	2.418874	.010416	232.23	0.000	2.398459	2.439289

Random-effects Parameters	Estimate	Std. Err.	[95% Conf.	Interval]
progr: Unstructured				
var(b_day_malattia)	.0000431	4.86e-06	.0000346	.0000538
var(_cons)	.0122388	.0012204	.0100661	.0148804
cov(b_day_malattia,_cons)	-.0005139	.0000687	-.0006485	-.0003794
var(Residual)	.0011489	.0000484	.0010578	.0012477

```
    LR test vs. linear model: chi2(3) = 1816.08              Prob > chi2 = 0.0000

    Note: LR test is conservative and provided only for reference.

225 . est store quadratic

226 . lrtest linear quadratic

    Likelihood-ratio test                                 LR chi2(6)  =     37.07
    (Assumption: linear nested in quadratic)              Prob > chi2 =    0.0000

227 . ****margins*****
228 . quiet: mixed mpv_ln  c.b_day_malattia##c.b_day_malattia##i.death_30 i.p_age2 i.p_cardio i.p_obeso i.p_rene|| progr
    > :b_day_malattia, cov(unstr)

229 . margins death_30, at(b_day_malattia=(0(1)21))  expression(exp(predict(xb))) asbal

    Adjusted predictions                            Number of obs     =      1,773

    Expression   : exp(predict(xb))

    1._at        : b_day_mala~a    =           0
                   death_30                      (asbalanced)
                   p_age2                        (asbalanced)
                   p_cardio                      (asbalanced)
                   p_obeso                       (asbalanced)
                   p_rene                        (asbalanced)

    2._at        : b_day_mala~a    =           1
                   death_30                      (asbalanced)
                   p_age2                        (asbalanced)
                   p_cardio                      (asbalanced)
                   p_obeso                       (asbalanced)
                   p_rene                        (asbalanced)

    3._at        : b_day_mala~a    =           2
                   death_30                      (asbalanced)
                   p_age2                        (asbalanced)
                   p_cardio                      (asbalanced)
                   p_obeso                       (asbalanced)
                   p_rene                        (asbalanced)

    4._at        : b_day_mala~a    =           3
                   death_30                      (asbalanced)
                   p_age2                        (asbalanced)
                   p_cardio                      (asbalanced)
                   p_obeso                       (asbalanced)
                   p_rene                        (asbalanced)

    5._at        : b_day_mala~a    =           4
```

```
                death_30                     (asbalanced)
                p_age2                       (asbalanced)
                p_cardio                     (asbalanced)
                p_obeso                      (asbalanced)
                p_rene                       (asbalanced)

6._at         : b_day_mala~a    =          5
                death_30                     (asbalanced)
                p_age2                       (asbalanced)
                p_cardio                     (asbalanced)
                p_obeso                      (asbalanced)
                p_rene                       (asbalanced)

7._at         : b_day_mala~a    =          6
                death_30                     (asbalanced)
                p_age2                       (asbalanced)
                p_cardio                     (asbalanced)
                p_obeso                      (asbalanced)
                p_rene                       (asbalanced)

8._at         : b_day_mala~a    =          7
                death_30                     (asbalanced)
                p_age2                       (asbalanced)
                p_cardio                     (asbalanced)
                p_obeso                      (asbalanced)
                p_rene                       (asbalanced)

9._at         : b_day_mala~a    =          8
                death_30                     (asbalanced)
                p_age2                       (asbalanced)
                p_cardio                     (asbalanced)
                p_obeso                      (asbalanced)
                p_rene                       (asbalanced)

10._at        : b_day_mala~a    =          9
                death_30                     (asbalanced)
                p_age2                       (asbalanced)
                p_cardio                     (asbalanced)
                p_obeso                      (asbalanced)
                p_rene                       (asbalanced)

11._at        : b_day_mala~a    =         10
                death_30                     (asbalanced)
                p_age2                       (asbalanced)
                p_cardio                     (asbalanced)
                p_obeso                      (asbalanced)
                p_rene                       (asbalanced)

12._at        : b_day_mala~a    =         11
                death_30                     (asbalanced)
                p_age2                       (asbalanced)
                p_cardio                     (asbalanced)
                p_obeso                      (asbalanced)
                p_rene                       (asbalanced)

13._at        : b_day_mala~a    =         12
                death_30                     (asbalanced)
                p_age2                       (asbalanced)
                p_cardio                     (asbalanced)
                p_obeso                      (asbalanced)
                p_rene                       (asbalanced)

14._at        : b_day_mala~a    =         13
                death_30                     (asbalanced)
                p_age2                       (asbalanced)
                p_cardio                     (asbalanced)
                p_obeso                      (asbalanced)
                p_rene                       (asbalanced)

15._at        : b_day_mala~a    =         14
                death_30                     (asbalanced)
                p_age2                       (asbalanced)
                p_cardio                     (asbalanced)
                p_obeso                      (asbalanced)
                p_rene                       (asbalanced)

16._at        : b_day_mala~a    =         15
                death_30                     (asbalanced)
                p_age2                       (asbalanced)
                p_cardio                     (asbalanced)
                p_obeso                      (asbalanced)
                p_rene                       (asbalanced)

17._at        : b_day_mala~a    =         16
                death_30                     (asbalanced)
                p_age2                       (asbalanced)
                p_cardio                     (asbalanced)
                p_obeso                      (asbalanced)
                p_rene                       (asbalanced)

18._at        : b_day_mala~a    =         17
                death_30                     (asbalanced)
                p_age2                       (asbalanced)
                p_cardio                     (asbalanced)
                p_obeso                      (asbalanced)
                p_rene                       (asbalanced)

19._at        : b_day_mala~a    =         18
                death_30                     (asbalanced)
                p_age2                       (asbalanced)
                p_cardio                     (asbalanced)
                p_obeso                      (asbalanced)
                p_rene                       (asbalanced)

20._at        : b_day_mala~a    =         19
                death_30                     (asbalanced)
                p_age2                       (asbalanced)
                p_cardio                     (asbalanced)
```

```
              p_obeso                      (asbalanced)
              p_rene                       (asbalanced)

21._at       : b_day_mala~a    =         20
              death_30                     (asbalanced)
              p_age2                       (asbalanced)
              p_cardio                     (asbalanced)
              p_obeso                      (asbalanced)
              p_rene                       (asbalanced)

22._at       : b_day_mala~a    =         21
              death_30                     (asbalanced)
              p_age2                       (asbalanced)
              p_cardio                     (asbalanced)
              p_obeso                      (asbalanced)
              p_rene                       (asbalanced)
```

	Margin	Delta-method Std. Err.	z	P>|z|	[95% Conf.	Interval]
_at#death_30						
1#survivor	11.53085	.1826359	63.14	0.000	11.17289	11.88881
1#nonsurvivor	10.61145	.2487169	42.66	0.000	10.12398	11.09893
2#survivor	11.44432	.1754135	65.24	0.000	11.10051	11.78812
2#nonsurvivor	10.65528	.2318052	45.97	0.000	10.20095	11.10961
3#survivor	11.36278	.1693532	67.10	0.000	11.03085	11.69471
3#nonsurvivor	10.70495	.2176766	49.18	0.000	10.27831	11.13159
4#survivor	11.28614	.1643296	68.68	0.000	10.96406	11.60822
4#nonsurvivor	10.76053	.2062348	52.18	0.000	10.35631	11.16474
5#survivor	11.21431	.1602145	70.00	0.000	10.9003	11.52833
5#nonsurvivor	10.82211	.1973311	54.84	0.000	10.43535	11.20887
6#survivor	11.1472	.156881	71.06	0.000	10.83972	11.45468
6#nonsurvivor	10.8898	.1907661	57.08	0.000	10.51591	11.2637
7#survivor	11.08474	.1542083	71.88	0.000	10.78249	11.38698
7#nonsurvivor	10.96371	.1863015	58.85	0.000	10.59856	11.32885
8#survivor	11.02684	.1520849	72.50	0.000	10.72876	11.32492
8#nonsurvivor	11.04395	.1836821	60.13	0.000	10.68394	11.40396
9#survivor	10.97344	.150412	72.96	0.000	10.67864	11.26824
9#nonsurvivor	11.13065	.1826624	60.94	0.000	10.77264	11.48866
10#survivor	10.92448	.1491056	73.27	0.000	10.63224	11.21672
10#nonsurvivor	11.22397	.1830332	61.32	0.000	10.86523	11.58271
11#survivor	10.8799	.1480981	73.46	0.000	10.58963	11.17017
11#nonsurvivor	11.32405	.1846444	61.33	0.000	10.96215	11.68595
12#survivor	10.83965	.1473391	73.57	0.000	10.55087	11.12843
12#nonsurvivor	11.43106	.1874212	60.99	0.000	11.06372	11.7984
13#survivor	10.80368	.1467958	73.60	0.000	10.51597	11.0914
13#nonsurvivor	11.54518	.1913738	60.33	0.000	11.1701	11.92027
14#survivor	10.77195	.146453	73.55	0.000	10.48491	11.059
14#nonsurvivor	11.66661	.1966	59.34	0.000	11.28128	12.05193
15#survivor	10.74443	.1463129	73.43	0.000	10.45766	11.0312
15#nonsurvivor	11.79554	.2032828	58.03	0.000	11.39711	12.19396
16#survivor	10.72108	.146395	73.23	0.000	10.43415	11.00801
16#nonsurvivor	11.93219	.2116806	56.37	0.000	11.51731	12.34708
17#survivor	10.70187	.1467354	72.93	0.000	10.41427	10.98947
17#nonsurvivor	12.07682	.2221124	54.37	0.000	11.64148	12.51215
18#survivor	10.68678	.1473858	72.51	0.000	10.39791	10.97566
18#nonsurvivor	12.22965	.2349381	52.05	0.000	11.76918	12.69012
19#survivor	10.67581	.1484131	71.93	0.000	10.38492	10.96669
19#nonsurvivor	12.39096	.2505369	49.46	0.000	11.89992	12.88201
20#survivor	10.66892	.1498972	71.17	0.000	10.37513	10.96271
20#nonsurvivor	12.56104	.2692868	46.65	0.000	12.03325	13.08883
21#survivor	10.66612	.1519289	70.20	0.000	10.36834	10.96389
21#nonsurvivor	12.74018	.2915489	43.70	0.000	12.16876	13.31161
22#survivor	10.6674	.1546074	69.00	0.000	10.36437	10.97042
22#nonsurvivor	12.92871	.317658	40.70	0.000	12.30611	13.55131

```
230 . marginsplot, ytitle("Cell volume (fL)")xlab(0(1)21) ylab(8(2)14) legend(off)

      Variables that uniquely identify margins: b_day_malattia death_30

231 . margins ar.death_30, at(b_day_malattia=(0(1)21))  expression(exp(predict(xb))) asbal

    Contrasts of adjusted predictions              Number of obs     =      1,773

    Expression   : exp(predict(xb))

    1._at        : b_day_mala~a    =          0
                   death_30                     (asbalanced)
                   p_age2                       (asbalanced)
                   p_cardio                     (asbalanced)
                   p_obeso                      (asbalanced)
                   p_rene                       (asbalanced)

    2._at        : b_day_mala~a    =          1
                   death_30                     (asbalanced)
                   p_age2                       (asbalanced)
                   p_cardio                     (asbalanced)
                   p_obeso                      (asbalanced)
                   p_rene                       (asbalanced)

    3._at        : b_day_mala~a    =          2
                   death_30                     (asbalanced)
                   p_age2                       (asbalanced)
                   p_cardio                     (asbalanced)
                   p_obeso                      (asbalanced)
                   p_rene                       (asbalanced)

    4._at        : b_day_mala~a    =          3
                   death_30                     (asbalanced)
                   p_age2                       (asbalanced)
                   p_cardio                     (asbalanced)
                   p_obeso                      (asbalanced)
                   p_rene                       (asbalanced)

    5._at        : b_day_mala~a    =          4
                   death_30                     (asbalanced)
```

```
                p_age2                        (asbalanced)
                p_cardio                      (asbalanced)
                p_obeso                       (asbalanced)
                p_rene                        (asbalanced)

6._at         : b_day_mala~a    =          5
                death_30                      (asbalanced)
                p_age2                        (asbalanced)
                p_cardio                      (asbalanced)
                p_obeso                       (asbalanced)
                p_rene                        (asbalanced)

7._at         : b_day_mala~a    =          6
                death_30                      (asbalanced)
                p_age2                        (asbalanced)
                p_cardio                      (asbalanced)
                p_obeso                       (asbalanced)
                p_rene                        (asbalanced)

8._at         : b_day_mala~a    =          7
                death_30                      (asbalanced)
                p_age2                        (asbalanced)
                p_cardio                      (asbalanced)
                p_obeso                       (asbalanced)
                p_rene                        (asbalanced)

9._at         : b_day_mala~a    =          8
                death_30                      (asbalanced)
                p_age2                        (asbalanced)
                p_cardio                      (asbalanced)
                p_obeso                       (asbalanced)
                p_rene                        (asbalanced)

10._at        : b_day_mala~a    =          9
                death_30                      (asbalanced)
                p_age2                        (asbalanced)
                p_cardio                      (asbalanced)
                p_obeso                       (asbalanced)
                p_rene                        (asbalanced)

11._at        : b_day_mala~a    =         10
                death_30                      (asbalanced)
                p_age2                        (asbalanced)
                p_cardio                      (asbalanced)
                p_obeso                       (asbalanced)
                p_rene                        (asbalanced)

12._at        : b_day_mala~a    =         11
                death_30                      (asbalanced)
                p_age2                        (asbalanced)
                p_cardio                      (asbalanced)
                p_obeso                       (asbalanced)
                p_rene                        (asbalanced)

13._at        : b_day_mala~a    =         12
                death_30                      (asbalanced)
                p_age2                        (asbalanced)
                p_cardio                      (asbalanced)
                p_obeso                       (asbalanced)
                p_rene                        (asbalanced)

14._at        : b_day_mala~a    =         13
                death_30                      (asbalanced)
                p_age2                        (asbalanced)
                p_cardio                      (asbalanced)
                p_obeso                       (asbalanced)
                p_rene                        (asbalanced)

15._at        : b_day_mala~a    =         14
                death_30                      (asbalanced)
                p_age2                        (asbalanced)
                p_cardio                      (asbalanced)
                p_obeso                       (asbalanced)
                p_rene                        (asbalanced)

16._at        : b_day_mala~a    =         15
                death_30                      (asbalanced)
                p_age2                        (asbalanced)
                p_cardio                      (asbalanced)
                p_obeso                       (asbalanced)
                p_rene                        (asbalanced)

17._at        : b_day_mala~a    =         16
                death_30                      (asbalanced)
                p_age2                        (asbalanced)
                p_cardio                      (asbalanced)
                p_obeso                       (asbalanced)
                p_rene                        (asbalanced)

18._at        : b_day_mala~a    =         17
                death_30                      (asbalanced)
                p_age2                        (asbalanced)
                p_cardio                      (asbalanced)
                p_obeso                       (asbalanced)
                p_rene                        (asbalanced)

19._at        : b_day_mala~a    =         18
                death_30                      (asbalanced)
                p_age2                        (asbalanced)
                p_cardio                      (asbalanced)
                p_obeso                       (asbalanced)
                p_rene                        (asbalanced)

20._at        : b_day_mala~a    =         19
                death_30                      (asbalanced)
                p_age2                        (asbalanced)
                p_cardio                      (asbalanced)
                p_obeso                       (asbalanced)
```

```
                p_rene                        (asbalanced)

     21._at       : b_day_mala~a    =          20
                    death_30                   (asbalanced)
                    p_age2                     (asbalanced)
                    p_cardio                   (asbalanced)
                    p_obeso                    (asbalanced)
                    p_rene                     (asbalanced)

     22._at       : b_day_mala~a    =          21
                    death_30                   (asbalanced)
                    p_age2                     (asbalanced)
                    p_cardio                   (asbalanced)
                    p_obeso                    (asbalanced)
                    p_rene                     (asbalanced)
```

	df	chi2	P>chi2
death_30@_at			
(nonsurvivor vs survivor) 1	1	12.27	0.0005
(nonsurvivor vs survivor) 2	1	10.69	0.0011
(nonsurvivor vs survivor) 3	1	8.68	0.0032
(nonsurvivor vs survivor) 4	1	6.37	0.0116
(nonsurvivor vs survivor) 5	1	4.01	0.0453
(nonsurvivor vs survivor) 6	1	1.91	0.1674
(nonsurvivor vs survivor) 7	1	0.46	0.4998
(nonsurvivor vs survivor) 8	1	0.01	0.9219
(nonsurvivor vs survivor) 9	1	0.84	0.3597
(nonsurvivor vs survivor) 10	1	3.09	0.0789
(nonsurvivor vs survivor) 11	1	6.76	0.0093
(nonsurvivor vs survivor) 12	1	11.75	0.0006
(nonsurvivor vs survivor) 13	1	17.81	0.0000
(nonsurvivor vs survivor) 14	1	24.60	0.0000
(nonsurvivor vs survivor) 15	1	31.68	0.0000
(nonsurvivor vs survivor) 16	1	38.54	0.0000
(nonsurvivor vs survivor) 17	1	44.67	0.0000
(nonsurvivor vs survivor) 18	1	49.62	0.0000
(nonsurvivor vs survivor) 19	1	53.08	0.0000
(nonsurvivor vs survivor) 20	1	54.96	0.0000
(nonsurvivor vs survivor) 21	1	55.34	0.0000
(nonsurvivor vs survivor) 22	1	54.45	0.0000
Joint	5	100.00	0.0000

	Contrast	Delta-method Std. Err.	[95% Conf.	Interval]
death_30@_at				
(nonsurvivor vs survivor) 1	-.9193936	.2624571	-1.4338	-.404987
(nonsurvivor vs survivor) 2	-.7890325	.2413354	-1.262041	-.3160238
(nonsurvivor vs survivor) 3	-.657832	.2232637	-1.095421	-.2202431
(nonsurvivor vs survivor) 4	-.5256145	.2081744	-.9336287	-.1176002
(nonsurvivor vs survivor) 5	-.3921989	.1959516	-.776257	-.0081408
(nonsurvivor vs survivor) 6	-.2574002	.1864246	-.6227857	.1079853
(nonsurvivor vs survivor) 7	-.1210289	.1793709	-.4725895	.2305317
(nonsurvivor vs survivor) 8	.0171092	.1745328	-.3249688	.3591872
(nonsurvivor vs survivor) 9	.1572137	.171644	-.1792024	.4936297
(nonsurvivor vs survivor) 10	.2994899	.1704628	-.034611	.6335908
(nonsurvivor vs survivor) 11	.4441495	.1708031	.1093817	.7789173
(nonsurvivor vs survivor) 12	.591411	.1725596	.2532004	.9296216
(nonsurvivor vs survivor) 13	.7415003	.1757239	.3970878	1.085913
(nonsurvivor vs survivor) 14	.8946513	.1803898	.5410939	1.248209
(nonsurvivor vs survivor) 15	1.051107	.1867492	.6850849	1.417128
(nonsurvivor vs survivor) 16	1.211118	.1950784	.8287711	1.593464
(nonsurvivor vs survivor) 17	1.374947	.2057162	.9717504	1.778143
(nonsurvivor vs survivor) 18	1.542866	.2190364	1.113562	1.972169
(nonsurvivor vs survivor) 19	1.715159	.2354206	1.253743	2.176575
(nonsurvivor vs survivor) 20	1.892122	.2552347	1.391872	2.392373
(nonsurvivor vs survivor) 21	2.074065	.2788147	1.527598	2.620532
(nonsurvivor vs survivor) 22	2.26131	.3064621	1.660656	2.861965

```
232 . graph save "Graph" "/Users/Chiara/Documents/fileDO 15 Maggio/Marg_MPV.gph", replace
    (file /Users/Chiara/Documents/fileDO 15 Maggio/Marg_MPV.gph saved)

233 .
234 . *margins death_30, at(b_day_malattia=(0(1)30))  expression(exp(predict(xb)))
235 . *marginsplot, title("MPV kinetic day 0 to day 30 after onset") ytitle("Cell volume (fl)")xlab(0(5)30) ylab(5(2)15,
    >  angle(horizontal)) yline(7, lcolor(red)) yline(11, lcolor(green)) legend(off)
236 . *margins ar.death_30, at(b_day_malattia=(0(1)30))  expression(exp(predict(xb)))
237 .
238 . ***combine*****
239 . gr combine "/Users/Chiara/Documents/fileDO 15 Maggio/Marg_MPV.gph" "/Users/Chiara/Documents/fileDO 15 Maggio/BOX_M
    > PV.gph", ycomm   xsize(8) ysize(4)

240 . graph save "Graph" "/Users/Chiara/Documents/fileDO 15 Maggio/Graph_MPV.gph", replace
    (file /Users/Chiara/Documents/fileDO 15 Maggio/Graph_MPV.gph saved)

241 .
242 .
243 . ***************************************************************************
244 . *** analisi HCT  **************************************************
245 . ***************************************************************************
246 . **** scatter no model
247 . *use "/Users/Chiara/Documents/fileDO 15 Maggio/fileDO\coorte_long_emocromo_anemia_1.dta", clear
248 . *scatter hct b_day_malattia  if death_30==1, mcolor(red) legend(off) title("HCT %") xtitle("day since onset") ytit
    > le("HCT %") || scatter hct b_day_malattia  if death_30==0, mcolor(green) legend(off) ms(oh)
249 .
250 . **** modelling
251 . *use "/Users/Chiara/Documents/fileDO 15 Maggio/fileDO\coorte_long_emocromo_anemia_1.dta", clear
252 . *mixed hct_ln c.b_day_malattia##i.death_30 || progr:b_day_malattia, cov(unstr)
253 . *est store linear
254 . *mixed hct_ln  c.b_day_malattia##c.b_day_malattia##i.death_30 || progr:b_day_malattia, cov(unstr)
255 . *est store quadratic
256 . *lrtest linear quadratic
257 .
```

```
258 . *margins death_30, at(b_day_malattia=(0(1)30))  expression(exp(predict(xb)))
259 . *marginsplot, title("HCT kinetic day 0 to day 28 after onset") ytitle("Cell volume (fL; log-scale)")
260 . *margins ar.death_30, at(b_day_malattia=(0(1)30))  expression(exp(predict(xb)))
261 .
262 .
263 . ***********************************************************************
264 . *** analisi mcv  ****************************************************
265 . ***********************************************************************
266 . **** scatter no model
267 . use "/Users/Chiara/Documents/fileDO 15 Maggio/fileDO\coorte_long_emocromo_anemia_1.dta", clear

268 . *scatter mcv b_day_malattia  if death_30==1, mcolor(red) legend(off) title("MCV variation over time") xtitle("day
    > since onset") ytitle("Cell volume (fL)") xlab(0(5)30)|| scatter mcv b_day_malattia  if death_30==0, mcolor(green)
    > legend(off) ms(oh)
269 .
270 . ********Graph box**************************************
271 . graph box mcv, over(death_30, label(nolabel)) over(b_day_malattia) asyvars box(1, fcolor(navy))  nooutsides ytitle
    > (Cells volume (fl)) yline(80, lcolor(red)) legend(off)

272 . graph save "Graph" "/Users/Chiara/Documents/fileDO 15 Maggio/BOX_MCV.gph", replace
    (file /Users/Chiara/Documents/fileDO 15 Maggio/BOX_MCV.gph saved)

273 . **** modelling
274 . use "/Users/Chiara/Documents/fileDO 15 Maggio/fileDO\coorte_long_emocromo_anemia_1.dta", clear

275 . mixed mcv_ln c.b_day_malattia##i.death_30 || progr:b_day_malattia, cov(unstr)

    Performing EM optimization:

    Performing gradient-based optimization:

    Iteration 0:   log likelihood =  3881.4055
    Iteration 1:   log likelihood =  3881.4181
    Iteration 2:   log likelihood =  3881.4181

    Computing standard errors:

    Mixed-effects ML regression                     Number of obs     =      1,805
    Group variable: progr                           Number of groups  =        379

                                                    Obs per group:
                                                                  min =          1
                                                                  avg =        4.8
                                                                  max =         22

                                                    Wald chi2(3)      =      42.56
    Log likelihood =  3881.4181                     Prob > chi2       =     0.0000
```

mcv_ln	Coef.	Std. Err.	z	P>|z|	[95% Conf.	Interval]
b_day_malattia	.0010319	.0002037	5.07	0.000	.0006326	.0014312
death_30						
nonsurvivor	.0508694	.0124139	4.10	0.000	.0265387	.0752001
death_30#c.b_day_malattia						
nonsurvivor	-.0004992	.0006146	-0.81	0.417	-.0017039	.0007054
_cons	4.46217	.004274	1044.02	0.000	4.453793	4.470547

Random-effects Parameters	Estimate	Std. Err.	[95% Conf.	Interval]
**progr:** Unstructured				
var(b_day_malattia)	5.95e-06	8.62e-07	4.48e-06	7.90e-06
var(_cons)	.0049492	.0004213	.0041887	.0058477
cov(b_day_malattia,_cons)	-.000066	.0000146	-.0000946	-.0000373
var(Residual)	.0002965	.0000122	.0002735	.0003213

```
    LR test vs. linear model: chi2(3) = 3647.86              Prob > chi2 = 0.0000

    Note: LR test is conservative and provided only for reference.

276 . est store linear

277 . mixed mcv_ln  c.b_day_malattia##c.b_day_malattia##i.death_30 i.p_age2 i.p_cardio i.p_obeso i.p_rene|| progr:b_day_
    > malattia, cov(unstr)

    Performing EM optimization:

    Performing gradient-based optimization:

    Iteration 0:   log likelihood =  3895.8695
    Iteration 1:   log likelihood =  3895.8837
    Iteration 2:   log likelihood =  3895.8837

    Computing standard errors:

    Mixed-effects ML regression                     Number of obs     =      1,805
    Group variable: progr                           Number of groups  =        379

                                                    Obs per group:
                                                                  min =          1
                                                                  avg =        4.8
                                                                  max =         22

                                                    Wald chi2(9)      =      71.94
    Log likelihood =  3895.8837                     Prob > chi2       =     0.0000
```

mcv_ln	Coef.	Std. Err.	z	P>|z|	[95% Conf.	Interval]
b_day_malattia	-.0001139	.0005479	-0.21	0.835	-.0011878	.00096
c.b_day_malattia#c.b_day_malattia	.0000462	.0000206	2.25	0.025	5.93e-06	.0000865

```
                                     death_30 |
                                  nonsurvivor |   .0263266   .0142429     1.85   0.065     -.001589    .0542422
                                              |
                    death_30#c.b_day_malattia |
                                  nonsurvivor |   .0046663   .0014023     3.33   0.001     .0019179    .0074147
                                              |
death_30#c.b_day_malattia#c.b_day_malattia |
                                  nonsurvivor |  -.0002287   .0000565    -4.05   0.000    -.0003394    -.000118
                                              |
                                     1.p_age2 |   .0119403   .0072937     1.64   0.102    -.0023551    .0262357
                                   1.p_cardio |  -.0194012   .0078147    -2.48   0.013    -.0347176   -.0040847
                                    1.p_obeso |  -.0174175   .0139158    -1.25   0.211    -.0446919     .009857
                                     1.p_rene |   .0361034   .0162436     2.22   0.026     .0042665    .0679404
                                        _cons |   4.467564   .0063685   701.51   0.000     4.455082    4.480046
------------------------------------------------------------------------------------------------------------

------------------------------------------------------------------------------
  Random-effects Parameters  |   Estimate   Std. Err.     [95% Conf. Interval]
-----------------------------+------------------------------------------------
progr: Unstructured          |
        var(b_day_malattia)  |   6.05e-06   8.73e-07      4.56e-06    8.03e-06
                  var(_cons) |   .0048844   .0004193      .0041281    .0057794
   cov(b_day_malattia,_cons) |  -.0000698   .0000149      -.000099   -.0000406
-----------------------------+------------------------------------------------
               var(Residual) |    .000292    .000012      .0002693    .0003165
------------------------------------------------------------------------------
LR test vs. linear model: chi2(3) = 3601.78               Prob > chi2 = 0.0000

Note: LR test is conservative and provided only for reference.

278 . est store quadratic

279 . lrtest linear quadratic

Likelihood-ratio test                                 LR chi2(6)  =     28.93
(Assumption: linear nested in quadratic)              Prob > chi2 =    0.0001

280 . ****margins*********
281 . quiet: mixed mcv_ln  c.b_day_malattia##c.b_day_malattia##i.death_30 i.p_age2 i.p_cardio i.p_obeso i.p_rene|| progr
> :b_day_malattia, cov(unstr)

282 . margins death_30, at(b_day_malattia=(0(1)21))  expression(exp(predict(xb))) asbal

Adjusted predictions                            Number of obs     =      1,805

Expression   : exp(predict(xb))

1._at        : b_day_mala~a    =           0
               death_30                      (asbalanced)
               p_age2                        (asbalanced)
               p_cardio                      (asbalanced)
               p_obeso                       (asbalanced)
               p_rene                        (asbalanced)

2._at        : b_day_mala~a    =           1
               death_30                      (asbalanced)
               p_age2                        (asbalanced)
               p_cardio                      (asbalanced)
               p_obeso                       (asbalanced)
               p_rene                        (asbalanced)

3._at        : b_day_mala~a    =           2
               death_30                      (asbalanced)
               p_age2                        (asbalanced)
               p_cardio                      (asbalanced)
               p_obeso                       (asbalanced)
               p_rene                        (asbalanced)

4._at        : b_day_mala~a    =           3
               death_30                      (asbalanced)
               p_age2                        (asbalanced)
               p_cardio                      (asbalanced)
               p_obeso                       (asbalanced)
               p_rene                        (asbalanced)

5._at        : b_day_mala~a    =           4
               death_30                      (asbalanced)
               p_age2                        (asbalanced)
               p_cardio                      (asbalanced)
               p_obeso                       (asbalanced)
               p_rene                        (asbalanced)

6._at        : b_day_mala~a    =           5
               death_30                      (asbalanced)
               p_age2                        (asbalanced)
               p_cardio                      (asbalanced)
               p_obeso                       (asbalanced)
               p_rene                        (asbalanced)

7._at        : b_day_mala~a    =           6
               death_30                      (asbalanced)
               p_age2                        (asbalanced)
               p_cardio                      (asbalanced)
               p_obeso                       (asbalanced)
               p_rene                        (asbalanced)

8._at        : b_day_mala~a    =           7
               death_30                      (asbalanced)
               p_age2                        (asbalanced)
               p_cardio                      (asbalanced)
               p_obeso                       (asbalanced)
               p_rene                        (asbalanced)

9._at        : b_day_mala~a    =           8
               death_30                      (asbalanced)
               p_age2                        (asbalanced)
               p_cardio                      (asbalanced)
```

```
                 p_obeso                        (asbalanced)
                 p_rene                         (asbalanced)

10._at         : b_day_mala~a    =           9
                 death_30                       (asbalanced)
                 p_age2                         (asbalanced)
                 p_cardio                       (asbalanced)
                 p_obeso                        (asbalanced)
                 p_rene                         (asbalanced)

11._at         : b_day_mala~a    =          10
                 death_30                       (asbalanced)
                 p_age2                         (asbalanced)
                 p_cardio                       (asbalanced)
                 p_obeso                        (asbalanced)
                 p_rene                         (asbalanced)

12._at         : b_day_mala~a    =          11
                 death_30                       (asbalanced)
                 p_age2                         (asbalanced)
                 p_cardio                       (asbalanced)
                 p_obeso                        (asbalanced)
                 p_rene                         (asbalanced)

13._at         : b_day_mala~a    =          12
                 death_30                       (asbalanced)
                 p_age2                         (asbalanced)
                 p_cardio                       (asbalanced)
                 p_obeso                        (asbalanced)
                 p_rene                         (asbalanced)

14._at         : b_day_mala~a    =          13
                 death_30                       (asbalanced)
                 p_age2                         (asbalanced)
                 p_cardio                       (asbalanced)
                 p_obeso                        (asbalanced)
                 p_rene                         (asbalanced)

15._at         : b_day_mala~a    =          14
                 death_30                       (asbalanced)
                 p_age2                         (asbalanced)
                 p_cardio                       (asbalanced)
                 p_obeso                        (asbalanced)
                 p_rene                         (asbalanced)

16._at         : b_day_mala~a    =          15
                 death_30                       (asbalanced)
                 p_age2                         (asbalanced)
                 p_cardio                       (asbalanced)
                 p_obeso                        (asbalanced)
                 p_rene                         (asbalanced)

17._at         : b_day_mala~a    =          16
                 death_30                       (asbalanced)
                 p_age2                         (asbalanced)
                 p_cardio                       (asbalanced)
                 p_obeso                        (asbalanced)
                 p_rene                         (asbalanced)

18._at         : b_day_mala~a    =          17
                 death_30                       (asbalanced)
                 p_age2                         (asbalanced)
                 p_cardio                       (asbalanced)
                 p_obeso                        (asbalanced)
                 p_rene                         (asbalanced)

19._at         : b_day_mala~a    =          18
                 death_30                       (asbalanced)
                 p_age2                         (asbalanced)
                 p_cardio                       (asbalanced)
                 p_obeso                        (asbalanced)
                 p_rene                         (asbalanced)

20._at         : b_day_mala~a    =          19
                 death_30                       (asbalanced)
                 p_age2                         (asbalanced)
                 p_cardio                       (asbalanced)
                 p_obeso                        (asbalanced)
                 p_rene                         (asbalanced)

21._at         : b_day_mala~a    =          20
                 death_30                       (asbalanced)
                 p_age2                         (asbalanced)
                 p_cardio                       (asbalanced)
                 p_obeso                        (asbalanced)
                 p_rene                         (asbalanced)

22._at         : b_day_mala~a    =          21
                 death_30                       (asbalanced)
                 p_age2                         (asbalanced)
                 p_cardio                       (asbalanced)
                 p_obeso                        (asbalanced)
                 p_rene                         (asbalanced)
```

	Margin	Delta-method Std. Err.	z	P>|z|	[95% Conf.	Interval]
_at#death_30						
1#survivor	87.63465	.9886045	88.64	0.000	85.69702	89.57228
1#nonsurvivor	89.97241	1.302937	69.05	0.000	87.4187	92.52612
2#survivor	87.62872	.9752644	89.85	0.000	85.71723	89.5402
2#nonsurvivor	90.36644	1.259146	71.77	0.000	87.89856	92.83432
3#survivor	87.63088	.9646516	90.84	0.000	85.7402	89.52156
3#nonsurvivor	90.72909	1.224892	74.07	0.000	88.32834	93.12983
4#survivor	87.64115	.9564088	91.64	0.000	85.76662	89.51567
4#nonsurvivor	91.05995	1.199193	75.93	0.000	88.70957	93.41032
5#survivor	87.65952	.9501902	92.25	0.000	85.79718	89.52185

```
     5#nonsurvivor |   91.35867    1.18091    77.36   0.000     89.04413    93.67321
        6#survivor |   87.68599   .9456694    92.72   0.000     85.83252    89.53947
     6#nonsurvivor |   91.62493   1.168824    78.39   0.000     89.33408    93.91578
        7#survivor |   87.72059   .9425454    93.07   0.000     85.87323    89.56794
     7#nonsurvivor |   91.85844   1.161722    79.07   0.000      89.5815    94.13537
        8#survivor |    87.7633   .9405486    93.31   0.000     85.91986    89.60674
     8#nonsurvivor |   92.05894   1.158473    79.47   0.000     89.78837     94.3295
        9#survivor |   87.81416   .9394438    93.47   0.000     85.97288    89.65543
     9#nonsurvivor |   92.22622   1.158087    79.64   0.000     89.95641    94.49603
       10#survivor |   87.87316   .9390334    93.58   0.000     86.03269    89.71363
    10#nonsurvivor |   92.36009    1.15976    79.64   0.000       90.087    94.63318
       11#survivor |   87.94033   .9391591    93.64   0.000     86.09961    89.78105
    11#nonsurvivor |    92.4604   1.162902    79.51   0.000     90.18116    94.73965
       12#survivor |   88.01569   .9397027    93.66   0.000     86.17391    89.85747
    12#nonsurvivor |   92.52706   1.167151    79.28   0.000     90.23949    94.81463
       13#survivor |   88.09925   .9405874    93.66   0.000     86.25574    89.94277
    13#nonsurvivor |   92.55998   1.172382    78.95   0.000     90.26215     94.8578
       14#survivor |   88.19105   .9417777    93.64   0.000      86.3452     90.0369
    14#nonsurvivor |   92.55913   1.178705    78.53   0.000     90.24891    94.86934
       15#survivor |    88.2911     .94328    93.60   0.000      86.4423    90.13989
    15#nonsurvivor |    92.5245   1.186459    77.98   0.000     90.19909    94.84992
       16#survivor |   88.39943   .9451424    93.53   0.000     86.54699    90.25188
    16#nonsurvivor |   92.45615   1.196199    77.29   0.000     90.11164    94.80065
       17#survivor |   88.51608   .9474552    93.43   0.000      86.6591    90.37306
    17#nonsurvivor |   92.35413   1.208677    76.41   0.000     89.98517    94.72309
       18#survivor |   88.64107   .9503504    93.27   0.000     86.77842    90.50373
    18#nonsurvivor |   92.21857   1.224809    75.29   0.000     89.81799    94.61915
       19#survivor |   88.77445   .9540015    93.05   0.000     86.90464    90.64426
    19#nonsurvivor |   92.04961   1.245635    73.90   0.000     89.60821    94.49101
       20#survivor |   88.91624   .9586224    92.75   0.000     87.03738    90.79511
    20#nonsurvivor |   91.84743   1.272264    72.19   0.000     89.35384    94.34103
       21#survivor |   89.06649   .9644658    92.35   0.000     87.17618    90.95681
    21#nonsurvivor |   91.61227    1.30581    70.16   0.000     89.05292    94.17161
       22#survivor |   89.22525   .9718204    91.81   0.000     87.32051    91.12998
    22#nonsurvivor |   91.34436   1.347326    67.80   0.000     88.70365    93.98507
--------------------------------------------------------------------------------

283 . marginsplot, ytitle("Cells volume (fl)") xlab(0(1)21) ylab(70(10)110) yline(80, lcolor(red)) legend(off)

      Variables that uniquely identify margins: b_day_malattia death_30

284 . margins ar.death_30, at(b_day_malattia=(0(1)21))  expression(exp(predict(xb))) asbal

    Contrasts of adjusted predictions              Number of obs     =      1,805

    Expression   : exp(predict(xb))

    1._at        : b_day_mala~a    =           0
                   death_30                      (asbalanced)
                   p_age2                        (asbalanced)
                   p_cardio                      (asbalanced)
                   p_obeso                       (asbalanced)
                   p_rene                        (asbalanced)

    2._at        : b_day_mala~a    =           1
                   death_30                      (asbalanced)
                   p_age2                        (asbalanced)
                   p_cardio                      (asbalanced)
                   p_obeso                       (asbalanced)
                   p_rene                        (asbalanced)

    3._at        : b_day_mala~a    =           2
                   death_30                      (asbalanced)
                   p_age2                        (asbalanced)
                   p_cardio                      (asbalanced)
                   p_obeso                       (asbalanced)
                   p_rene                        (asbalanced)

    4._at        : b_day_mala~a    =           3
                   death_30                      (asbalanced)
                   p_age2                        (asbalanced)
                   p_cardio                      (asbalanced)
                   p_obeso                       (asbalanced)
                   p_rene                        (asbalanced)

    5._at        : b_day_mala~a    =           4
                   death_30                      (asbalanced)
                   p_age2                        (asbalanced)
                   p_cardio                      (asbalanced)
                   p_obeso                       (asbalanced)
                   p_rene                        (asbalanced)

    6._at        : b_day_mala~a    =           5
                   death_30                      (asbalanced)
                   p_age2                        (asbalanced)
                   p_cardio                      (asbalanced)
                   p_obeso                       (asbalanced)
                   p_rene                        (asbalanced)

    7._at        : b_day_mala~a    =           6
                   death_30                      (asbalanced)
                   p_age2                        (asbalanced)
                   p_cardio                      (asbalanced)
                   p_obeso                       (asbalanced)
                   p_rene                        (asbalanced)

    8._at        : b_day_mala~a    =           7
                   death_30                      (asbalanced)
                   p_age2                        (asbalanced)
                   p_cardio                      (asbalanced)
                   p_obeso                       (asbalanced)
                   p_rene                        (asbalanced)

    9._at        : b_day_mala~a    =           8
                   death_30                      (asbalanced)
                   p_age2                        (asbalanced)
                   p_cardio                      (asbalanced)
                   p_obeso                       (asbalanced)
```

```
                 p_rene                        (asbalanced)

10._at       : b_day_mala~a    =          9
               death_30                        (asbalanced)
               p_age2                          (asbalanced)
               p_cardio                        (asbalanced)
               p_obeso                         (asbalanced)
               p_rene                          (asbalanced)

11._at       : b_day_mala~a    =         10
               death_30                        (asbalanced)
               p_age2                          (asbalanced)
               p_cardio                        (asbalanced)
               p_obeso                         (asbalanced)
               p_rene                          (asbalanced)

12._at       : b_day_mala~a    =         11
               death_30                        (asbalanced)
               p_age2                          (asbalanced)
               p_cardio                        (asbalanced)
               p_obeso                         (asbalanced)
               p_rene                          (asbalanced)

13._at       : b_day_mala~a    =         12
               death_30                        (asbalanced)
               p_age2                          (asbalanced)
               p_cardio                        (asbalanced)
               p_obeso                         (asbalanced)
               p_rene                          (asbalanced)

14._at       : b_day_mala~a    =         13
               death_30                        (asbalanced)
               p_age2                          (asbalanced)
               p_cardio                        (asbalanced)
               p_obeso                         (asbalanced)
               p_rene                          (asbalanced)

15._at       : b_day_mala~a    =         14
               death_30                        (asbalanced)
               p_age2                          (asbalanced)
               p_cardio                        (asbalanced)
               p_obeso                         (asbalanced)
               p_rene                          (asbalanced)

16._at       : b_day_mala~a    =         15
               death_30                        (asbalanced)
               p_age2                          (asbalanced)
               p_cardio                        (asbalanced)
               p_obeso                         (asbalanced)
               p_rene                          (asbalanced)

17._at       : b_day_mala~a    =         16
               death_30                        (asbalanced)
               p_age2                          (asbalanced)
               p_cardio                        (asbalanced)
               p_obeso                         (asbalanced)
               p_rene                          (asbalanced)

18._at       : b_day_mala~a    =         17
               death_30                        (asbalanced)
               p_age2                          (asbalanced)
               p_cardio                        (asbalanced)
               p_obeso                         (asbalanced)
               p_rene                          (asbalanced)

19._at       : b_day_mala~a    =         18
               death_30                        (asbalanced)
               p_age2                          (asbalanced)
               p_cardio                        (asbalanced)
               p_obeso                         (asbalanced)
               p_rene                          (asbalanced)

20._at       : b_day_mala~a    =         19
               death_30                        (asbalanced)
               p_age2                          (asbalanced)
               p_cardio                        (asbalanced)
               p_obeso                         (asbalanced)
               p_rene                          (asbalanced)

21._at       : b_day_mala~a    =         20
               death_30                        (asbalanced)
               p_age2                          (asbalanced)
               p_cardio                        (asbalanced)
               p_obeso                         (asbalanced)
               p_rene                          (asbalanced)

22._at       : b_day_mala~a    =         21
               death_30                        (asbalanced)
               p_age2                          (asbalanced)
               p_cardio                        (asbalanced)
               p_obeso                         (asbalanced)
               p_rene                          (asbalanced)
```

	df	chi2	P>chi2
death_30@_at			
(nonsurvivor vs survivor) 1	1	3.38	0.0660
(nonsurvivor vs survivor) 2	1	5.08	0.0241
(nonsurvivor vs survivor) 3	1	7.04	0.0080
(nonsurvivor vs survivor) 4	1	9.12	0.0025
(nonsurvivor vs survivor) 5	1	11.20	0.0008
(nonsurvivor vs survivor) 6	1	13.13	0.0003
(nonsurvivor vs survivor) 7	1	14.82	0.0001
(nonsurvivor vs survivor) 8	1	16.17	0.0001
(nonsurvivor vs survivor) 9	1	17.15	0.0000
(nonsurvivor vs survivor) 10	1	17.73	0.0000
(nonsurvivor vs survivor) 11	1	17.91	0.0000

(nonsurvivor vs survivor) 12	1	17.70	0.0000
(nonsurvivor vs survivor) 13	1	17.13	0.0000
(nonsurvivor vs survivor) 14	1	16.21	0.0001
(nonsurvivor vs survivor) 15	1	14.97	0.0001
(nonsurvivor vs survivor) 16	1	13.45	0.0002
(nonsurvivor vs survivor) 17	1	11.72	0.0006
(nonsurvivor vs survivor) 18	1	9.83	0.0017
(nonsurvivor vs survivor) 19	1	7.89	0.0050
(nonsurvivor vs survivor) 20	1	5.98	0.0144
(nonsurvivor vs survivor) 21	1	4.22	0.0399
(nonsurvivor vs survivor) 22	1	2.71	0.1000
Joint	5	56.45	0.0000

	Contrast	Delta-method Std. Err.	[95% Conf.	Interval]
death_30@_at				
(nonsurvivor vs survivor) 1	2.337763	1.271823	-.1549639	4.83049
(nonsurvivor vs survivor) 2	2.737729	1.214203	.3579347	5.117522
(nonsurvivor vs survivor) 3	3.098205	1.167916	.8091323	5.387277
(nonsurvivor vs survivor) 4	3.4188	1.132068	1.199986	5.637613
(nonsurvivor vs survivor) 5	3.699153	1.105514	1.532385	5.865921
(nonsurvivor vs survivor) 6	3.938936	1.086933	1.808587	6.069285
(nonsurvivor vs survivor) 7	4.137852	1.074934	2.03102	6.244684
(nonsurvivor vs survivor) 8	4.295637	1.068166	2.202071	6.389203
(nonsurvivor vs survivor) 9	4.412061	1.065404	2.323909	6.500214
(nonsurvivor vs survivor) 10	4.486927	1.065625	2.398341	6.575513
(nonsurvivor vs survivor) 11	4.520073	1.068056	2.426722	6.613424
(nonsurvivor vs survivor) 12	4.51137	1.072199	2.409899	6.612841
(nonsurvivor vs survivor) 13	4.460724	1.077846	2.348186	6.573263
(nonsurvivor vs survivor) 14	4.368077	1.085077	2.241365	6.494789
(nonsurvivor vs survivor) 15	4.233403	1.094256	2.088701	6.378104
(nonsurvivor vs survivor) 16	4.056712	1.106006	1.888981	6.224443
(nonsurvivor vs survivor) 17	3.83805	1.121187	1.640565	6.035535
(nonsurvivor vs survivor) 18	3.577494	1.140848	1.341473	5.813516
(nonsurvivor vs survivor) 19	3.275159	1.166173	.9895012	5.560817
(nonsurvivor vs survivor) 20	2.931191	1.198403	.5823646	5.280018
(nonsurvivor vs survivor) 21	2.545771	1.238755	.1178566	4.973686
(nonsurvivor vs survivor) 22	2.119113	1.28834	-.4059876	4.644214

```
285 . graph save "Graph" "/Users/Chiara/Documents/fileDO 15 Maggio/Marg_MCV.gph", replace
    (file /Users/Chiara/Documents/fileDO 15 Maggio/Marg_MCV.gph saved)

286 .
287 . *margins death_30, at(b_day_malattia=(0(1)30))  expression(exp(predict(xb)))
288 . *marginsplot, title("MCV kinetic day 0 to day 30 after onset") ytitle("Cell volume (fL") xlab(0(5)30) ylab(75(5)10
    > 0, angle(horizontal)) yline(80, lcolor(red))legend (off)
289 . *margins ar.death_30, at(b_day_malattia=(0(1)30))  expression(exp(predict(xb)))
290 .
291 . ***combine*****
292 . gr combine "/Users/Chiara/Documents/fileDO 15 Maggio/Marg_MCV.gph" "/Users/Chiara/Documents/fileDO 15 Maggio/BOX_M
    > CV.gph", ycomm   xsize(8) ysize(4)

293 . graph save "Graph" "/Users/Chiara/Documents/fileDO 15 Maggio/Graph_MCV.gph", replace
    (file /Users/Chiara/Documents/fileDO 15 Maggio/Graph_MCV.gph saved)

294 .
295 . ****************************************************************************
296 . *** analisi RDW-CV     ***************************************************
297 . ****************************************************************************
298 . **** scatter no model
299 . use "/Users/Chiara/Documents/fileDO 15 Maggio/fileDO\coorte_long_emocromo_anemia_1.dta", clear

300 . *scatter rdwcv b_day_malattia  if death_30==1, mcolor(red) legend(off) title("RDW-CV% variation over time") xtitle
    > ("day since onset") ytitle("RDW-CV (%)") xlab(0(5)30)|| scatter rdwcv b_day_malattia  if death_30==0, mcolor(green
    > ) legend(off) ms(oh)
301 . *keep if rdwcv!=.
302 .
303 . ********Graph box**************************************
304 . graph box rdwcv, over(death_30, label(nolabel)) over(b_day_malattia) asyvars box(1, fcolor(navy))  nooutsides ytit
    > le(Cells volume (fl)) yline(14, lcolor(red)) legend(off)

305 . graph save "Graph" "/Users/Chiara/Documents/fileDO 15 Maggio/BOX_RDW.gph", replace
    (file /Users/Chiara/Documents/fileDO 15 Maggio/BOX_RDW.gph saved)

306 .
307 . **** modelling
308 . use "/Users/Chiara/Documents/fileDO 15 Maggio/fileDO\coorte_long_emocromo_anemia_1.dta", clear

309 . mixed rdwcv_ln c.b_day_malattia##i.death_30 || progr:b_day_malattia, cov(unstr)

    Performing EM optimization:

    Performing gradient-based optimization:

    Iteration 0:   log likelihood =  3194.0524
    Iteration 1:   log likelihood =  3194.0531
    Iteration 2:   log likelihood =  3194.0531

    Computing standard errors:

    Mixed-effects ML regression                    Number of obs     =      1,805
    Group variable: progr                          Number of groups  =        379

                                                   Obs per group:
                                                                 min =          1
                                                                 avg =        4.8
                                                                 max =         22

                                                   Wald chi2(3)      =      83.89
    Log likelihood =  3194.0531                    Prob > chi2       =     0.0000
```

rdwcv_ln	Coef.	Std. Err.	z	P>|z|	[95% Conf.	Interval]

```
          b_day_malattia |   .0006332   .0003203     1.98   0.048     5.45e-06     .001261
                         |
                death_30 |
             nonsurvivor |    .067003    .016913     3.96   0.000     .0338541    .1001518
                         |
death_30#c.b_day_malattia |
             nonsurvivor |   .0053289   .0009825     5.42   0.000     .0034032    .0072546
                         |
                   _cons |    2.56952   .0058591   438.55   0.000     2.558037    2.581004
------------------------------------------------------------------------------------------

------------------------------------------------------------------------------
  Random-effects Parameters  |   Estimate   Std. Err.     [95% Conf. Interval]
-----------------------------+------------------------------------------------
progr: Unstructured          |
         var(b_day_malattia) |   .0000165   1.99e-06       .000013    .0000209
                  var(_cons) |   .0089686   .0007812       .007561    .0106382
   cov(b_day_malattia,_cons) |  -.0000786     .00003     -.0001375   -.0000198
-----------------------------+------------------------------------------------
               var(Residual) |   .0006158    .000025      .0005686    .0006668
------------------------------------------------------------------------------
LR test vs. linear model: chi2(3) = 3387.52               Prob > chi2 = 0.0000

Note: LR test is conservative and provided only for reference.

310 . est store linear

311 . mixed rdwcv_ln  c.b_day_malattia##c.b_day_malattia##i.death_30 i.p_age2 i.p_cardio i.p_obeso i.p_rene || progr:b_d
    > ay_malattia, cov(unstr)

    Performing EM optimization:

    Performing gradient-based optimization:

    Iteration 0:   log likelihood =  3237.8802
    Iteration 1:   log likelihood =  3237.8807
    Iteration 2:   log likelihood =  3237.8807

    Computing standard errors:

    Mixed-effects ML regression                     Number of obs     =      1,805
    Group variable: progr                           Number of groups  =        379

                                                    Obs per group:
                                                                  min =          1
                                                                  avg =        4.8
                                                                  max =         22

                                                    Wald chi2(9)      =     186.47
    Log likelihood =  3237.8807                     Prob > chi2       =     0.0000

    ------------------------------------------------------------------------------------------------
                                   rdwcv_ln |      Coef.   Std. Err.      z    P>|z|     [95% Conf. Interval]
    ----------------------------------------+-------------------------------------------------------
                             b_day_malattia |  -.0006197   .0008029    -0.77   0.440    -.0021933     .000954
                                            |
            c.b_day_malattia#c.b_day_malattia |   .0000504   .0000299     1.69   0.092    -8.22e-06     .000109
                                            |
                                   death_30 |
                                nonsurvivor |   .0548367   .0188874     2.90   0.004      .017818    .0918554
                                            |
                  death_30#c.b_day_malattia |
                                nonsurvivor |  -.0017401   .0020595    -0.84   0.398    -.0057767    .0022964
                                            |
    death_30#c.b_day_malattia#c.b_day_malattia |
                                nonsurvivor |   .0003325   .0000822     4.05   0.000     .0001714    .0004935
                                            |
                                   1.p_age2 |   .0355318   .0099427     3.57   0.000     .0160444    .0550192
                                 1.p_cardio |    .039348   .0106709     3.69   0.000     .0184334    .0602626
                                  1.p_obeso |  -.0000827   .0189147    -0.00   0.997    -.0371548    .0369894
                                   1.p_rene |   .0855343   .0219689     3.89   0.000     .0424761    .1285925
                                      _cons |   2.542967   .0086469   294.09   0.000     2.526019    2.559915
    ------------------------------------------------------------------------------------------------

    ------------------------------------------------------------------------------
      Random-effects Parameters  |   Estimate   Std. Err.     [95% Conf. Interval]
    -----------------------------+------------------------------------------------
    progr: Unstructured          |
             var(b_day_malattia) |   .0000167   2.00e-06      .0000132    .0000212
                      var(_cons) |   .0079089    .000708      .0066361    .0094259
       cov(b_day_malattia,_cons) |  -.0001011   .0000294     -.0001588   -.0000434
    -----------------------------+------------------------------------------------
                   var(Residual) |   .0006023   .0000245      .0005561    .0006523
    ------------------------------------------------------------------------------
    LR test vs. linear model: chi2(3) = 3166.07               Prob > chi2 = 0.0000

    Note: LR test is conservative and provided only for reference.

312 . est store quadratic

313 . lrtest linear quadratic

    Likelihood-ratio test                                 LR chi2(6)  =     87.66
    (Assumption: linear nested in quadratic)              Prob > chi2 =    0.0000

314 . *****margins*******
315 . quiet:mixed rdwcv_ln  c.b_day_malattia##c.b_day_malattia##i.death_30 i.p_age2 i.p_cardio i.p_obeso i.p_rene || pro
    > gr:b_day_malattia, cov(unstr)

316 . margins death_30, at(b_day_malattia=(0(1)21))  expression(exp(predict(xb))) asbal

    Adjusted predictions                            Number of obs     =      1,805

    Expression   : exp(predict(xb))

    1._at        : b_day_mala~a    =           0
                   death_30                     (asbalanced)
                   p_age2                       (asbalanced)
```

```
                 p_cardio                      (asbalanced)
                 p_obeso                       (asbalanced)
                 p_rene                        (asbalanced)

2._at          : b_day_mala~a    =           1
                 death_30                      (asbalanced)
                 p_age2                        (asbalanced)
                 p_cardio                      (asbalanced)
                 p_obeso                       (asbalanced)
                 p_rene                        (asbalanced)

3._at          : b_day_mala~a    =           2
                 death_30                      (asbalanced)
                 p_age2                        (asbalanced)
                 p_cardio                      (asbalanced)
                 p_obeso                       (asbalanced)
                 p_rene                        (asbalanced)

4._at          : b_day_mala~a    =           3
                 death_30                      (asbalanced)
                 p_age2                        (asbalanced)
                 p_cardio                      (asbalanced)
                 p_obeso                       (asbalanced)
                 p_rene                        (asbalanced)

5._at          : b_day_mala~a    =           4
                 death_30                      (asbalanced)
                 p_age2                        (asbalanced)
                 p_cardio                      (asbalanced)
                 p_obeso                       (asbalanced)
                 p_rene                        (asbalanced)

6._at          : b_day_mala~a    =           5
                 death_30                      (asbalanced)
                 p_age2                        (asbalanced)
                 p_cardio                      (asbalanced)
                 p_obeso                       (asbalanced)
                 p_rene                        (asbalanced)

7._at          : b_day_mala~a    =           6
                 death_30                      (asbalanced)
                 p_age2                        (asbalanced)
                 p_cardio                      (asbalanced)
                 p_obeso                       (asbalanced)
                 p_rene                        (asbalanced)

8._at          : b_day_mala~a    =           7
                 death_30                      (asbalanced)
                 p_age2                        (asbalanced)
                 p_cardio                      (asbalanced)
                 p_obeso                       (asbalanced)
                 p_rene                        (asbalanced)

9._at          : b_day_mala~a    =           8
                 death_30                      (asbalanced)
                 p_age2                        (asbalanced)
                 p_cardio                      (asbalanced)
                 p_obeso                       (asbalanced)
                 p_rene                        (asbalanced)

10._at         : b_day_mala~a    =           9
                 death_30                      (asbalanced)
                 p_age2                        (asbalanced)
                 p_cardio                      (asbalanced)
                 p_obeso                       (asbalanced)
                 p_rene                        (asbalanced)

11._at         : b_day_mala~a    =          10
                 death_30                      (asbalanced)
                 p_age2                        (asbalanced)
                 p_cardio                      (asbalanced)
                 p_obeso                       (asbalanced)
                 p_rene                        (asbalanced)

12._at         : b_day_mala~a    =          11
                 death_30                      (asbalanced)
                 p_age2                        (asbalanced)
                 p_cardio                      (asbalanced)
                 p_obeso                       (asbalanced)
                 p_rene                        (asbalanced)

13._at         : b_day_mala~a    =          12
                 death_30                      (asbalanced)
                 p_age2                        (asbalanced)
                 p_cardio                      (asbalanced)
                 p_obeso                       (asbalanced)
                 p_rene                        (asbalanced)

14._at         : b_day_mala~a    =          13
                 death_30                      (asbalanced)
                 p_age2                        (asbalanced)
                 p_cardio                      (asbalanced)
                 p_obeso                       (asbalanced)
                 p_rene                        (asbalanced)

15._at         : b_day_mala~a    =          14
                 death_30                      (asbalanced)
                 p_age2                        (asbalanced)
                 p_cardio                      (asbalanced)
                 p_obeso                       (asbalanced)
                 p_rene                        (asbalanced)

16._at         : b_day_mala~a    =          15
                 death_30                      (asbalanced)
                 p_age2                        (asbalanced)
                 p_cardio                      (asbalanced)
                 p_obeso                       (asbalanced)
                 p_rene                        (asbalanced)
```

```
17._at       : b_day_mala~a    =          16
               death_30                      (asbalanced)
               p_age2                        (asbalanced)
               p_cardio                      (asbalanced)
               p_obeso                       (asbalanced)
               p_rene                        (asbalanced)

18._at       : b_day_mala~a    =          17
               death_30                      (asbalanced)
               p_age2                        (asbalanced)
               p_cardio                      (asbalanced)
               p_obeso                       (asbalanced)
               p_rene                        (asbalanced)

19._at       : b_day_mala~a    =          18
               death_30                      (asbalanced)
               p_age2                        (asbalanced)
               p_cardio                      (asbalanced)
               p_obeso                       (asbalanced)
               p_rene                        (asbalanced)

20._at       : b_day_mala~a    =          19
               death_30                      (asbalanced)
               p_age2                        (asbalanced)
               p_cardio                      (asbalanced)
               p_obeso                       (asbalanced)
               p_rene                        (asbalanced)

21._at       : b_day_mala~a    =          20
               death_30                      (asbalanced)
               p_age2                        (asbalanced)
               p_cardio                      (asbalanced)
               p_obeso                       (asbalanced)
               p_rene                        (asbalanced)

22._at       : b_day_mala~a    =          21
               death_30                      (asbalanced)
               p_age2                        (asbalanced)
               p_cardio                      (asbalanced)
               p_obeso                       (asbalanced)
               p_rene                        (asbalanced)
```

		Delta-method				
	Margin	Std. Err.	z	P>|z|	[95% Conf.	Interval]
_at#death_30						
1#survivor	13.77882	.2107385	65.38	0.000	13.36578	14.19186
1#nonsurvivor	14.55551	.280021	51.98	0.000	14.00668	15.10434
2#survivor	13.77098	.2074847	66.37	0.000	13.36432	14.17764
2#nonsurvivor	14.52676	.2676984	54.27	0.000	14.00208	15.05144
3#survivor	13.76453	.2049122	67.17	0.000	13.36291	14.16615
3#nonsurvivor	14.50917	.2583346	56.16	0.000	14.00285	15.0155
4#survivor	13.75947	.2029351	67.80	0.000	13.36172	14.15721
4#nonsurvivor	14.50271	.251644	57.63	0.000	14.0095	14.99592
5#survivor	13.7558	.2014696	68.28	0.000	13.36092	14.15067
5#nonsurvivor	14.50735	.2473073	58.66	0.000	14.02264	14.99207
6#survivor	13.75351	.2004359	68.62	0.000	13.36066	14.14636
6#nonsurvivor	14.52312	.2449919	59.28	0.000	14.04294	15.00329
7#survivor	13.75261	.1997604	68.85	0.000	13.36109	14.14414
7#nonsurvivor	14.55003	.2443731	59.54	0.000	14.07107	15.02899
8#survivor	13.7531	.1993769	68.98	0.000	13.36233	14.14387
8#nonsurvivor	14.58816	.2451543	59.51	0.000	14.10767	15.06866
9#survivor	13.75497	.1992277	69.04	0.000	13.36449	14.14545
9#nonsurvivor	14.6376	.2470825	59.24	0.000	14.15332	15.12187
10#survivor	13.75823	.1992644	69.05	0.000	13.36768	14.14878
10#nonsurvivor	14.69845	.2499585	58.80	0.000	14.20854	15.18836
11#survivor	13.76288	.1994482	69.00	0.000	13.37197	14.15379
11#nonsurvivor	14.77086	.2536426	58.23	0.000	14.27373	15.26799
12#survivor	13.76892	.1997507	68.93	0.000	13.37741	14.16042
12#nonsurvivor	14.855	.2580565	57.56	0.000	14.34922	15.36078
13#survivor	13.77634	.2001531	68.83	0.000	13.38405	14.16864
13#nonsurvivor	14.95106	.2631841	56.81	0.000	14.43523	15.46689
14#survivor	13.78516	.2006473	68.70	0.000	13.3919	14.17843
14#nonsurvivor	15.05927	.2690695	55.97	0.000	14.5319	15.58663
15#survivor	13.79538	.201235	68.55	0.000	13.40097	14.18979
15#nonsurvivor	15.17988	.2758156	55.04	0.000	14.63929	15.72046
16#survivor	13.807	.2019282	68.38	0.000	13.41123	14.20277
16#nonsurvivor	15.31317	.283581	54.00	0.000	14.75736	15.86898
17#survivor	13.82002	.2027487	68.16	0.000	13.42264	14.2174
17#nonsurvivor	15.45947	.2925762	52.84	0.000	14.88603	16.03291
18#survivor	13.83444	.2037284	67.91	0.000	13.43514	14.23374
18#nonsurvivor	15.61913	.3030585	51.54	0.000	15.02514	16.21311
19#survivor	13.85028	.2049083	67.59	0.000	13.44866	14.25189
19#nonsurvivor	15.79251	.3153252	50.08	0.000	15.17449	16.41054
20#survivor	13.86753	.2063391	67.21	0.000	13.46311	14.27194
20#nonsurvivor	15.98006	.3297051	48.47	0.000	15.33385	16.62627
21#survivor	13.8862	.2080796	66.74	0.000	13.47837	14.29403
21#nonsurvivor	16.18222	.3465489	46.70	0.000	15.503	16.86144
22#survivor	13.9063	.2101966	66.16	0.000	13.49432	14.31827
22#nonsurvivor	16.39949	.3662199	44.78	0.000	15.68171	17.11726

```
317 . marginsplot, ytitle("RDW %") xlab(0(1)21) ylab(10(2)18) yline(14, lcolor(red)) legend(off)

      Variables that uniquely identify margins: b_day_malattia death_30

318 . margins ar.death_30, at(b_day_malattia=(0(1)21))  expression(exp(predict(xb))) asbal

    Contrasts of adjusted predictions               Number of obs     =      1,805

    Expression   : exp(predict(xb))

    1._at        : b_day_mala~a    =           0
                   death_30                      (asbalanced)
                   p_age2                        (asbalanced)
                   p_cardio                      (asbalanced)
```

```
                p_obeso                        (asbalanced)
                p_rene                         (asbalanced)

2._at         : b_day_mala~a    =            1
                death_30                       (asbalanced)
                p_age2                         (asbalanced)
                p_cardio                       (asbalanced)
                p_obeso                        (asbalanced)
                p_rene                         (asbalanced)

3._at         : b_day_mala~a    =            2
                death_30                       (asbalanced)
                p_age2                         (asbalanced)
                p_cardio                       (asbalanced)
                p_obeso                        (asbalanced)
                p_rene                         (asbalanced)

4._at         : b_day_mala~a    =            3
                death_30                       (asbalanced)
                p_age2                         (asbalanced)
                p_cardio                       (asbalanced)
                p_obeso                        (asbalanced)
                p_rene                         (asbalanced)

5._at         : b_day_mala~a    =            4
                death_30                       (asbalanced)
                p_age2                         (asbalanced)
                p_cardio                       (asbalanced)
                p_obeso                        (asbalanced)
                p_rene                         (asbalanced)

6._at         : b_day_mala~a    =            5
                death_30                       (asbalanced)
                p_age2                         (asbalanced)
                p_cardio                       (asbalanced)
                p_obeso                        (asbalanced)
                p_rene                         (asbalanced)

7._at         : b_day_mala~a    =            6
                death_30                       (asbalanced)
                p_age2                         (asbalanced)
                p_cardio                       (asbalanced)
                p_obeso                        (asbalanced)
                p_rene                         (asbalanced)

8._at         : b_day_mala~a    =            7
                death_30                       (asbalanced)
                p_age2                         (asbalanced)
                p_cardio                       (asbalanced)
                p_obeso                        (asbalanced)
                p_rene                         (asbalanced)

9._at         : b_day_mala~a    =            8
                death_30                       (asbalanced)
                p_age2                         (asbalanced)
                p_cardio                       (asbalanced)
                p_obeso                        (asbalanced)
                p_rene                         (asbalanced)

10._at        : b_day_mala~a    =            9
                death_30                       (asbalanced)
                p_age2                         (asbalanced)
                p_cardio                       (asbalanced)
                p_obeso                        (asbalanced)
                p_rene                         (asbalanced)

11._at        : b_day_mala~a    =           10
                death_30                       (asbalanced)
                p_age2                         (asbalanced)
                p_cardio                       (asbalanced)
                p_obeso                        (asbalanced)
                p_rene                         (asbalanced)

12._at        : b_day_mala~a    =           11
                death_30                       (asbalanced)
                p_age2                         (asbalanced)
                p_cardio                       (asbalanced)
                p_obeso                        (asbalanced)
                p_rene                         (asbalanced)

13._at        : b_day_mala~a    =           12
                death_30                       (asbalanced)
                p_age2                         (asbalanced)
                p_cardio                       (asbalanced)
                p_obeso                        (asbalanced)
                p_rene                         (asbalanced)

14._at        : b_day_mala~a    =           13
                death_30                       (asbalanced)
                p_age2                         (asbalanced)
                p_cardio                       (asbalanced)
                p_obeso                        (asbalanced)
                p_rene                         (asbalanced)

15._at        : b_day_mala~a    =           14
                death_30                       (asbalanced)
                p_age2                         (asbalanced)
                p_cardio                       (asbalanced)
                p_obeso                        (asbalanced)
                p_rene                         (asbalanced)

16._at        : b_day_mala~a    =           15
                death_30                       (asbalanced)
                p_age2                         (asbalanced)
                p_cardio                       (asbalanced)
                p_obeso                        (asbalanced)
                p_rene                         (asbalanced)
```

```
17._at       : b_day_mala~a    =          16
               death_30                      (asbalanced)
               p_age2                        (asbalanced)
               p_cardio                      (asbalanced)
               p_obeso                       (asbalanced)
               p_rene                        (asbalanced)

18._at       : b_day_mala~a    =          17
               death_30                      (asbalanced)
               p_age2                        (asbalanced)
               p_cardio                      (asbalanced)
               p_obeso                       (asbalanced)
               p_rene                        (asbalanced)

19._at       : b_day_mala~a    =          18
               death_30                      (asbalanced)
               p_age2                        (asbalanced)
               p_cardio                      (asbalanced)
               p_obeso                       (asbalanced)
               p_rene                        (asbalanced)

20._at       : b_day_mala~a    =          19
               death_30                      (asbalanced)
               p_age2                        (asbalanced)
               p_cardio                      (asbalanced)
               p_obeso                       (asbalanced)
               p_rene                        (asbalanced)

21._at       : b_day_mala~a    =          20
               death_30                      (asbalanced)
               p_age2                        (asbalanced)
               p_cardio                      (asbalanced)
               p_obeso                       (asbalanced)
               p_rene                        (asbalanced)

22._at       : b_day_mala~a    =          21
               death_30                      (asbalanced)
               p_age2                        (asbalanced)
               p_cardio                      (asbalanced)
               p_obeso                       (asbalanced)
               p_rene                        (asbalanced)
```

	df	chi2	P>chi2
death_30@_at			
(nonsurvivor vs survivor) 1	1	8.24	0.0041
(nonsurvivor vs survivor) 2	1	8.75	0.0031
(nonsurvivor vs survivor) 3	1	9.33	0.0022
(nonsurvivor vs survivor) 4	1	10.00	0.0016
(nonsurvivor vs survivor) 5	1	10.76	0.0010
(nonsurvivor vs survivor) 6	1	11.64	0.0006
(nonsurvivor vs survivor) 7	1	12.66	0.0004
(nonsurvivor vs survivor) 8	1	13.85	0.0002
(nonsurvivor vs survivor) 9	1	15.26	0.0001
(nonsurvivor vs survivor) 10	1	16.92	0.0000
(nonsurvivor vs survivor) 11	1	18.85	0.0000
(nonsurvivor vs survivor) 12	1	21.09	0.0000
(nonsurvivor vs survivor) 13	1	23.64	0.0000
(nonsurvivor vs survivor) 14	1	26.50	0.0000
(nonsurvivor vs survivor) 15	1	29.64	0.0000
(nonsurvivor vs survivor) 16	1	33.01	0.0000
(nonsurvivor vs survivor) 17	1	36.51	0.0000
(nonsurvivor vs survivor) 18	1	40.02	0.0000
(nonsurvivor vs survivor) 19	1	43.40	0.0000
(nonsurvivor vs survivor) 20	1	46.50	0.0000
(nonsurvivor vs survivor) 21	1	49.17	0.0000
(nonsurvivor vs survivor) 22	1	51.30	0.0000
Joint	5	165.28	0.0000

	Contrast	Delta-method Std. Err.	[95% Conf.	Interval]
death_30@_at				
(nonsurvivor vs survivor) 1	.7766853	.2706044	.2463104	1.30706
(nonsurvivor vs survivor) 2	.7557797	.2554936	.2550216	1.256538
(nonsurvivor vs survivor) 3	.7446448	.2437229	.2669567	1.222333
(nonsurvivor vs survivor) 4	.7432419	.2350389	.2825741	1.20391
(nonsurvivor vs survivor) 5	.7515579	.2291253	.3024805	1.200635
(nonsurvivor vs survivor) 6	.7696045	.2256223	.3273928	1.211816
(nonsurvivor vs survivor) 7	.7974185	.2241547	.3580834	1.236754
(nonsurvivor vs survivor) 8	.8350623	.2243618	.3953213	1.274803
(nonsurvivor vs survivor) 9	.8826236	.2259236	.4398215	1.325426
(nonsurvivor vs survivor) 10	.9402159	.2285798	.4922077	1.388224
(nonsurvivor vs survivor) 11	1.007979	.2321408	.5529915	1.462967
(nonsurvivor vs survivor) 12	1.08608	.2364927	.6225631	1.549597
(nonsurvivor vs survivor) 13	1.174713	.2415978	.70119	1.648236
(nonsurvivor vs survivor) 14	1.274101	.2474936	.789022	1.759179
(nonsurvivor vs survivor) 15	1.384494	.2542883	.8860979	1.88289
(nonsurvivor vs survivor) 16	1.506175	.2621571	.9923567	2.019994
(nonsurvivor vs survivor) 17	1.639457	.271335	1.10765	2.171264
(nonsurvivor vs survivor) 18	1.784685	.2821089	1.231762	2.337609
(nonsurvivor vs survivor) 19	1.942239	.2948071	1.364428	2.520051
(nonsurvivor vs survivor) 20	2.112534	.309787	1.505362	2.719705
(nonsurvivor vs survivor) 21	2.29602	.327422	1.654285	2.937756
(nonsurvivor vs survivor) 22	2.49319	.3480892	1.810948	3.175433

```
319 . graph save "Graph" "/Users/Chiara/Documents/fileDO 15 Maggio/Marg_RDW.gph", replace
    (file /Users/Chiara/Documents/fileDO 15 Maggio/Marg_RDW.gph saved)

320 .
321 . *margins death_30, at(b_day_malattia=(0(1)30))  expression(exp(predict(xb)))
322 . *marginsplot, title("RDW-CV% kinetic day 0 to day 30 after onset") ytitle("RDW (%)") xlab(0(5)30) ylab(10(2)20, an
    > gle(horizontal)) yline(14, lcolor(red)) legend(off)
323 . *margins ar.death_30, at(b_day_malattia=(0(1)30))  expression(exp(predict(xb)))
```

```
324 .
325 . ***combine*****
326 . gr combine "/Users/Chiara/Documents/fileDO 15 Maggio/Marg_RDW.gph" "/Users/Chiara/Documents/fileDO 15 Maggio/BOX_R
    > DW.gph", ycomm   xsize(8) ysize(4)

327 . graph save "Graph" "/Users/Chiara/Documents/fileDO 15 Maggio/Graph_RDW.gph", replace
    (file /Users/Chiara/Documents/fileDO 15 Maggio/Graph_RDW.gph saved)

328 .
329 . ***************************************************************************
330 . *** analisi RBC***************************************************
331 . ***************************************************************************
332 . **** scatter no model
333 . use "/Users/Chiara/Documents/fileDO 15 Maggio/fileDO\coorte_long_emocromo_anemia_1.dta", clear

334 . *scatter rbc b_day_malattia  if death_30==1, mcolor(red) legend(off) title("RBC variation over time") xtitle("day
    > since onset") ytitle("RBC per mmc X 1000000") xlab(0(5)30)|| scatter rbc b_day_malattia  if death_30==0, mcolor(gr
    > een) legend(off) ms(oh)
335 . *keep if rdwcv!=.
336 . *********Graph box*****************************************
337 . replace rbc=rbc*1000000
    (1,805 real changes made)

338 . graph box rbc, over(death_30, label(nolabel)) over(b_day_malattia) asyvars box(1, fcolor(navy))  nooutsides ytitle
    > (Cells per mmc x 1000000) yline(3000000, lcolor(red)) legend(off)

339 . graph save "Graph" "/Users/Chiara/Documents/fileDO 15 Maggio/BOX_RBC.gph", replace
    (file /Users/Chiara/Documents/fileDO 15 Maggio/BOX_RBC.gph saved)

340 . **** modelling
341 . use "/Users/Chiara/Documents/fileDO 15 Maggio/fileDO\coorte_long_emocromo_anemia_1.dta", clear

342 . mixed rbc_ln c.b_day_malattia##i.death_30 || progr:b_day_malattia, cov(unstr)

    Performing EM optimization:

    Performing gradient-based optimization:

    Iteration 0:   log likelihood =  1661.5037
    Iteration 1:   log likelihood =  1661.5103
    Iteration 2:   log likelihood =  1661.5103

    Computing standard errors:

    Mixed-effects ML regression                     Number of obs     =      1,805
    Group variable: progr                           Number of groups  =        379

                                                    Obs per group:
                                                                  min =          1
                                                                  avg =        4.8
                                                                  max =         22

                                                    Wald chi2(3)      =     129.70
    Log likelihood =  1661.5103                     Prob > chi2       =     0.0000
```

rbc_ln	Coef.	Std. Err.	z	P>|z|	[95% Conf.	Interval]
b_day_malattia	-.0059862	.0007063	-8.48	0.000	-.0073706	-.0046018
death_30						
nonsurvivor	-.1346529	.0307841	-4.37	0.000	-.1949886	-.0743173
death_30#c.b_day_malattia						
nonsurvivor	-.0034302	.0021124	-1.62	0.104	-.0075704	.0007099
_cons	15.38927	.0110902	1387.65	0.000	15.36753	15.411

Random-effects Parameters	Estimate	Std. Err.	[95% Conf.	Interval]
**progr**: Unstructured				
var(b_day_malattia)	.0000655	9.52e-06	.0000493	.0000871
var(_cons)	.0259037	.0026059	.0212682	.0315496
cov(b_day_malattia,_cons)	-.0005826	.0001341	-.0008455	-.0003197
var(Residual)	.0044198	.0001783	.0040837	.0047836

```
    LR test vs. linear model: chi2(3) = 2292.52              Prob > chi2 = 0.0000

    Note: LR test is conservative and provided only for reference.

343 . est store linear

344 . mixed rbc_ln  c.b_day_malattia##c.b_day_malattia##i.death_30 i.p_age2 i.p_cardio i.p_obeso i.p_rene || progr:b_day
    > _malattia, cov(unstr)

    Performing EM optimization:

    Performing gradient-based optimization:

    Iteration 0:   log likelihood =  1694.3095
    Iteration 1:   log likelihood =  1694.3168
    Iteration 2:   log likelihood =  1694.3168

    Computing standard errors:

    Mixed-effects ML regression                     Number of obs     =      1,805
    Group variable: progr                           Number of groups  =        379

                                                    Obs per group:
                                                                  min =          1
                                                                  avg =        4.8
                                                                  max =         22

                                                    Wald chi2(9)      =     207.08
    Log likelihood =  1694.3168                     Prob > chi2       =     0.0000
```

```
------------------------------------------------------------------------------------------------------------
                                     rbc_ln |      Coef.   Std. Err.      z    P>|z|     [95% Conf. Interval]
--------------------------------------------+---------------------------------------------------------------
                             b_day_malattia |  -.0073368    .002025    -3.62   0.000    -.0113056   -.0033679
                                            |
        c.b_day_malattia#c.b_day_malattia |   .0000585   .0000771     0.76   0.448    -.0000926    .0002096
                                            |
                                   death_30 |
                                nonsurvivor |  -.0426858   .0380358    -1.12   0.262    -.1172345    .0318629
                                            |
                    death_30#c.b_day_malattia |
                                nonsurvivor |  -.0130316   .0051749    -2.52   0.012    -.0231742    -.002889
                                            |
death_30#c.b_day_malattia#c.b_day_malattia |
                                nonsurvivor |   .0004259   .0002116     2.01   0.044     .0000111    .0008407
                                            |
                                  1.p_age2 |  -.0644802   .0157717    -4.09   0.000     -.095392   -.0335683
                                1.p_cardio |   .0093844   .0169037     0.56   0.579    -.0237463    .0425151
                                 1.p_obeso |   .0368329   .0299484     1.23   0.219    -.0218649    .0955307
                                  1.p_rene |  -.2186919   .0348034    -6.28   0.000    -.2869053   -.1504786
                                     _cons |   15.43243   .0170308   906.15   0.000     15.39905    15.46581
------------------------------------------------------------------------------------------------------------

------------------------------------------------------------------------------
  Random-effects Parameters  |   Estimate   Std. Err.     [95% Conf. Interval]
-----------------------------+------------------------------------------------
progr: Unstructured          |
         var(b_day_malattia) |   .0000694     .00001      .0000523     .000092
                   var(_cons) |   .0245928   .0025468      .0200752     .030127
   cov(b_day_malattia,_cons) |  -.0006992   .0001403     -.0009741   -.0004242
-----------------------------+------------------------------------------------
               var(Residual) |   .0043675   .0001767      .0040346     .004728
------------------------------------------------------------------------------
LR test vs. linear model: chi2(3) = 2164.87               Prob > chi2 = 0.0000

Note: LR test is conservative and provided only for reference.

345 . est store quadratic

346 . lrtest linear quadratic

    Likelihood-ratio test                                 LR chi2(6)  =     65.61
    (Assumption: linear nested in quadratic)              Prob > chi2 =    0.0000

347 . ***margins*******
348 . quiet: mixed rbc_ln  c.b_day_malattia##c.b_day_malattia##i.death_30 i.p_age2 i.p_cardio i.p_obeso i.p_rene || prog
    > r:b_day_malattia, cov(unstr)

349 . margins death_30, at(b_day_malattia=(0(1)21))  expression(exp(predict(xb))) asbal

    Adjusted predictions                           Number of obs     =      1,805

    Expression   : exp(predict(xb))

    1._at        : b_day_mala~a    =           0
                   death_30                      (asbalanced)
                   p_age2                        (asbalanced)
                   p_cardio                      (asbalanced)
                   p_obeso                       (asbalanced)
                   p_rene                        (asbalanced)

    2._at        : b_day_mala~a    =           1
                   death_30                      (asbalanced)
                   p_age2                        (asbalanced)
                   p_cardio                      (asbalanced)
                   p_obeso                       (asbalanced)
                   p_rene                        (asbalanced)

    3._at        : b_day_mala~a    =           2
                   death_30                      (asbalanced)
                   p_age2                        (asbalanced)
                   p_cardio                      (asbalanced)
                   p_obeso                       (asbalanced)
                   p_rene                        (asbalanced)

    4._at        : b_day_mala~a    =           3
                   death_30                      (asbalanced)
                   p_age2                        (asbalanced)
                   p_cardio                      (asbalanced)
                   p_obeso                       (asbalanced)
                   p_rene                        (asbalanced)

    5._at        : b_day_mala~a    =           4
                   death_30                      (asbalanced)
                   p_age2                        (asbalanced)
                   p_cardio                      (asbalanced)
                   p_obeso                       (asbalanced)
                   p_rene                        (asbalanced)

    6._at        : b_day_mala~a    =           5
                   death_30                      (asbalanced)
                   p_age2                        (asbalanced)
                   p_cardio                      (asbalanced)
                   p_obeso                       (asbalanced)
                   p_rene                        (asbalanced)

    7._at        : b_day_mala~a    =           6
                   death_30                      (asbalanced)
                   p_age2                        (asbalanced)
                   p_cardio                      (asbalanced)
                   p_obeso                       (asbalanced)
                   p_rene                        (asbalanced)

    8._at        : b_day_mala~a    =           7
                   death_30                      (asbalanced)
                   p_age2                        (asbalanced)
                   p_cardio                      (asbalanced)
```

```
                p_obeso                      (asbalanced)
                p_rene                       (asbalanced)

9._at        : b_day_mala~a    =           8
                death_30                     (asbalanced)
                p_age2                       (asbalanced)
                p_cardio                     (asbalanced)
                p_obeso                      (asbalanced)
                p_rene                       (asbalanced)

10._at       : b_day_mala~a    =           9
                death_30                     (asbalanced)
                p_age2                       (asbalanced)
                p_cardio                     (asbalanced)
                p_obeso                      (asbalanced)
                p_rene                       (asbalanced)

11._at       : b_day_mala~a    =          10
                death_30                     (asbalanced)
                p_age2                       (asbalanced)
                p_cardio                     (asbalanced)
                p_obeso                      (asbalanced)
                p_rene                       (asbalanced)

12._at       : b_day_mala~a    =          11
                death_30                     (asbalanced)
                p_age2                       (asbalanced)
                p_cardio                     (asbalanced)
                p_obeso                      (asbalanced)
                p_rene                       (asbalanced)

13._at       : b_day_mala~a    =          12
                death_30                     (asbalanced)
                p_age2                       (asbalanced)
                p_cardio                     (asbalanced)
                p_obeso                      (asbalanced)
                p_rene                       (asbalanced)

14._at       : b_day_mala~a    =          13
                death_30                     (asbalanced)
                p_age2                       (asbalanced)
                p_cardio                     (asbalanced)
                p_obeso                      (asbalanced)
                p_rene                       (asbalanced)

15._at       : b_day_mala~a    =          14
                death_30                     (asbalanced)
                p_age2                       (asbalanced)
                p_cardio                     (asbalanced)
                p_obeso                      (asbalanced)
                p_rene                       (asbalanced)

16._at       : b_day_mala~a    =          15
                death_30                     (asbalanced)
                p_age2                       (asbalanced)
                p_cardio                     (asbalanced)
                p_obeso                      (asbalanced)
                p_rene                       (asbalanced)

17._at       : b_day_mala~a    =          16
                death_30                     (asbalanced)
                p_age2                       (asbalanced)
                p_cardio                     (asbalanced)
                p_obeso                      (asbalanced)
                p_rene                       (asbalanced)

18._at       : b_day_mala~a    =          17
                death_30                     (asbalanced)
                p_age2                       (asbalanced)
                p_cardio                     (asbalanced)
                p_obeso                      (asbalanced)
                p_rene                       (asbalanced)

19._at       : b_day_mala~a    =          18
                death_30                     (asbalanced)
                p_age2                       (asbalanced)
                p_cardio                     (asbalanced)
                p_obeso                      (asbalanced)
                p_rene                       (asbalanced)

20._at       : b_day_mala~a    =          19
                death_30                     (asbalanced)
                p_age2                       (asbalanced)
                p_cardio                     (asbalanced)
                p_obeso                      (asbalanced)
                p_rene                       (asbalanced)

21._at       : b_day_mala~a    =          20
                death_30                     (asbalanced)
                p_age2                       (asbalanced)
                p_cardio                     (asbalanced)
                p_obeso                      (asbalanced)
                p_rene                       (asbalanced)

22._at       : b_day_mala~a    =          21
                death_30                     (asbalanced)
                p_age2                       (asbalanced)
                p_cardio                     (asbalanced)
                p_obeso                      (asbalanced)
                p_rene                       (asbalanced)
```

	Margin	Delta-method Std. Err.	z	P>|z|	[95% Conf.	Interval]
_at#death_30						
1#survivor	4474709	117652.2	38.03	0.000	4244115	4705303
1#nonsurvivor	4287722	158954.8	26.97	0.000	3976176	4599267

```
        2#survivor  |   4442259   112971.6    39.32   0.000      4220839     4663679
     2#nonsurvivor  |   4203306   143880.2    29.21   0.000      3921306     4485307
        3#survivor  |   4410560   109097.2    40.43   0.000      4196733     4624386
     3#nonsurvivor  |   4124547   131690.1    31.32   0.000      3866439     4382655
        4#survivor  |   4379599   105934.8    41.34   0.000      4171970     4587227
     4#nonsurvivor  |   4051186   122118.6    33.17   0.000      3811838     4290534
        5#survivor  |   4349364   103385.9    42.07   0.000      4146731     4551997
     5#nonsurvivor  |   3982987   114853.8    34.68   0.000      3757877     4208096
        6#survivor  |   4319843   101352.1    42.62   0.000      4121196     4518489
     6#nonsurvivor  |   3919731   109546.7    35.78   0.000      3705023     4134438
        7#survivor  |   4291024   99738.81    43.02   0.000      4095539     4486508
     7#nonsurvivor  |   3861218   105832.5    36.48   0.000      3653790     4068646
        8#survivor  |   4262895   98459.08    43.30   0.000      4069919     4455872
     8#nonsurvivor  |   3807265   103359.7    36.84   0.000      3604684     4009847
        9#survivor  |   4235447   97436.08    43.47   0.000      4044475     4426418
     9#nonsurvivor  |   3757705   101817.4    36.91   0.000      3558147     3957263
       10#survivor  |   4208667   96604.98    43.57   0.000      4019324     4398009
    10#nonsurvivor  |   3712385   100954.1    36.77   0.000      3514518     3910251
       11#survivor  |   4182545   95914.01    43.61   0.000      3994557     4370533
    11#nonsurvivor  |   3671165   100587.2    36.50   0.000      3474018     3868313
       12#survivor  |   4157072   95324.86    43.61   0.000      3970238     4343905
    12#nonsurvivor  |   3633922   100604.6    36.12   0.000      3436741     3831104
       13#survivor  |   4132237   94812.71    43.58   0.000      3946407     4318066
    13#nonsurvivor  |   3600544   100961.1    35.66   0.000      3402664     3798424
       14#survivor  |   4108030   94366.07    43.53   0.000      3923076     4292984
    14#nonsurvivor  |   3570929     101673    35.12   0.000      3371654     3770205
       15#survivor  |   4084443   93986.31    43.46   0.000      3900233     4268653
    15#nonsurvivor  |   3544991   102811.6    34.48   0.000      3343484     3746498
       16#survivor  |   4061467   93687.26    43.35   0.000      3877843     4245090
    16#nonsurvivor  |   3522652     104495    33.71   0.000      3317845     3727458
       17#survivor  |   4039091   93494.67    43.20   0.000      3855845     4222338
    17#nonsurvivor  |   3503846   106878.9    32.78   0.000      3294367     3713325
       18#survivor  |   4017309   93445.45    42.99   0.000      3834159     4200459
    18#nonsurvivor  |   3488519   110144.9    31.67   0.000      3272639     3704399
       19#survivor  |   3996112   93586.77    42.70   0.000      3812685     4179538
    19#nonsurvivor  |   3476625   114486.6    30.37   0.000      3252236     3701015
       20#survivor  |   3975491   93974.68    42.30   0.000      3791304     4159678
    20#nonsurvivor  |   3468130   120095.1    28.88   0.000      3232748     3703512
       21#survivor  |   3955439    94672.3    41.78   0.000      3769885     4140993
    21#nonsurvivor  |   3463009   127145.9    27.24   0.000      3213807     3712210
       22#survivor  |   3935948   95747.44    41.11   0.000      3748287     4123610
    22#nonsurvivor  |   3461246   135789.6    25.49   0.000      3195104     3727389
--------------------------------------------------------------------------------

350 . marginsplot, ytitle("Cells per mmc")xlab(0(1)21) ylab(2000000(1000000)6000000)  yline(3000000, lcolor(red)) legend
    > (off)

      Variables that uniquely identify margins: b_day_malattia death_30

351 . margins ar.death_30, at(b_day_malattia=(0(1)21))  expression(exp(predict(xb))) asbal

    Contrasts of adjusted predictions               Number of obs     =      1,805

    Expression   : exp(predict(xb))

    1._at        : b_day_mala~a    =           0
                   death_30                      (asbalanced)
                   p_age2                        (asbalanced)
                   p_cardio                      (asbalanced)
                   p_obeso                       (asbalanced)
                   p_rene                        (asbalanced)

    2._at        : b_day_mala~a    =           1
                   death_30                      (asbalanced)
                   p_age2                        (asbalanced)
                   p_cardio                      (asbalanced)
                   p_obeso                       (asbalanced)
                   p_rene                        (asbalanced)

    3._at        : b_day_mala~a    =           2
                   death_30                      (asbalanced)
                   p_age2                        (asbalanced)
                   p_cardio                      (asbalanced)
                   p_obeso                       (asbalanced)
                   p_rene                        (asbalanced)

    4._at        : b_day_mala~a    =           3
                   death_30                      (asbalanced)
                   p_age2                        (asbalanced)
                   p_cardio                      (asbalanced)
                   p_obeso                       (asbalanced)
                   p_rene                        (asbalanced)

    5._at        : b_day_mala~a    =           4
                   death_30                      (asbalanced)
                   p_age2                        (asbalanced)
                   p_cardio                      (asbalanced)
                   p_obeso                       (asbalanced)
                   p_rene                        (asbalanced)

    6._at        : b_day_mala~a    =           5
                   death_30                      (asbalanced)
                   p_age2                        (asbalanced)
                   p_cardio                      (asbalanced)
                   p_obeso                       (asbalanced)
                   p_rene                        (asbalanced)

    7._at        : b_day_mala~a    =           6
                   death_30                      (asbalanced)
                   p_age2                        (asbalanced)
                   p_cardio                      (asbalanced)
                   p_obeso                       (asbalanced)
                   p_rene                        (asbalanced)

    8._at        : b_day_mala~a    =           7
                   death_30                      (asbalanced)
                   p_age2                        (asbalanced)
                   p_cardio                      (asbalanced)
```

```
                p_obeso                       (asbalanced)
                p_rene                        (asbalanced)

9._at         : b_day_mala~a    =           8
                death_30                      (asbalanced)
                p_age2                        (asbalanced)
                p_cardio                      (asbalanced)
                p_obeso                       (asbalanced)
                p_rene                        (asbalanced)

10._at        : b_day_mala~a    =           9
                death_30                      (asbalanced)
                p_age2                        (asbalanced)
                p_cardio                      (asbalanced)
                p_obeso                       (asbalanced)
                p_rene                        (asbalanced)

11._at        : b_day_mala~a    =          10
                death_30                      (asbalanced)
                p_age2                        (asbalanced)
                p_cardio                      (asbalanced)
                p_obeso                       (asbalanced)
                p_rene                        (asbalanced)

12._at        : b_day_mala~a    =          11
                death_30                      (asbalanced)
                p_age2                        (asbalanced)
                p_cardio                      (asbalanced)
                p_obeso                       (asbalanced)
                p_rene                        (asbalanced)

13._at        : b_day_mala~a    =          12
                death_30                      (asbalanced)
                p_age2                        (asbalanced)
                p_cardio                      (asbalanced)
                p_obeso                       (asbalanced)
                p_rene                        (asbalanced)

14._at        : b_day_mala~a    =          13
                death_30                      (asbalanced)
                p_age2                        (asbalanced)
                p_cardio                      (asbalanced)
                p_obeso                       (asbalanced)
                p_rene                        (asbalanced)

15._at        : b_day_mala~a    =          14
                death_30                      (asbalanced)
                p_age2                        (asbalanced)
                p_cardio                      (asbalanced)
                p_obeso                       (asbalanced)
                p_rene                        (asbalanced)

16._at        : b_day_mala~a    =          15
                death_30                      (asbalanced)
                p_age2                        (asbalanced)
                p_cardio                      (asbalanced)
                p_obeso                       (asbalanced)
                p_rene                        (asbalanced)

17._at        : b_day_mala~a    =          16
                death_30                      (asbalanced)
                p_age2                        (asbalanced)
                p_cardio                      (asbalanced)
                p_obeso                       (asbalanced)
                p_rene                        (asbalanced)

18._at        : b_day_mala~a    =          17
                death_30                      (asbalanced)
                p_age2                        (asbalanced)
                p_cardio                      (asbalanced)
                p_obeso                       (asbalanced)
                p_rene                        (asbalanced)

19._at        : b_day_mala~a    =          18
                death_30                      (asbalanced)
                p_age2                        (asbalanced)
                p_cardio                      (asbalanced)
                p_obeso                       (asbalanced)
                p_rene                        (asbalanced)

20._at        : b_day_mala~a    =          19
                death_30                      (asbalanced)
                p_age2                        (asbalanced)
                p_cardio                      (asbalanced)
                p_obeso                       (asbalanced)
                p_rene                        (asbalanced)

21._at        : b_day_mala~a    =          20
                death_30                      (asbalanced)
                p_age2                        (asbalanced)
                p_cardio                      (asbalanced)
                p_obeso                       (asbalanced)
                p_rene                        (asbalanced)

22._at        : b_day_mala~a    =          21
                death_30                      (asbalanced)
                p_age2                        (asbalanced)
                p_cardio                      (asbalanced)
                p_obeso                       (asbalanced)
                p_rene                        (asbalanced)
```

	df	chi2	P>chi2
death_30@_at			
(nonsurvivor vs survivor) 1	**1**	**1.28**	**0.2572**
(nonsurvivor vs survivor) 2	**1**	**2.61**	**0.1065**
(nonsurvivor vs survivor) 3	**1**	**4.54**	**0.0330**

(nonsurvivor vs survivor) 4	1	7.11	0.0077
(nonsurvivor vs survivor) 5	1	10.18	0.0014
(nonsurvivor vs survivor) 6	1	13.54	0.0002
(nonsurvivor vs survivor) 7	1	16.90	0.0000
(nonsurvivor vs survivor) 8	1	20.02	0.0000
(nonsurvivor vs survivor) 9	1	22.71	0.0000
(nonsurvivor vs survivor) 10	1	24.89	0.0000
(nonsurvivor vs survivor) 11	1	26.54	0.0000
(nonsurvivor vs survivor) 12	1	27.65	0.0000
(nonsurvivor vs survivor) 13	1	28.24	0.0000
(nonsurvivor vs survivor) 14	1	28.29	0.0000
(nonsurvivor vs survivor) 15	1	27.78	0.0000
(nonsurvivor vs survivor) 16	1	26.71	0.0000
(nonsurvivor vs survivor) 17	1	25.07	0.0000
(nonsurvivor vs survivor) 18	1	22.90	0.0000
(nonsurvivor vs survivor) 19	1	20.32	0.0000
(nonsurvivor vs survivor) 20	1	17.48	0.0000
(nonsurvivor vs survivor) 21	1	14.56	0.0001
(nonsurvivor vs survivor) 22	1	11.76	0.0006
Joint	5	36.28	0.0000

	Contrast	Delta-method Std. Err.	[95% Conf.	Interval]
death_30@_at				
(nonsurvivor vs survivor) 1	-186987.3	165026.5	-510433.4	136458.7
(nonsurvivor vs survivor) 2	-238952.2	148043.8	-529112.8	51208.26
(nonsurvivor vs survivor) 3	-286012.5	134174.5	-548989.8	-23035.31
(nonsurvivor vs survivor) 4	-328412.7	123198.5	-569877.3	-86948.04
(nonsurvivor vs survivor) 5	-366377.2	114836.6	-591452.7	-141301.6
(nonsurvivor vs survivor) 6	-400112	108747.9	-613254	-186970.1
(nonsurvivor vs survivor) 7	-429805.6	104546.9	-634713.9	-224897.4
(nonsurvivor vs survivor) 8	-455630	101837	-655226.8	-256033.1
(nonsurvivor vs survivor) 9	-477741.5	100248.3	-674224.5	-281258.4
(nonsurvivor vs survivor) 10	-496282.1	99469.25	-691238.2	-301325.9
(nonsurvivor vs survivor) 11	-511379.7	99265	-705935.6	-316823.9
(nonsurvivor vs survivor) 12	-523149.3	99483.78	-718133.9	-328164.6
(nonsurvivor vs survivor) 13	-531692.9	100055.1	-727797.3	-335588.5
(nonsurvivor vs survivor) 14	-537101	100983.7	-735025.4	-339176.6
(nonsurvivor vs survivor) 15	-539452.3	102341.5	-740038	-338866.6
(nonsurvivor vs survivor) 16	-538814.6	104257.9	-743156.4	-334472.9
(nonsurvivor vs survivor) 17	-535245	106907.9	-744780.5	-325709.5
(nonsurvivor vs survivor) 18	-528790.1	110497.1	-745360.3	-312219.8
(nonsurvivor vs survivor) 19	-519486.5	115244.5	-745361.6	-293611.4
(nonsurvivor vs survivor) 20	-507360.9	121364	-745229.9	-269491.8
(nonsurvivor vs survivor) 21	-492430.2	129047.6	-745358.9	-239501.5
(nonsurvivor vs survivor) 22	-474701.9	138455.3	-746069.3	-203334.5

```
352 . graph save "Graph" "/Users/Chiara/Documents/fileDO 15 Maggio/Marg_RBC.gph", replace
    (file /Users/Chiara/Documents/fileDO 15 Maggio/Marg_RBC.gph saved)

353 .
354 . *margins death_30, at(b_day_malattia=(0(1)30))  expression(exp(predict(xb)))
355 . *marginsplot, title("RBC kinetic day 0 to day 30 after onset") ytitle("RBC(log-scale)")xlab(0(5)30) ylab(2000000(5
    > 00000)6000000, angle(horizontal)) yline(3000000, lcolor(red)) legend(off)
356 . *margins ar.death_30, at(b_day_malattia=(0(1)30))  expression(exp(predict(xb)))
357 .
358 . ***combine*****
359 . gr combine "/Users/Chiara/Documents/fileDO 15 Maggio/Marg_RBC.gph" "/Users/Chiara/Documents/fileDO 15 Maggio/BOX_R
    > BC.gph", ycomm   xsize(8) ysize(4)

360 . graph save "Graph" "/Users/Chiara/Documents/fileDO 15 Maggio/Graph_RBC.gph", replace
    (file /Users/Chiara/Documents/fileDO 15 Maggio/Graph_RBC.gph saved)

361 .
362 .
363 . ****************************************************************************
364 . *** analisi  HGB    *****************************************************
365 . ****************************************************************************
366 . **** scatter no model
367 . use "/Users/Chiara/Documents/fileDO 15 Maggio/fileDO\coorte_long_emocromo_anemia_1.dta", clear

368 . *scatter hgb b_day_malattia  if death_30==1, mcolor(red) legend(off) title("HGb variation over time") xtitle("day
    > since onset") ytitle("HGb (g/dL)") xlab(0(5)30)|| scatter hgb b_day_malattia  if death_30==0, mcolor(green) legend
    > (off) ms(oh)
369 . *keep if hgb!=.
370 .
371 . *********Graph box**************************************
372 . graph box hgb, over(death_30, label(nolabel)) over(b_day_malattia) asyvars box(1, fcolor(navy))  nooutsides ytitle
    > (Hb (gr/dl)) yline(8, lcolor(red)) legend(off)

373 . graph save "Graph" "/Users/Chiara/Documents/fileDO 15 Maggio/BOX_Hb.gph", replace
    (file /Users/Chiara/Documents/fileDO 15 Maggio/BOX_Hb.gph saved)

374 .
375 . **** modelling
376 . use "/Users/Chiara/Documents/fileDO 15 Maggio/fileDO\coorte_long_emocromo_anemia_1.dta", clear

377 . mixed hgb_ln c.b_day_malattia##i.death_30 i.p_age2 i.p_cardio i.p_obeso i.p_rene || progr:b_day_malattia, cov(unst
    > r)

    Performing EM optimization:

    Performing gradient-based optimization:

    Iteration 0:   log likelihood =  1759.0037
    Iteration 1:   log likelihood =  1759.0077
    Iteration 2:   log likelihood =  1759.0077

    Computing standard errors:

    Mixed-effects ML regression                     Number of obs     =      1,805
    Group variable: progr                           Number of groups  =        379

                                                    Obs per group:
```

```
                                                             min =          1
                                                             avg =        4.8
                                                             max =         22

                                                Wald chi2(7)      =     207.52
Log likelihood =  1759.0077                     Prob > chi2       =     0.0000

------------------------------------------------------------------------------------------
                    hgb_ln |      Coef.   Std. Err.      z    P>|z|     [95% Conf. Interval]
---------------------------+--------------------------------------------------------------
            b_day_malattia |  -.0061723   .0006761    -9.13   0.000    -.0074975   -.0048471
                           |
                  death_30 |
               nonsurvivor |  -.0544592   .0296128    -1.84   0.066    -.1124992    .0035809
                           |
death_30#c.b_day_malattia  |
               nonsurvivor |  -.0032463   .0020197    -1.61   0.108    -.0072049    .0007122
                           |
                  1.p_age2 |  -.0602628   .0152214    -3.96   0.000    -.0900962   -.0304294
                1.p_cardio |  -.0184339   .0163211    -1.13   0.259    -.0504227    .0135549
                 1.p_obeso |   .0064551   .0288868     0.22   0.823     -.050162    .0630721
                  1.p_rene |  -.1946001   .0335085    -5.81   0.000    -.2602756   -.1289246
                     _cons |   2.690466   .0129946   207.04   0.000     2.664997    2.715935
------------------------------------------------------------------------------------------

------------------------------------------------------------------------------
  Random-effects Parameters  |   Estimate   Std. Err.     [95% Conf. Interval]
-----------------------------+------------------------------------------------
progr: Unstructured          |
         var(b_day_malattia) |   .0000603   8.54e-06      .0000457    .0000796
                   var(_cons) |    .021043   .0021668      .0171972    .0257487
   cov(b_day_malattia,_cons) |  -.0005393   .0001172     -.0007689   -.0003097
-----------------------------+------------------------------------------------
               var(Residual) |   .0040964   .0001641       .003787    .0044311
------------------------------------------------------------------------------
LR test vs. linear model: chi2(3) = 2144.88               Prob > chi2 = 0.0000

Note: LR test is conservative and provided only for reference.

378 . est store linear

379 . mixed hgb_ln  c.b_day_malattia##c.b_day_malattia##i.death_30 i.p_age2 i.p_obeso i.p_rene i.p_cardio || progr:b_day
    > _malattia, cov(unstr)

Performing EM optimization:

Performing gradient-based optimization:

Iteration 0:   log likelihood =  1760.7311
Iteration 1:   log likelihood =  1760.7359
Iteration 2:   log likelihood =  1760.7359

Computing standard errors:

Mixed-effects ML regression                     Number of obs     =      1,805
Group variable: progr                           Number of groups  =        379

                                                Obs per group:
                                                             min =          1
                                                             avg =        4.8
                                                             max =         22

                                                Wald chi2(9)      =     209.82
Log likelihood =  1760.7359                     Prob > chi2       =     0.0000

----------------------------------------------------------------------------------------------------------
                                    hgb_ln |      Coef.   Std. Err.      z    P>|z|     [95% Conf. Interval]
-------------------------------------------+--------------------------------------------------------------
                            b_day_malattia |  -.0089773   .0019427    -4.62   0.000    -.0127849   -.0051697
                                           |
          c.b_day_malattia#c.b_day_malattia |   .0001142   .0000741     1.54   0.123     -.000031    .0002593
                                           |
                                  death_30 |
                               nonsurvivor |  -.0496867   .0361198    -1.38   0.169    -.1204801    .0211067
                                           |
                 death_30#c.b_day_malattia |
                               nonsurvivor |  -.0049944    .004972    -1.00   0.315    -.0147393    .0047506
                                           |
death_30#c.b_day_malattia#c.b_day_malattia |
                               nonsurvivor |   .0000927   .0002034     0.46   0.648    -.0003059    .0004914
                                           |
                                  1.p_age2 |  -.0607034   .0152433    -3.98   0.000    -.0905797   -.0308271
                                 1.p_obeso |   .0075202   .0289389     0.26   0.795    -.0491989    .0642393
                                  1.p_rene |  -.1956897   .0335835    -5.83   0.000    -.2615123   -.1298672
                                1.p_cardio |  -.0181757   .0163432    -1.11   0.266    -.0502078    .0138564
                                     _cons |   2.705509   .0162643   166.35   0.000     2.673632    2.737387
----------------------------------------------------------------------------------------------------------

------------------------------------------------------------------------------
  Random-effects Parameters  |   Estimate   Std. Err.     [95% Conf. Interval]
-----------------------------+------------------------------------------------
progr: Unstructured          |
         var(b_day_malattia) |   .0000622   8.79e-06      .0000471     .000082
                   var(_cons) |   .0216148    .002242      .0176385    .0264875
   cov(b_day_malattia,_cons) |  -.0005742   .0001218      -.000813   -.0003355
-----------------------------+------------------------------------------------
               var(Residual) |   .0040683   .0001636        .00376    .0044018
------------------------------------------------------------------------------
LR test vs. linear model: chi2(3) = 2144.62               Prob > chi2 = 0.0000

Note: LR test is conservative and provided only for reference.

380 . est store quadratic

381 . lrtest linear quadratic

Likelihood-ratio test                                 LR chi2(2)  =      3.46
(Assumption: linear nested in quadratic)              Prob > chi2 =    0.1776
```

```
382 .
383 . *margins****************
384 . quiet: mixed hgb_ln c.b_day_malattia##i.death_30 i.p_age2 i.p_cardio i.p_obeso i.p_rene || progr:b_day_malattia, c
> ov(unstr)

385 . margins death_30, at(b_day_malattia=(0(1)21))  expression(exp(predict(xb))) asbal

Adjusted predictions                          Number of obs     =      1,805

Expression   : exp(predict(xb))

1._at        : b_day_mala~a    =           0
               death_30                      (asbalanced)
               p_age2                        (asbalanced)
               p_cardio                      (asbalanced)
               p_obeso                       (asbalanced)
               p_rene                        (asbalanced)

2._at        : b_day_mala~a    =           1
               death_30                      (asbalanced)
               p_age2                        (asbalanced)
               p_cardio                      (asbalanced)
               p_obeso                       (asbalanced)
               p_rene                        (asbalanced)

3._at        : b_day_mala~a    =           2
               death_30                      (asbalanced)
               p_age2                        (asbalanced)
               p_cardio                      (asbalanced)
               p_obeso                       (asbalanced)
               p_rene                        (asbalanced)

4._at        : b_day_mala~a    =           3
               death_30                      (asbalanced)
               p_age2                        (asbalanced)
               p_cardio                      (asbalanced)
               p_obeso                       (asbalanced)
               p_rene                        (asbalanced)

5._at        : b_day_mala~a    =           4
               death_30                      (asbalanced)
               p_age2                        (asbalanced)
               p_cardio                      (asbalanced)
               p_obeso                       (asbalanced)
               p_rene                        (asbalanced)

6._at        : b_day_mala~a    =           5
               death_30                      (asbalanced)
               p_age2                        (asbalanced)
               p_cardio                      (asbalanced)
               p_obeso                       (asbalanced)
               p_rene                        (asbalanced)

7._at        : b_day_mala~a    =           6
               death_30                      (asbalanced)
               p_age2                        (asbalanced)
               p_cardio                      (asbalanced)
               p_obeso                       (asbalanced)
               p_rene                        (asbalanced)

8._at        : b_day_mala~a    =           7
               death_30                      (asbalanced)
               p_age2                        (asbalanced)
               p_cardio                      (asbalanced)
               p_obeso                       (asbalanced)
               p_rene                        (asbalanced)

9._at        : b_day_mala~a    =           8
               death_30                      (asbalanced)
               p_age2                        (asbalanced)
               p_cardio                      (asbalanced)
               p_obeso                       (asbalanced)
               p_rene                        (asbalanced)

10._at       : b_day_mala~a    =           9
               death_30                      (asbalanced)
               p_age2                        (asbalanced)
               p_cardio                      (asbalanced)
               p_obeso                       (asbalanced)
               p_rene                        (asbalanced)

11._at       : b_day_mala~a    =          10
               death_30                      (asbalanced)
               p_age2                        (asbalanced)
               p_cardio                      (asbalanced)
               p_obeso                       (asbalanced)
               p_rene                        (asbalanced)

12._at       : b_day_mala~a    =          11
               death_30                      (asbalanced)
               p_age2                        (asbalanced)
               p_cardio                      (asbalanced)
               p_obeso                       (asbalanced)
               p_rene                        (asbalanced)

13._at       : b_day_mala~a    =          12
               death_30                      (asbalanced)
               p_age2                        (asbalanced)
               p_cardio                      (asbalanced)
               p_obeso                       (asbalanced)
               p_rene                        (asbalanced)

14._at       : b_day_mala~a    =          13
               death_30                      (asbalanced)
               p_age2                        (asbalanced)
               p_cardio                      (asbalanced)
               p_obeso                       (asbalanced)
```

```
                p_rene                        (asbalanced)

15._at       : b_day_mala~a    =         14
               death_30                       (asbalanced)
               p_age2                         (asbalanced)
               p_cardio                       (asbalanced)
               p_obeso                        (asbalanced)
               p_rene                         (asbalanced)

16._at       : b_day_mala~a    =         15
               death_30                       (asbalanced)
               p_age2                         (asbalanced)
               p_cardio                       (asbalanced)
               p_obeso                        (asbalanced)
               p_rene                         (asbalanced)

17._at       : b_day_mala~a    =         16
               death_30                       (asbalanced)
               p_age2                         (asbalanced)
               p_cardio                       (asbalanced)
               p_obeso                        (asbalanced)
               p_rene                         (asbalanced)

18._at       : b_day_mala~a    =         17
               death_30                       (asbalanced)
               p_age2                         (asbalanced)
               p_cardio                       (asbalanced)
               p_obeso                        (asbalanced)
               p_rene                         (asbalanced)

19._at       : b_day_mala~a    =         18
               death_30                       (asbalanced)
               p_age2                         (asbalanced)
               p_cardio                       (asbalanced)
               p_obeso                        (asbalanced)
               p_rene                         (asbalanced)

20._at       : b_day_mala~a    =         19
               death_30                       (asbalanced)
               p_age2                         (asbalanced)
               p_cardio                       (asbalanced)
               p_obeso                        (asbalanced)
               p_rene                         (asbalanced)

21._at       : b_day_mala~a    =         20
               death_30                       (asbalanced)
               p_age2                         (asbalanced)
               p_cardio                       (asbalanced)
               p_obeso                        (asbalanced)
               p_rene                         (asbalanced)

22._at       : b_day_mala~a    =         21
               death_30                       (asbalanced)
               p_age2                         (asbalanced)
               p_cardio                       (asbalanced)
               p_obeso                        (asbalanced)
               p_rene                         (asbalanced)
```

	Margin	Delta-method Std. Err.	z	P>|z|	[95% Conf.	Interval]
_at#death_30						
1#survivor	12.89765	.299634	43.04	0.000	12.31038	13.48493
1#nonsurvivor	12.21404	.3683134	33.16	0.000	11.49216	12.93592
2#survivor	12.81829	.2952045	43.42	0.000	12.2397	13.39688
2#nonsurvivor	12.09954	.3534449	34.23	0.000	11.4068	12.79228
3#survivor	12.73942	.2910505	43.77	0.000	12.16897	13.30987
3#nonsurvivor	11.98612	.3399792	35.26	0.000	11.31977	12.65246
4#survivor	12.66103	.2871729	44.09	0.000	12.09818	13.22388
4#nonsurvivor	11.87375	.3279857	36.20	0.000	11.23091	12.51659
5#survivor	12.58312	.2835722	44.37	0.000	12.02733	13.13891
5#nonsurvivor	11.76245	.3175285	37.04	0.000	11.1401	12.38479
6#survivor	12.50569	.2802483	44.62	0.000	11.95642	13.05497
6#nonsurvivor	11.65218	.3086617	37.75	0.000	11.04721	12.25715
7#survivor	12.42874	.2772006	44.84	0.000	11.88544	12.97205
7#nonsurvivor	11.54295	.3014249	38.29	0.000	10.95217	12.13373
8#survivor	12.35226	.2744277	45.01	0.000	11.8144	12.89013
8#nonsurvivor	11.43474	.2958389	38.65	0.000	10.85491	12.01457
9#survivor	12.27626	.2719278	45.15	0.000	11.74329	12.80923
9#nonsurvivor	11.32755	.2919019	38.81	0.000	10.75543	11.89966
10#survivor	12.20072	.2696983	45.24	0.000	11.67212	12.72932
10#nonsurvivor	11.22136	.2895872	38.75	0.000	10.65378	11.78894
11#survivor	12.12564	.2677359	45.29	0.000	11.60089	12.6504
11#nonsurvivor	11.11616	.2888433	38.49	0.000	10.55004	11.68229
12#survivor	12.05103	.2660369	45.30	0.000	11.52961	12.57245
12#nonsurvivor	11.01196	.2895953	38.03	0.000	10.44436	11.57955
13#survivor	11.97688	.2645965	45.26	0.000	11.45828	12.49548
13#nonsurvivor	10.90872	.2917483	37.39	0.000	10.33691	11.48054
14#survivor	11.90318	.2634096	45.19	0.000	11.38691	12.41945
14#nonsurvivor	10.80646	.2951927	36.61	0.000	10.2279	11.38503
15#survivor	11.82994	.2624705	45.07	0.000	11.3155	12.34437
15#nonsurvivor	10.70516	.2998091	35.71	0.000	10.11754	11.29277
16#survivor	11.75714	.2617727	44.91	0.000	11.24408	12.27021
16#nonsurvivor	10.6048	.3054733	34.72	0.000	10.00609	11.20352
17#survivor	11.6848	.2613094	44.72	0.000	11.17264	12.19696
17#nonsurvivor	10.50539	.3120619	33.66	0.000	9.89376	11.11702
18#survivor	11.6129	.2610731	44.48	0.000	11.10121	12.12459
18#nonsurvivor	10.40691	.319455	32.58	0.000	9.780788	11.03303
19#survivor	11.54144	.261056	44.21	0.000	11.02978	12.0531
19#nonsurvivor	10.30935	.3275394	31.48	0.000	9.667384	10.95132
20#survivor	11.47042	.2612501	43.91	0.000	10.95838	11.98247
20#nonsurvivor	10.21271	.3362104	30.38	0.000	9.553746	10.87167
21#survivor	11.39984	.2616468	43.57	0.000	10.88703	11.91266
21#nonsurvivor	10.11697	.3453723	29.29	0.000	9.440051	10.79389
22#survivor	11.3297	.2622374	43.20	0.000	10.81572	11.84367
22#nonsurvivor	10.02213	.3549391	28.24	0.000	9.32646	10.7178

```
386 . marginsplot, ytitle("HGb (g/dL") xlab(0(1)21) ylab(5(5)20) yline(8, lcolor(red)) legend(off)

      Variables that uniquely identify margins: b_day_malattia death_30

387 . margins ar.death_30, at(b_day_malattia=(0(1)21))  expression(exp(predict(xb))) asbal

    Contrasts of adjusted predictions              Number of obs     =      1,805

    Expression   : exp(predict(xb))

    1._at        : b_day_mala~a    =           0
                   death_30                      (asbalanced)
                   p_age2                        (asbalanced)
                   p_cardio                      (asbalanced)
                   p_obeso                       (asbalanced)
                   p_rene                        (asbalanced)

    2._at        : b_day_mala~a    =           1
                   death_30                      (asbalanced)
                   p_age2                        (asbalanced)
                   p_cardio                      (asbalanced)
                   p_obeso                       (asbalanced)
                   p_rene                        (asbalanced)

    3._at        : b_day_mala~a    =           2
                   death_30                      (asbalanced)
                   p_age2                        (asbalanced)
                   p_cardio                      (asbalanced)
                   p_obeso                       (asbalanced)
                   p_rene                        (asbalanced)

    4._at        : b_day_mala~a    =           3
                   death_30                      (asbalanced)
                   p_age2                        (asbalanced)
                   p_cardio                      (asbalanced)
                   p_obeso                       (asbalanced)
                   p_rene                        (asbalanced)

    5._at        : b_day_mala~a    =           4
                   death_30                      (asbalanced)
                   p_age2                        (asbalanced)
                   p_cardio                      (asbalanced)
                   p_obeso                       (asbalanced)
                   p_rene                        (asbalanced)

    6._at        : b_day_mala~a    =           5
                   death_30                      (asbalanced)
                   p_age2                        (asbalanced)
                   p_cardio                      (asbalanced)
                   p_obeso                       (asbalanced)
                   p_rene                        (asbalanced)

    7._at        : b_day_mala~a    =           6
                   death_30                      (asbalanced)
                   p_age2                        (asbalanced)
                   p_cardio                      (asbalanced)
                   p_obeso                       (asbalanced)
                   p_rene                        (asbalanced)

    8._at        : b_day_mala~a    =           7
                   death_30                      (asbalanced)
                   p_age2                        (asbalanced)
                   p_cardio                      (asbalanced)
                   p_obeso                       (asbalanced)
                   p_rene                        (asbalanced)

    9._at        : b_day_mala~a    =           8
                   death_30                      (asbalanced)
                   p_age2                        (asbalanced)
                   p_cardio                      (asbalanced)
                   p_obeso                       (asbalanced)
                   p_rene                        (asbalanced)

    10._at       : b_day_mala~a    =           9
                   death_30                      (asbalanced)
                   p_age2                        (asbalanced)
                   p_cardio                      (asbalanced)
                   p_obeso                       (asbalanced)
                   p_rene                        (asbalanced)

    11._at       : b_day_mala~a    =          10
                   death_30                      (asbalanced)
                   p_age2                        (asbalanced)
                   p_cardio                      (asbalanced)
                   p_obeso                       (asbalanced)
                   p_rene                        (asbalanced)

    12._at       : b_day_mala~a    =          11
                   death_30                      (asbalanced)
                   p_age2                        (asbalanced)
                   p_cardio                      (asbalanced)
                   p_obeso                       (asbalanced)
                   p_rene                        (asbalanced)

    13._at       : b_day_mala~a    =          12
                   death_30                      (asbalanced)
                   p_age2                        (asbalanced)
                   p_cardio                      (asbalanced)
                   p_obeso                       (asbalanced)
                   p_rene                        (asbalanced)

    14._at       : b_day_mala~a    =          13
                   death_30                      (asbalanced)
                   p_age2                        (asbalanced)
                   p_cardio                      (asbalanced)
                   p_obeso                       (asbalanced)
                   p_rene                        (asbalanced)
```

```
15._at       : b_day_mala~a    =          14
               death_30                      (asbalanced)
               p_age2                        (asbalanced)
               p_cardio                      (asbalanced)
               p_obeso                       (asbalanced)
               p_rene                        (asbalanced)

16._at       : b_day_mala~a    =          15
               death_30                      (asbalanced)
               p_age2                        (asbalanced)
               p_cardio                      (asbalanced)
               p_obeso                       (asbalanced)
               p_rene                        (asbalanced)

17._at       : b_day_mala~a    =          16
               death_30                      (asbalanced)
               p_age2                        (asbalanced)
               p_cardio                      (asbalanced)
               p_obeso                       (asbalanced)
               p_rene                        (asbalanced)

18._at       : b_day_mala~a    =          17
               death_30                      (asbalanced)
               p_age2                        (asbalanced)
               p_cardio                      (asbalanced)
               p_obeso                       (asbalanced)
               p_rene                        (asbalanced)

19._at       : b_day_mala~a    =          18
               death_30                      (asbalanced)
               p_age2                        (asbalanced)
               p_cardio                      (asbalanced)
               p_obeso                       (asbalanced)
               p_rene                        (asbalanced)

20._at       : b_day_mala~a    =          19
               death_30                      (asbalanced)
               p_age2                        (asbalanced)
               p_cardio                      (asbalanced)
               p_obeso                       (asbalanced)
               p_rene                        (asbalanced)

21._at       : b_day_mala~a    =          20
               death_30                      (asbalanced)
               p_age2                        (asbalanced)
               p_cardio                      (asbalanced)
               p_obeso                       (asbalanced)
               p_rene                        (asbalanced)

22._at       : b_day_mala~a    =          21
               death_30                      (asbalanced)
               p_age2                        (asbalanced)
               p_cardio                      (asbalanced)
               p_obeso                       (asbalanced)
               p_rene                        (asbalanced)
```

	df	chi2	P>chi2
death_30@_at			
(nonsurvivor vs survivor) 1	1	3.45	0.0631
(nonsurvivor vs survivor) 2	1	4.19	0.0407
(nonsurvivor vs survivor) 3	1	5.03	0.0250
(nonsurvivor vs survivor) 4	1	5.96	0.0146
(nonsurvivor vs survivor) 5	1	6.98	0.0082
(nonsurvivor vs survivor) 6	1	8.06	0.0045
(nonsurvivor vs survivor) 7	1	9.18	0.0025
(nonsurvivor vs survivor) 8	1	10.28	0.0013
(nonsurvivor vs survivor) 9	1	11.33	0.0008
(nonsurvivor vs survivor) 10	1	12.28	0.0005
(nonsurvivor vs survivor) 11	1	13.10	0.0003
(nonsurvivor vs survivor) 12	1	13.75	0.0002
(nonsurvivor vs survivor) 13	1	14.23	0.0002
(nonsurvivor vs survivor) 14	1	14.55	0.0001
(nonsurvivor vs survivor) 15	1	14.70	0.0001
(nonsurvivor vs survivor) 16	1	14.72	0.0001
(nonsurvivor vs survivor) 17	1	14.62	0.0001
(nonsurvivor vs survivor) 18	1	14.43	0.0001
(nonsurvivor vs survivor) 19	1	14.18	0.0002
(nonsurvivor vs survivor) 20	1	13.88	0.0002
(nonsurvivor vs survivor) 21	1	13.55	0.0002
(nonsurvivor vs survivor) 22	1	13.21	0.0003
Joint	3	19.01	0.0003

	Contrast	Delta-method Std. Err.	[95% Conf.	Interval]
death_30@_at				
(nonsurvivor vs survivor) 1	-.6836119	.3678441	-1.404573	.0373493
(nonsurvivor vs survivor) 2	-.7187483	.3511618	-1.407013	-.0304838
(nonsurvivor vs survivor) 3	-.7532997	.335995	-1.411838	-.0947616
(nonsurvivor vs survivor) 4	-.7872732	.3224432	-1.41925	-.1552961
(nonsurvivor vs survivor) 5	-.8206756	.3106033	-1.429447	-.2119043
(nonsurvivor vs survivor) 6	-.853514	.3005633	-1.442607	-.2644206
(nonsurvivor vs survivor) 7	-.8857951	.2923953	-1.458879	-.3127107
(nonsurvivor vs survivor) 8	-.9175257	.2861479	-1.478365	-.3566863
(nonsurvivor vs survivor) 9	-.9487126	.2818391	-1.501107	-.3963181
(nonsurvivor vs survivor) 10	-.9793623	.2794522	-1.527079	-.431646
(nonsurvivor vs survivor) 11	-1.009481	.2789336	-1.556181	-.4627814
(nonsurvivor vs survivor) 12	-1.039076	.280195	-1.588248	-.4899039
(nonsurvivor vs survivor) 13	-1.068153	.2831184	-1.623055	-.5132514
(nonsurvivor vs survivor) 14	-1.096719	.2875636	-1.660333	-.5331046
(nonsurvivor vs survivor) 15	-1.124779	.2933771	-1.699788	-.5497708
(nonsurvivor vs survivor) 16	-1.152341	.3004001	-1.741114	-.5635674
(nonsurvivor vs survivor) 17	-1.179409	.3084755	-1.78401	-.5748086

```
(nonsurvivor vs survivor) 18  |   -1.205991   .3174533     -1.828188   -.5837943
(nonsurvivor vs survivor) 19  |   -1.232092   .3271942     -1.873381   -.5908036
(nonsurvivor vs survivor) 20  |   -1.257719   .3375714     -1.919346   -.5960909
(nonsurvivor vs survivor) 21  |   -1.282876   .3484714     -1.965867   -.5998843
(nonsurvivor vs survivor) 22  |    -1.30757   .3597942     -2.012753   -.6023863
--------------------------------------------------------------------------------

388 . graph save "Graph" "/Users/Chiara/Documents/fileDO 15 Maggio/Marg_Hb.gph", replace
    (file /Users/Chiara/Documents/fileDO 15 Maggio/Marg_Hb.gph saved)

389 .
390 . *margins death_30, at(b_day_malattia=(0(1)30))  expression(exp(predict(xb)))
391 . *marginsplot, title("HGb kinetic day 0 to day 30 after onset") ytitle("HGb (g/dL; log-scale)") xlab(0(5)30) ylab(4
    > (2)16, angle(horizontal)) yline(10, lcolor(green)) yline(8, lcolor(orange)) yline(5, lcolor(red)) legend(off)
392 . *margins ar.death_30, at(b_day_malattia=(0(1)30))  expression(exp(predict(xb)))
393 .
394 . **combine*****
395 . gr combine "/Users/Chiara/Documents/fileDO 15 Maggio/Marg_Hb.gph" "/Users/Chiara/Documents/fileDO 15 Maggio/BOX_Hb
    > .gph", ycomm   xsize(8) ysize(4)

396 . graph save "Graph" "/Users/Chiara/Documents/fileDO 15 Maggio/Graph_Hb.gph", replace
    (file /Users/Chiara/Documents/fileDO 15 Maggio/Graph_Hb.gph saved)

397 .
    end of do-file

398 . log close
          name:  <unnamed>
           log:  /Users/Chiara/Documents/fileDO 15 Maggio/Kinetics Hematological .smcl
      log type:  smcl
     closed on:  19 May 2020, 17:05:04
--------------------------------------------------------------------------------
```
