# Supplementary material for: COVID-19 disease—Temporal analyses of complete blood count parameters over course of illness, and relationship to patient demographics and management outcomes in survivors and non-survivors: A longitudinal descriptive cohort study
Source: PLoS One. 2020 Dec 28;15(12):e0244129. doi: 10.1371/journal.pone.0244129 (PMC7769441; doi:10.1371/journal.pone.0244129)
Supplement: S2 File — (PDF) [file pone.0244129.s002.pdf]

S1. Model building and variable selection process

Patients feature		Bivariable analysis				Model 1				Model 2				Model 3			
		OR	95% CI		p-value	OR	95% CI		p-value	OR	95% CI		p-value	OR	95% CI		p-value
Age	<60<br>>60	4.21	1.82	9.77	0.001	2.58	1.02	6.48	0.044	2.56	1.02	6.40	0.044	2.59	1.04	6.45	0.042
Sex	female<br>male	0.82	0.41	1.65	0.5765	-	-	-	-	-	-	-	-	-	-	-	-
Diabetes	no<br>yes	3.28	1.51	7.13	0.005	1.21	0.49	3.00	0.68	1.22	0.50	3.00	0.665	-	-	-	-
Neoplasm	no<br>yes	1.59	0.44	5.70	0.498	-	-	-	-	-	-	-	-	-	-	-	-
Obesity	no<br>yes	3.89	1.51	10.04	0.01	4.93	1.68	14.52	0.004	4.88	1.68	14.16	0.004	5.13	1.81	14.50	0.002
Chronic renal failure	no<br>yes	9.23	3.49	24.36	0	5.13	1.63	16.10	0.005	5.01	1.72	14.61	0.003	5.20	1.80	14.97	0.002
COPD	no<br>yes	2.47	1.13	5.43	0.0331	0.95	0.37	2.42	0.909	-	-	-	-				
Cardiovascular disease	no<br>yes	4.46	2.25	8.86	0.001	2.71	1.23	5.96	0.013	2.70	0.01	0.06	0.013	2.79	1.29	6.03	0.009
P-value vs full model	-	-	-	-	-	0.9223				0.9815				0.9857			
